# Synergistic Two‐Color Photochemical Polymer Network Formation and Lithography

**DOI:** 10.1002/anie.202518815

**Published:** 2025-10-03

**Authors:** Jan Hobich, Xingyu Wu, Florian Feist, Willie Scheibel, Natalia Herdt, Paul Somers, Eva Blasco, Hatice Mutlu, Christopher Barner‐Kowollik

**Affiliations:** ^1^ Institute of Nanotechnology (INT) Karlsruhe Institute of Technology (KIT) Kaiserstraße 12 76131 Karlsruhe Germany; ^2^ Institute of Functional Interfaces (IFG) Karlsruhe Institute of Technology (KIT) Kaiserstraße 12 76131 Karlsruhe Germany; ^3^ Institute for Molecular Systems Engineering and Advanced Materials Heidelberg University im Neuenheimer Feld 225 69120 Heidelberg Germany; ^4^ Department of Chemistry Technical Polymer Chemistry Rheinland‐Pfälzische Technische Universität Kaiserslautern‐Landau (RPTU) Erwin‐Schrödinger‐Straße, Gebäude 52–54 67663 Kaiserslautern Germany; ^5^ Department of Polymer Chemistry Leibniz‐Institut für Verbundwerkstoffe GmbH Erwin‐Schrödinger‐Straße 58, Gebäude 58/Raum 523 67663 Kaiserslautern Germany; ^6^ School of Chemistry and Physics Centre for Materials Science Queensland University of Technology (QUT) 2 George Street Brisbane QLD 4000 Australia

**Keywords:** Photoswitches, Polymer network formation, Synergistic two‐color photochemistry, Two‐color laser lithography

## Abstract

We introduce synergistic two‐color lithography as an advanced wavelength‐gated strategy for spatially and temporally controlling polymer network formation. Our photoresist entails two photoswitches, i.e., diarylindenone epoxide (DIO) and strained azobenzene (SA), each activated at a judiciously selected wavelength, i.e., 375 or 430 nm. Under specific conditions of photon flux, simultaneous irradiation at both wavelengths induces a (3 + 2) cycloaddition between the photoactivated DIO′ and SA′ species, generating covalently crosslinked networks, whereas under these specifically determined conditions, single‐wavelength exposure does not induce solidification. Kinetic analysis highlights the potential of synergistic activation to enable advanced additive manufacturing. We implemented the two‐color activated covalent bond forming system in a dual‐laser lithographic platform enabling the fabrication of well‐defined structures, including segmented ring and butterfly architectures by simply activating and deactivating one of the colors of light.

## Graphical abstract

Two photoswitches, able to respond to disparate wavelengths, are integrated into multi‐functional polymer architectures to form a photoresist that responds to curing upon irradiation with two distinct monochromatic colors of light, enabling two‐color lithography.

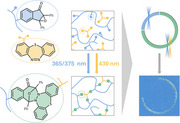

## Introduction

Three‐dimensional (3D) printing has evolved into a powerful platform for fabricating complex structures with high precision, particularly through light‐based techniques such as stereolithography (SLA), digital light processing (DLP), and two‐photon lithography (TPL).^[^ ^1^, ^2^, ^3^, ^4^, ^5^, ^6^ ^]^ Recently, the integration of multiple wavelengths of light into these systems has opened new dimensions of control in additive manufacturing.^[^ ^7^, ^8^, ^9^ ^]^ By employing distinct wavelengths, it becomes possible to not only finetune spatial and temporal control over the photochemical process, but also to unlock new fundamental photochemical reaction modes, which include synergistic, antagonistic, orthogonal, and cooperative mechanisms, each offering unique opportunities for designing photochemically addressable materials with enhanced structural complexity and function.^[^ ^8^, ^9^, ^10^ ^]^ Among those reaction modes, synergistic photochemistry stands out for its potential in rapidly constructing polymer networks with high precision.^[^ ^11^ ^]^ In such systems, a specific outcome–typically the formation of a covalent bond–occurs only when two distinct wavelengths of light intersect in space and time. This requirement introduces a dual‐gated mechanism that enables spatially confined and temporally controlled activation, minimizing undesired side reactions and enhancing pattern resolution. Indeed, this concept has already demonstrated considerable potential in projection‐based lithography and 3D microfabrication approaches.^[^ ^11^, ^12^, ^13^, ^14^, ^15^ ^]^

Very promising candidates for the development and realization of these systems are molecular photoswitches, i.e., molecules that are capable of undergoing reversible structural changes upon light exposure.^[^ ^16^, ^17^, ^18^, ^19^, ^20^ ^]^ Their tunable chemical structure enables precise modulation of their photochemical properties (absorption spectra and wavelength‐dependent photoreactivity), thereby facilitating the design of *λ*‐orthogonal activation windows.^[^ ^21^, ^22^, ^23^, ^24^, ^25^, ^26^ ^]^ Importantly, recent advances in the design of photoswitches with red‐shifted activation profiles and increased quantum yields have greatly expanded their usability, enabling their operation under mild and diverse lighting conditions, including throughout the visible spectrum and into the near‐infrared region. These features make them ideal building blocks for synergistic photochemical reactions, where selective excitation at non‐overlapping wavelengths is a prerequisite.^[^ ^17^, ^27^, ^28^, ^29^ ^]^

**Figure 1:**
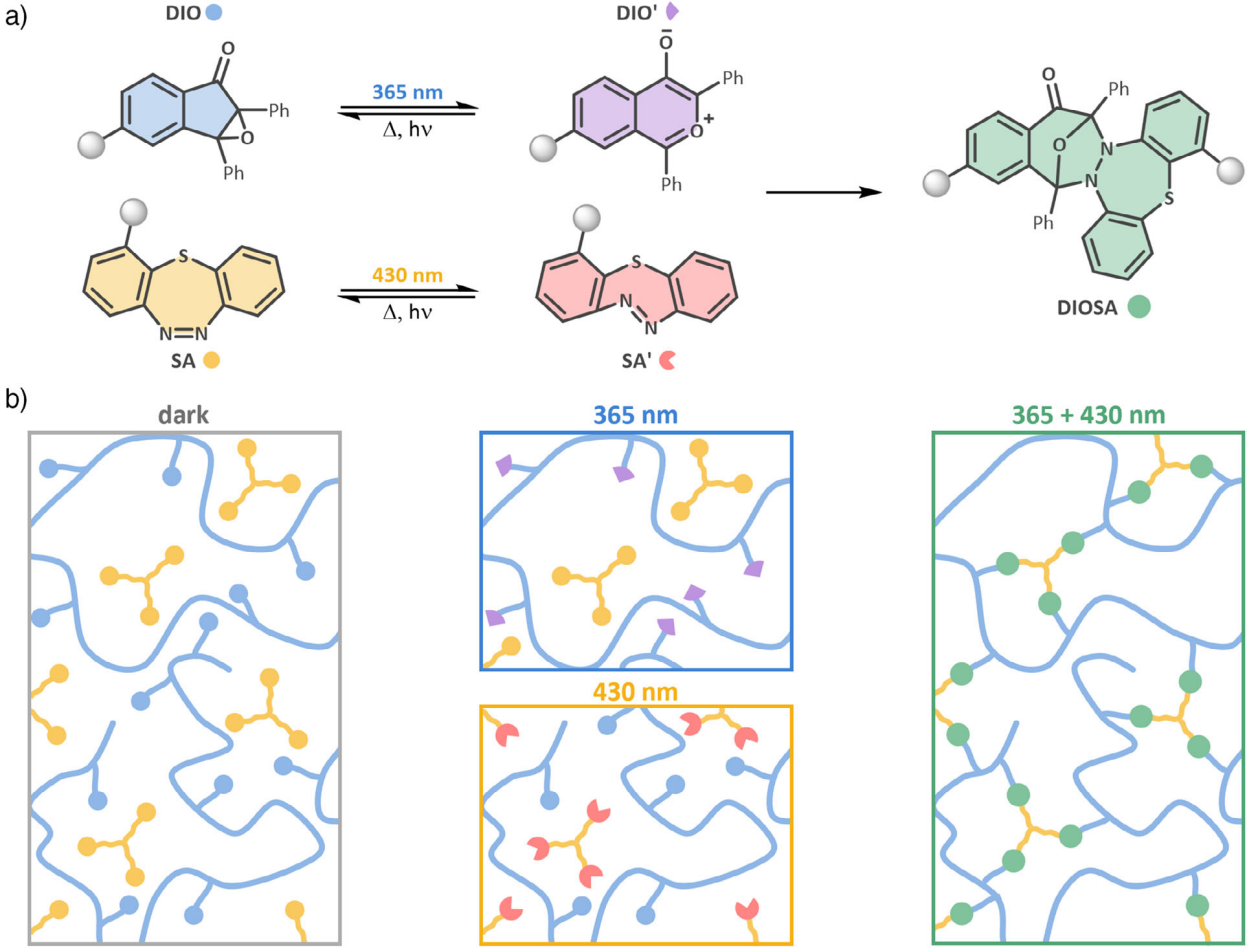
a) Light‐induced photoswitching of functionalized DIO (blue) and SA (yellow) to their corresponding isomers DIO’ (violet) and SA’ (red) under 365 and 430 nm irradiation, respectively. These activated isomers possess complementary reactivities and undergo a selective cycloaddition to form the DIOSA adduct (green). b) Photoresist consisting of a polymer with multiple DIO units (blue circles) and a molecule bearing three SA functionalities (yellow circles). One‐color irradiation with either 365 or 430 nm light reversibly and selectively switches DIO or SA to their reactive species DIO’ (violet triangles) and SA’ (red, three‐quarter circles) respectively. Simultaneous two‐color activation induces controlled crosslinking, forming a covalent network via DIOSA (green circles) formation. Color coding reflects the chemical species as defined in (a).

In an earlier study, we have reported a dual‐switch system tailored specifically for synergistic, two‐color covalent bond formation.^[^ ^30^ ^]^ Specifically, the two reagents are a diarylindenone epoxide (DIO) and a ring‐strained azobenzene derivative (SA) from the diazocine family.^[^ ^31^ ^]^ Upon exposure to ultraviolet (UV) light (365 nm), DIO undergoes a ring‐expansion to form a reactive isomer, DIO’,^[^ ^32^ ^]^ while SA isomerizes from a *cis* to a *trans* configuration under visible light (430 nm), yielding SA’.^[^ ^33^, ^34^, ^35^ ^]^ Importantly, the parent compounds DIO and SA remain inert in the dark, but their respective photogenerated isomers DIO’ and SA’ exhibit complementary reactivities: DIO’ engages in selective reactions with strained double bonds, and the *trans*‐configured SA’ features a highly strained azo bond that facilitates a strain‐release‐driven cycloaddition.^[^ ^32^, ^34^ ^]^ This unique combination results in a robust and selective bond formation, i.e., cycloaddition, yielding the covalent adduct, termed DIOSA (Figure 1a). The initial mechanistic study was carried out on the small‐molecule level, involving detailed structural and kinetic characterization via photochemical action plot measurements,^[^ ^36^ ^]^ single‐crystal X‐ray diffraction (SXRD), liquid chromatography‐mass spectrometry (LC‐MS), and nuclear magnetic resonance (NMR) spectroscopy. In addition, to further quantify the performance of this dual‐wavelength approach, we introduced a novel metric ϕ_*syn*_ to quantify the synergistic efficiency of the light‐triggered bond formation. In other words, ϕ_*syn*_ is instrumental in benchmarking synergistic multi‐color photochemical systems and provides a framework for evaluating spatial selectivity and light‐gated reactivity. Furthermore, we aimed to provide a fundamental basis for the development of advanced photoresists tailored for synergistic two‐color 3D printing.^[^ ^30^ ^]^

Building on these findings, the present work translates the small molecule dual‐switch system into a macromolecular setting suitable for synergistic two‐color network formation and lithography. Specifically, we report the design of a photoresist system composed of DIO and SA functional groups, engineered to undergo synergistic two‐color crosslinking (Figure 1b). Our current contribution thus marks a critical transition from fundamental photochemistry to application‐oriented materials science. By embedding both switches within polymer architectures, we enable controlled, dual‐light‐triggered polymer network formation suitable for use in lithographic fabrication. The performance of the system is demonstrated in lithographic patterning experiments, where one‐ and two‐color conditions are compared. Thus, our current study emphasizes the broader potential of wavelength‐encoded photochemistry in next‐generation 3D printing. The ability to trigger specific reactions using orthogonal light inputs not only provides improved control over polymer network formation but also paves the way for the fabrication of intricate architectures with high precision by harnessing dual wavelength‐gated activation. Our work makes a critical contribution to precision photochemistry and highlights how rational design of molecular switches can be leveraged for advanced additive manufacturing applications.^[^ ^37^ ^]^

## Results and Discussion

Our initial focus was the rational design and synthesis of a next‐generation photoresist containing the photoreactive core units DIO and SA. Subsequently, we examine the crosslinking behavior of the resulting macromolecular assemblies under dilute conditions (90 wt% solvent) using size‐exclusion chromatography (SEC), thereby establishing a mechanistic framework for understanding the key principles of synergistic polymer network formation. Finally, we validate the applicability of our introduced system by fabricating printed lines and other two‐dimensional patterns using a two‐color laser‐based lithography setup.

## Design of Photoresist Components

The initial step involved equipping both the DIO and SA photoswitch with synthetically versatile functional handles enabling their covalent incorporation into macromolecular scaffolds. For the DIO component, a modified synthetic route was developed, building on the established strategy from our previous reported approach^[^ ^30^ ^]^ in which a hydroxy functional group was introduced to the aromatic core via an alternative precursor (Supporting Information Section 2.1). Subsequent nucleophilic substitution with 6‐bromohexanol afforded a hydroxy‐terminated hexamethylene spacer (DIO‐OH), designed to enhance solubility, augment molecular flexibility, and mitigate proximal steric or electronic effects that could otherwise perturb the photoreactive core. Importantly, the terminal hydroxyl group also provided a strategic site for downstream functionalization.

The synthesis of the functional SA derivative (SA‐OH) was similarly refined (Supporting Information Section 2.2) from protocols for asymmetric diazocines.^[^ ^38^ ^]^ Structural modification was performed to incorporate a hydroxyhexyl linker distant to the azo bond (in the *ortho* position relative to the thioether moiety) ensuring minimal interference during isomerization and subsequent cycloaddition. Analogous to the DIO‐OH system, the flexible linker served to increase solubility, facilitate conformational flexibility, and spatially decouple the photoactive unit from its surrounding chemical environment.

Importantly, both modified photoswitches retained their reactivity under dual‐wavelength irradiation with DIO‐OH undergoing ring expansion to DIO′‐OH upon UV exposure (365 nm), and SA‐OH isomerizing to the reactive SA′‐OH form under visible light (430 nm), leading to the formation of DIOSA as corroborated by LC‐MS analysis (compare Supporting Information Section 3.2).

**Figure 2:**
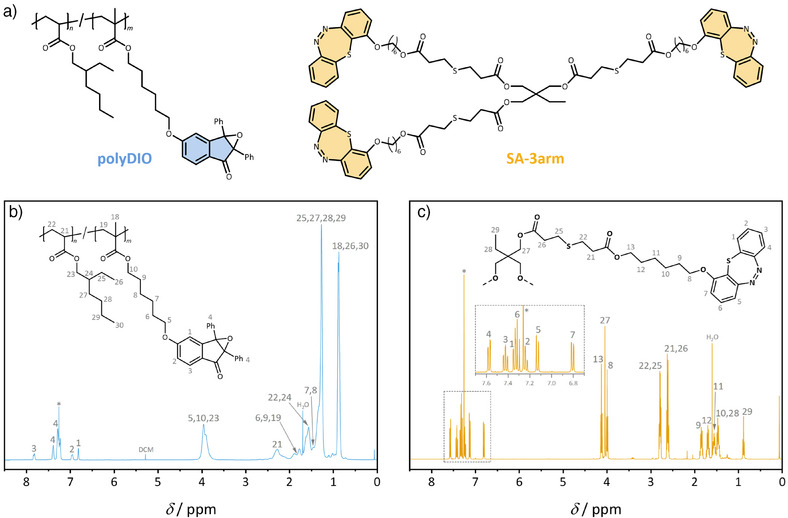
Structures and ^1^H NMR spectroscopic characterization of polyDIO and SA‐3arm. a) Chemical structures of DIO and SA moieties bearing functional handles, introduced into the molecular frameworks of polyDIO (blue) and SA‐3arm (yellow), respectively, to enable synergistic network formation. b) ^1^H NMR (400 MHz) spectrum of polyDIO (blue) in CDCl_3_ (*) at ambient temperature with proton assignment. Enlarged spectrum available in Figure S39. c) ^1^H NMR (400 MHz) spectrum of SA‐3arm (yellow) in CDCl_3_ (*) at ambient temperature with proton assignment. Enlarged spectrum available in Figure S64.

A critical design requirement for achieving photoinduced network formation via crosslinking lies in the multivalency of the reactive sites. In the present system, more than two photoactive groups per molecule are needed to enable crosslinking and, consequently, the formation of mechanically robust photoresist structures. We thus employ tailored synthetic strategies to embed multiple DIO and SA moieties within discrete macromolecular architectures, thereby facilitating the formation of a synergistically crosslinkable polymer network (Figure 2a).

The strategy underpinning our multi‐DIO compound was to tether the photoswitch directly onto the polymer backbone, enabling the facile incorporation of multiple DIO units per polymer chain. Using such a macromolecular precursor for network formation addresses the inherently slower reaction kinetics of step‐growth crosslinking, such as the reaction between DIO and SA, compared to the rapid radical‐initiated polymerizations typically used in 3D printing.^[^ ^39^ ^]^ This kinetic difference is associated with the fact that each crosslinking in the step‐growth process requires activation by two photons at separate sites, whereas in radical chain‐growth, a single photon can initiate numerous crosslinking events. A higher number of photoswitchable units per molecule means fewer crosslinking events and thus less photons are needed to form a stable network. Additionally, the above approach allows the use of an excess of DIO relative to SA, which was previously shown to enhance the synergistic efficiency in small‐molecule studies.^[^ ^30^ ^]^ Importantly, the overall crosslinking density remains unaffected as not all DIO units must participate in network formation for structural integrity to be achieved.

**Figure 3:**
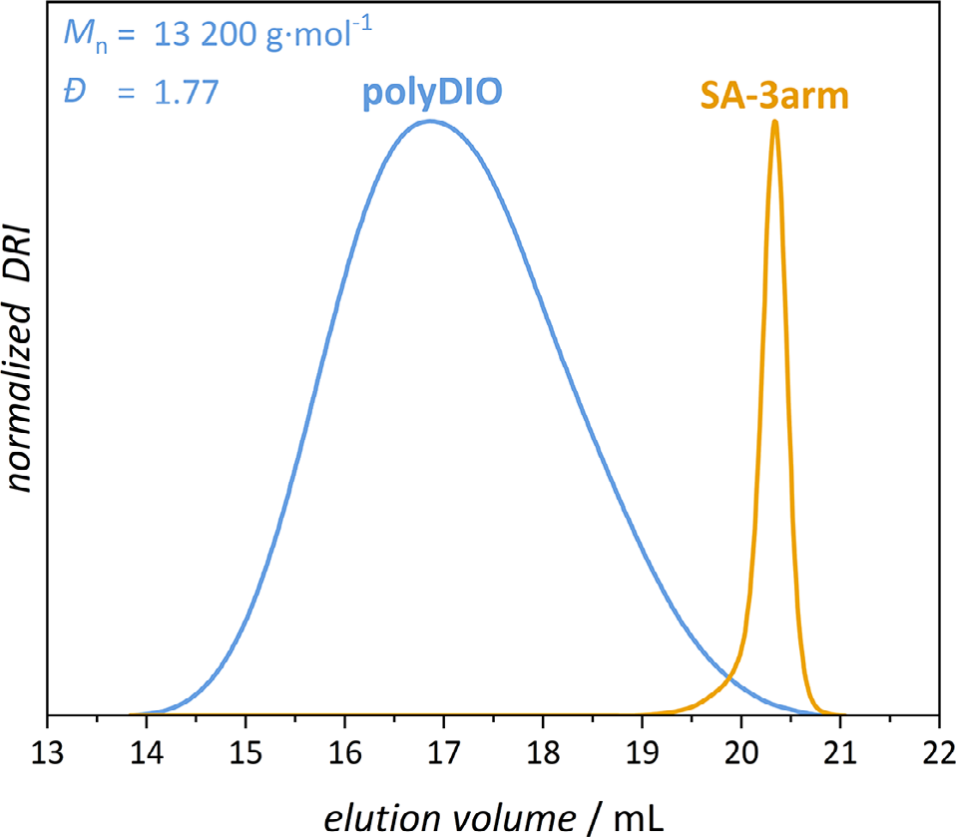
SEC elution traces of polyDIO (blue) and SA‐3arm (yellow). For polyDIO the corresponding *M*_n_ (13 200 g mol^−1^) and *Đ* (1.77) are shown in blue.

To functionalize the DIO‐based monomer to allow radical polymerization, the terminal hydroxy group was methacrylated. 2‐ethylhexyl acrylate (EHA) was selected as a comonomer to provide spatial separation between the DIO units along the polymer chain and to enhance solubility. Given its low glass transition temperature (*T*_g_ of −65 °C) in the homopolymer,^[^ ^40^ ^]^ EHA was also expected to mitigate brittleness and contribute to a soft, flexible polymer backbone, thereby potentially reducing the solvent requirements during photoresist formulation. Furthermore, the targeted number average molar mass (*M*_n_) of the polymer was carefully tuned, as it directly correlates with both *T*_g_ and viscosity,^[^ ^41^ ^]^ making it critical to find a balance between a sufficiently high *M*_n_ needed to incorporate multiple DIO units per chain, while being sufficiently low to ensure manageable processing viscosity. The monomers were copolymerized via free radical polymerization in toluene (1.5 M regarding total monomer concentration), initiated thermally by azobisisobutyronitrile (AIBN, 0.9 mol%) in the presence of 1‐dodecanthiol (1.2 mol%) as a chain transfer agent. We note that the use of reversible deactivation radical polymerization (RDRP) protocols is neither required nor desirable, as the introduced end groups (e.g., bromines or dithioesters) may be photolabile and interfere with the non‐radical network formation process. The resulting polymer (polyDIO, Figure 2a) was obtained with a moderate *M*_n_ of 13 200 g mol^−1^ and a dispersity (*Đ*) of 1.77, as determined by SEC (Figure 3). The content of the DIO monomer was quantified by ^1^H NMR spectroscopy and found to be close to 13.5 mol% (Supporting Information Section 6), in reasonable agreement with the initial monomer feed ratio of 15%. A *T*_g_ of −44 °C was determined by differential scanning calorimetry (DSC) (Figure S78), which is well suited for the intended formulation requirements for a soft and processable resist material.

We initially aimed to extend the radical polymerization strategy used for the DIO‐based monomer to the SA‐functionalized counterpart. However, polymerization attempts failed, as the SA‐unit was found to quench the propagating radicals (Supporting Information Section 5), which was an unexpected outcome given that azobenzene derivatives are typically considered inert under radical polymerization conditions.^[^ ^42^ ^]^ This may result from the azo bond or the thioether moiety being prone to radical attack due to the high ring strain of the SA unit. To overcome this limitation, the synthetic strategy was reoriented toward the synthesis of a discrete, three‐armed molecule, akin to pentaerythritol triacrylate, a well‐established crosslinking agent in photoresist formulations for light‐based 3D printing.^[^ ^39^, ^43^, ^44^ ^]^ Specifically, the hydroxy‐terminated SA compound (SA‐OH) was first acrylated, and then reacted with a trifunctional thiol via base‐mediated thiol‐Michael addition, employing 1,8‐diazabicyclo[5.4.0]undec‐7‐ene (DBU) as the catalyst, affording a well‐defined tri‐functional scaffold bearing terminal SA units (SA‐3arm, Figure 2a). The resulting compound was characterized by 1‐ and 2D‐NMR spectroscopy (Figures 2b and S64–68) in addition to SEC (Figure 3), confirming the successful incorporation of the photoswitchable moieties and establishing its suitability for crosslinking in synergistic two‐color photopolymer systems.

With both the DIO‐functional polymer and the three‐armed SA compound in hand, the subsequent step in developing a photoresist formulation involved identifying an appropriate solvent to ensure homogeneous mixing of all components. The selected solvent must offer robust solubilization for both photoactive species, exhibit photostability under irradiation, and not evaporate during the printing process; factors critical for preventing defects such as bubble formation, precipitation, or phase separation.^[^ ^45^ ^]^ Acetophenone emerged as an ideal candidate due to its high boiling point (201.7 °C)^[^ ^46^ ^]^ and its shared aromatic and polar character with the employed photoswitches, suggesting a high solubility of the functional components. Additionally, its successful use in comparable photochemical printing setups has been previously documented.^[^ ^47^, ^48^, ^49^, ^50^, ^51^ ^]^ Furthermore, control experiments employing the small‐molecule system confirmed that acetophenone sustains a high synergistic efficiency, thereby validating its selection as the solvent of choice for subsequent resist formulation (see Supporting Information, Section 3.3). The established photoreactivity thus provides a robust platform for transitioning the molecular system to the macromolecular scale, paving the way for practical material applications.

## Synergistic Network Formation

Building upon these foundational photochemical insights, we subsequently investigated whether the two‐color (i.e., *λ*‐orthogonal) reactivity could be harnessed effectively to achieve synergistic network formation under conditions that approximate those required for lithographic manufacturing. Our previous study investigating a small‐molecule model system enabled a precise kinetic analysis and detailed structural characterization of the dual‐wavelength photoreaction.^[^ ^30^ ^]^ The following experiments aim to bridge the gap between these controlled, idealized conditions and the complex environment of practical two‐color lithography. Accordingly, the crosslinkable compounds polyDIO and SA‐3arm were employed at elevated concentrations in a different solvent (i.e., acetophenone instead of acetonitrile) to assess synergistic network formation under conditions more representative of real‐world lithographic applications. This intermediate stage of investigation serves as a critical validation point for assessing the feasibility and robustness of synergistic two‐color crosslinking under formulation‐relevant settings.

As the formation of stable structures in lithographic fabrication requires both high solid content and sufficient crosslinking density, the concentrations of DIO and SA were increased from 5.0 mM (0.32 wt% solid content in acetonitrile) in the previous study to 47.5 mM (10.0 wt% solid content in acetophenone). Furthermore, the samples were irradiated for up to 3 h under a nitrogen atmosphere in the same dual‐LED setup (refer to Supporting Information Section 3.1) employed for the small molecule study under continuous stirring. While such long irradiation times exceed those typically used in practical printing, they allow for a robust assessment of the synergistic efficiency of the crosslinking under homogeneous and controlled conditions.

**Figure 4:**
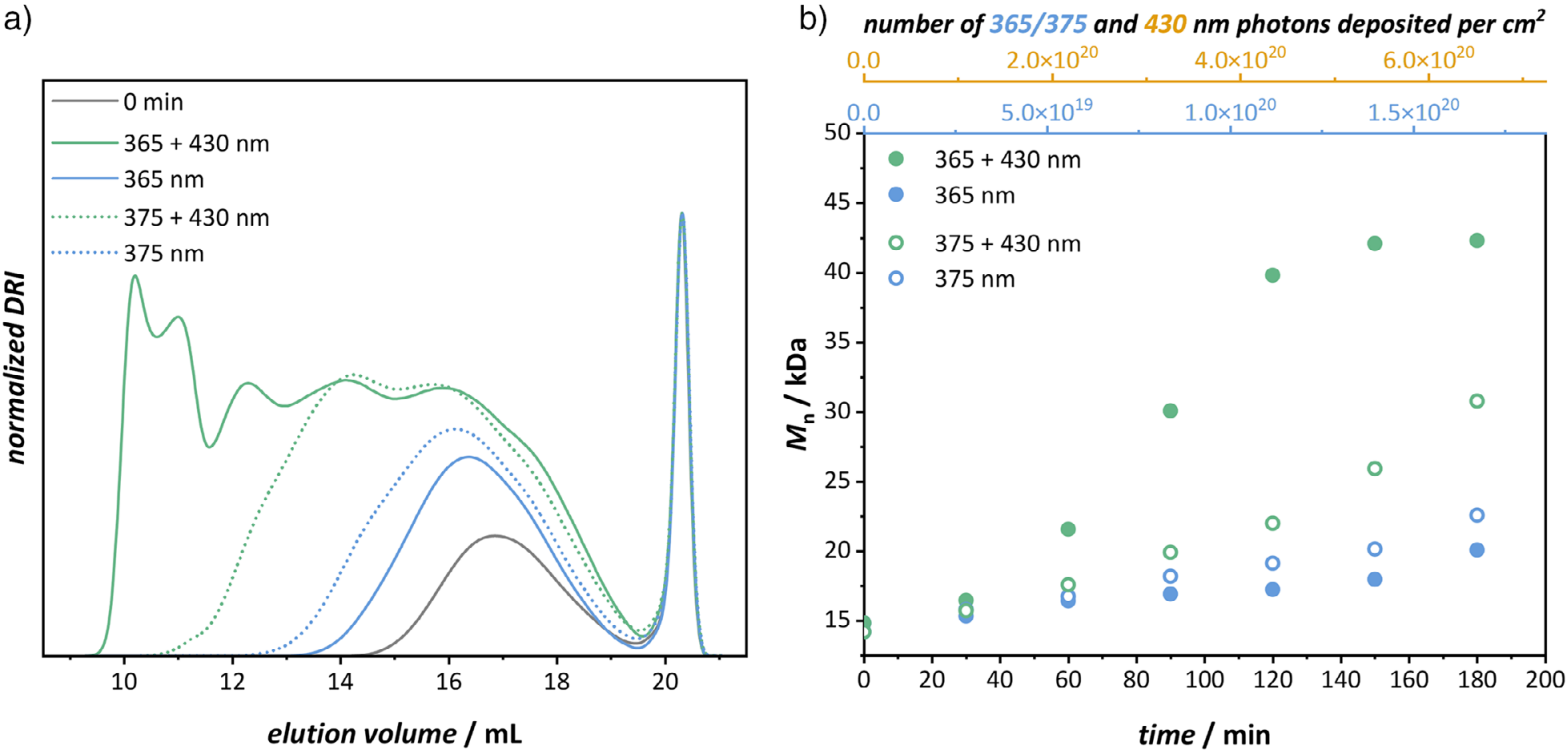
One‐ and two‐color crosslinking kinetics of polyDIO and SA‐3arm with comparison of 365 and 375 nm for the activation of DIO to DIO’. a) SEC traces of the crosslinking reactions between polyDIO and SA‐3arm in acetophenone before irradiation (grey), and after one‐ (365/375 nm, blue) and two‐color (365/375+430 nm, green) irradiation for 3 hours, respectively. b). Evolution of the polymer *M*_n_ during 3 h of irradiation under one‐ (365/375 nm, blue) and two‐color (365/375+430 nm, green) irradiation.

In contrast to the small‐molecule scale, network formation in the macromolecular system cannot be directly monitored using solution‐state techniques, such as NMR spectroscopy or LC‐MS, due to the formation of insoluble and crosslinked structures. To address this challenge, SEC was employed to qualitatively track the evolution of the soluble polymer fraction during the crosslinking reaction. As the crosslinking reaction progresses, SEC elution traces shift toward lower elution volumes, corresponding to an increase in molar mass over time (compare Figure 4a). The *M*_n_ of the soluble polymer fraction was used as a readout for the crosslinking progress, reflecting the gradual coupling of polyDIO and SA‐3arm moieties into a growing macromolecular network. It should be noted that the SEC results can only be used as qualitative references for comparison of the progress of network formation (more details are given in Supporting Information Section 3.4). The kinetics for one‐ and two‐color irradiations were conducted using the previously optimized wavelengths of 365 and 430 nm, applying photon fluxes of 1.55 × 10^1^⁶ photons s^−1^ cm^−^^2^ (365 nm) and 6.04 × 10^1^⁶ photons s^−1^ cm^−^^2^ (430 nm), under inert atmosphere and continuous stirring (Figure S2). Reaction kinetics were monitored for 3 h via SEC, with aliquots collected in 30 min intervals as seen in Figure 4 and Supporting Information Section 3.4.

The grey trace in Figure 4a shows the initial macromolecular formulation prior to irradiation, consisting of a broad polyDIO distribution at lower elution volumes (13.5 to 19.5 mL) and a more narrow distribution for SA‐3arm at higher elution volumes (19.5 to 20.8 mL). Upon irradiation with 365 nm light alone, a moderate increase in *M*_n_ from 14 900 to 20 100 g mol^−1^ and a shift toward lower elution volumes is observed, indicating partial crosslinking. In contrast, 430 nm light irradiation alone produced no significant shift in the SEC trace, even after 4 h (Figure S13), confirming its full orthogonality and suitability for synergistic dual‐wavelength experiments. Under simultaneous 365 and 430 nm exposure, the reaction proceeded significantly faster than for the 365 nm one‐color reaction. A pronounced increase in *M*_n_ was observed, reaching 42 300 g mol^−1^ after 3 h. Notably, a kinetic plateau of the *M*_n_ was reached after approximately 2 h as seen in Figure 4b, which likely reflects the formation of a largely insoluble network, as evidenced by the significant increase in resistance during syringe filtration, suggesting the onset of extensive network formation and loss of solubility. These findings confirm that the molecular‐level synergistic reactivity was successfully translated into the macromolecular context, enabling synergistic two‐color network formation.

For implementation in lithographic printing, the original 365 nm source had to be replaced. Although 430 nm irradiation was readily achievable using our current nanosecond (*ns*) pulsed optical parametric oscillator (OPO), simultaneously generating 365 nm was not feasible. Acquiring a second tunable laser to match the photon fluxes would be cost‐prohibitive. To circumvent this limitation, a continuous‐wave (CW) 375 nm laser, which is readily available and more affordable, was chosen as the closest functional alternative for the printing application.

To assess the impact of this wavelength substitution, one‐ and two‐color SEC kinetics were performed using a 375 nm LED calibrated to the same photon flux (1.55 × 10^1^⁶ photons s^−1^ cm^−^^2^) as for the original 365 nm LED. Intriguingly, SEC analysis revealed that the single‐wavelength exposure at 375 nm yielded a higher degree of crosslinking than observed at 365 nm (*M*_n_ =  22 600 versus 20 100 g mol^−1^). Meanwhile, the two‐color reaction (375 and 430 nm) preserved the synergistic effect, though the final *M*_n_ of 30 800 g mol^−1^ remained below that obtained under the combination of 365 and 430 nm light exposure (42 300 g mol^−1^).

The unexpected outcome for the one‐color reactions stands in contrast to the prior photochemical action plot studies, which had shown reduced reactivity of both photoswitches at 375 relative to 365 nm.^[^ ^30^ ^]^ Several factors may account for this discrepancy, including the use of acetophenone instead of acetonitrile as solvent, a nearly tenfold increase in photoswitch concentration (47.5 versus 5 mM), the incorporation of electron‐donating ether substituents to the aromatic core, wavelength‐dependent differences in penetration depth, and more complex photoswitch interplay, especially under network‐forming conditions. Although the precise influence of these individual variables cannot be conclusively identified, their collective influence likely accounts for the unexpectedly enhanced reactivity observed with 375 nm light exposure alone in comparison to 365 nm light exposure.

Despite the altered reactivity profile under 375 nm light exposure, narrowing the degree of synergistic efficiency, the hallmark of the dual‐wavelength system is clearly preserved with a significant increase in molar mass when simultaneously 375 and 430 nm light exposure is applied. These data confirm that synergistic network formation remains viable with the adapted conditions. Given the practical availability and technical constraints of the laser sources, the 375 and 430 nm wavelength combination was adopted for all subsequent lithography experiments, offering the optimal compromise between photochemical performance and technical feasibility with the tools at our disposal.

## Synergistic Two‐Color Lithography

To achieve synergistic two‐color lithography, it is essential to deliver two wavelengths with independently adjustable laser intensities simultaneously. Building on the demonstrated synergistic network formation of polyDIO and SA‐3arm under dual‐wavelength exposure at 375 and 430 nm, we incorporate a cost‐effective, readily available 375 nm CW laser into our custom‐designed monochromatic laser integrated stereolithographic apparatus (Mono LISA) 3D printer to enable synergistic two‐color lithography.^[^ ^50^ ^]^ Our Mono LISA is equipped with a tunable *ns* pulsed laser source, which enables easy selection of 430 nm. It is important to note that although our tunable pulsed laser covers a broad wavelength range (210–2400 nm), emitting two wavelengths simultaneously is not achievable with this single laser. An alternative configuration would be combining our Mono LISA printer, where 365 or 375 nm can be selected, with a 430 nm CW laser. However, due to the intrinsic photoreactivity of the system at 365 or 375 nm, efficient synergistic network formation within the nanosecond range requires an extremely high power 430 nm CW laser–an option which is technically challenging and prohibitively expensive. As illustrated in Scheme S1, the 375 nm CW laser, after leaving the optical fiber, is reflected at a beam splitter, while the 430 nm ns laser is transmitted through it. The combined beams are subsequently directed onto a two‐axis Galvo mirror system (*X* and *Y* axes) and ultimately focused onto the printing stage via a 45° mirror and a focusing lens. Upon importing G‐code files specifying the coordinates and travel speed into our customized software, the software processes these files to precisely control both the printing trajectory and speed, enabled by the Galvo mirror motion. The detailed specifications of the optical components and laser configurations of our setup as well as the mechanism of our two‐color lithography are provided in the Supporting Information Section 12.1.

**Figure 5:**
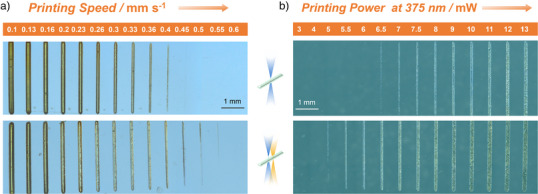
Optical microscopy images of lines printed with one‐ or two‐color exposure. Top lines were printed with one color, while bottom lines were printed using two colors as illustrated by the schematic in the middle. a) Lines printed at fixed laser powers at 375 nm (5 mW) and 430 nm (10 mW, bottom lines) with varying printing speeds. b) Lines printed at a fixed printing speed (0.6 mm s^−1^) and a 430 nm laser power (10 mW, bottom lines) with varying laser powers at 375 nm.

Taking full advantage of the versatility of our custom 3D printer–specifically, its capability for simultaneous 375 nm CW and tunable *ns* pulsed laser delivery, its wide range of accessible laser powers and its adjustable printing speed–we next implemented synergistic two‐color lithography using our custom‐designed photoswitch ink system, consisting of polyDIO (22.7 wt%) and SA‐3arm (7.4 wt%) with equimolar amounts of DIO and SA (70.5 mM, Figure S15) in acetophenone. Prior to synergistic two‐color lithography, we attempted laser printing with only polyDIO or SA‐3arm using 375, 430 nm or both wavelengths. As expected, no structure formation was observed under any accessible power or printing speed, confirming that DIO and SA cannot self‐react, and network formation requires both compounds. Similar results were observed when printing with the two‐component ink using only the 430 nm pulsed laser, which proves that DIO’ is not formed under 430 nm irradiation and is in accordance with the previously reported action plot.^[^ ^30^ ^]^ Subsequently, we systematically screened a range of printing speeds and laser powers at 375 and 430 nm to optimize the conditions for synergistic two‐color lithography. As shown in Figure 5, printing with our photoswitch ink is readily achievable. The features of the printed structures are in the mm‐range, with a minimal feature size of 48 µm. With fixed laser powers at 375 nm (5 mW) and 430 nm (10 mW), while varying the printing speed, microscopy images (Figure 5a) clearly show that the fabrication window of two‐color exposure in our experimental conditions is broader than that of one‐color, evidenced by the well‐defined lines formed at printing speeds of 0.45, 0.50 and 0.55 mm s^−1^ under dual‐wavelength exposure, whereas no lines are formed under these same conditions with single‐wavelength exposure. The expanded fabrication window under two‐wavelength exposure was also observed at other 375 nm laser powers (1, 2.5, and 15 mW), as detailed in Table S1. When varying the 375 nm laser power from 3 to 13 mW, with a fixed printing speed (0.6 mm s^−1^) and laser power at 430 nm (10 mW), single‐wavelength exposure failed to yield any well‐defined structures at 5.0, 5.5, 6.0 and 6.5 mW, whereas clear lines were obtained under two‐wavelength exposure at these powers (Figure 5b). These results are consistent with our findings in Figure 5a, where printing outcomes were governed by the delivered photon flux–achieved either by adjusting laser power or printing speed. Moreover, the fabrication windows broaden when the laser power at 375 nm increases for both one‐ and two‐color lithography (Table S1). It is also evident that the line widths increase as the laser power at 375 nm increases, or the printing speed decreases (Figure 5). We anticipate that higher photon flux, i.e., higher laser power or slower printing speed, drives more cycloadditions between DIO and SA, thereby generating more DIOSA crosslinks, which results in wider lines, maintained following development in acetone to remove the soluble constituents.

Inspired by our previous study, which demonstrated enhanced synergistic effects at higher powers at 430 nm, we investigated the effect of the laser power at 430 nm under a fixed laser power at 375 nm (5 mW) and printing speed (0.55 mm s^−1^). Interestingly, varying the power at 430 nm did not significantly affect the printed line widths (Figure S80), possibly associated with the pulsed nature of the 430 nm laser. We submit that the synergistic effect will be more pronounced when both wavelengths are delivered in either pulsed or continuous‐wave mode, a configuration not achievable at the current stage. Nevertheless, a clear synergistic effect is observed: Lines that cannot be printed with either 375 or 430 nm alone are readily formed when both wavelengths are applied simultaneously, highlighting the essential role of dual‐wavelength activation in triggering the synergistic network formation required for successful lithography.

**Figure 6:**
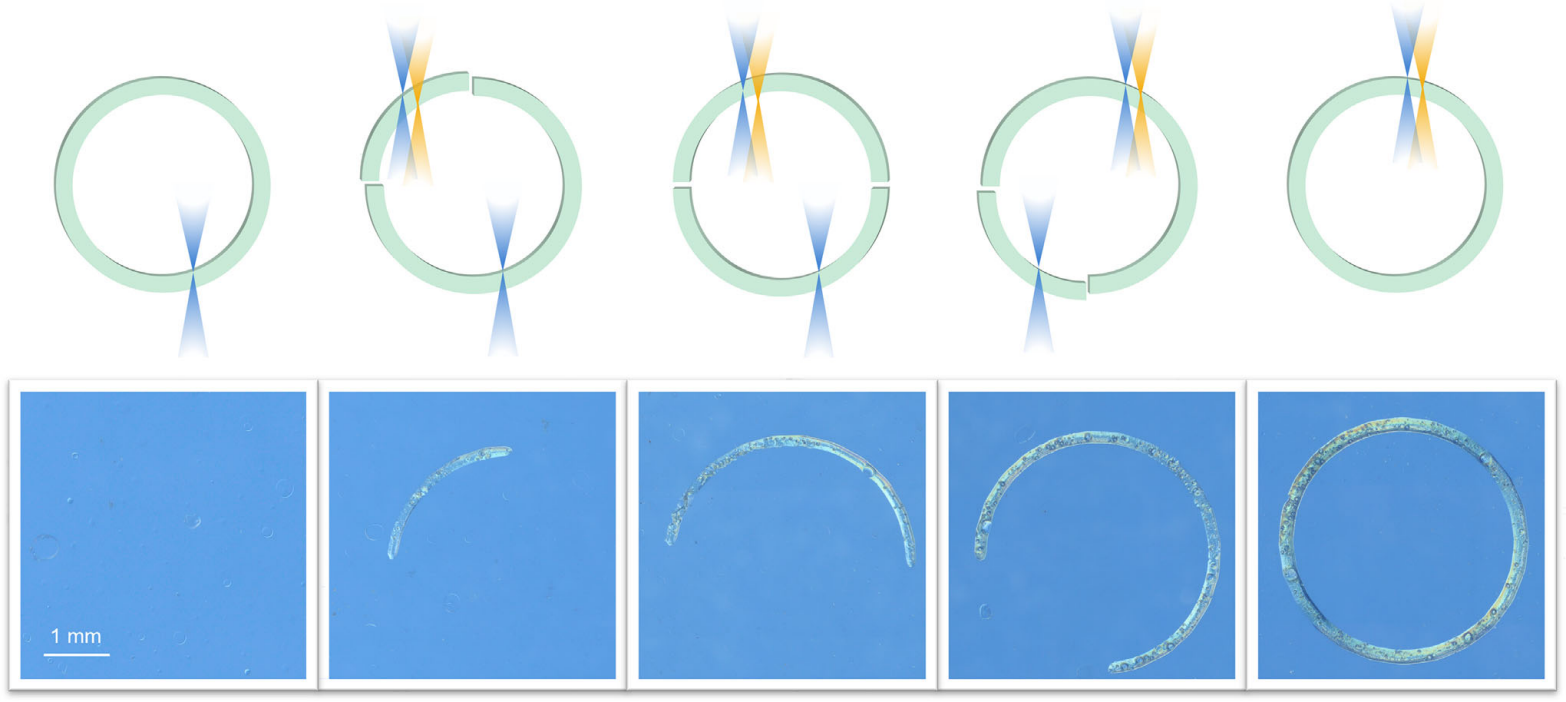
Optical microscopy images of a series of rings with varying fractions (100%, 75%, 50%, 25% and 0% from left to the right) printed using one‐color exposure (375 nm, 5 mW, 0.45 mm s^−1^). The remaining portions of each ring were printed using two‐color irradiation (375 nm at 5 mW and 430 nm at 10 mW, 0.45 mm s^−1^). The schematic at the top illustrates the segments irradiated with one‐ and two‐colors of light.

Encouraged by the results of the line tests, we proceeded to fabricate more intricate structures. A series of ring patterns were designed, with varying segments (100%, 75%, 50%, 25% and 0%) printed using one‐color exposure and the remaining portions printed with two‐color irradiation. The G‐codes for the rings and their trajectories are provided in Supporting Information 1 and Figure S81. The line tests provide optimized parameters for synergistic two‐color lithography: 375 nm at 5 mW with a printing speed of 0.45 mm s^−1^ for one‐color printing, while 375 nm (5 mW) combined with 430 nm (10 mW) at the same speed for two‐color printing. As shown in Figures 6 and S82, well‐defined ring segments were successfully printed when two wavelengths were employed, while those exposed to only a single wavelength failed to form, evidenced by the progressive formation of 0%, 25%, 50%, 75% and 100% rings from left to right, demonstrating the essential role of synergistic activation in polymer network formation.

**Figure 7:**
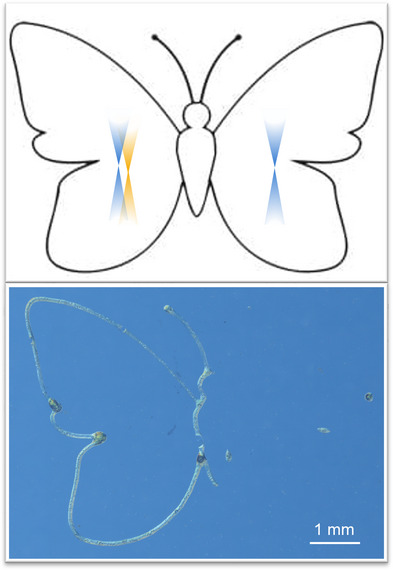
Butterfly structure, with the left half fabricated using two colors and the right half using only one color. Top: Schematic representation of the whole butterfly structure and the corresponding single‐ and dual‐irradiation applied. Bottom: Optical microscopy image of the butterfly with the left half fabricated using two colors (375 nm at 5 mW and 430 nm at 10 mW, 0.45 mm s^−1^) and the right half using only one color (375 nm at 5 mW, 0.45 mm s^−1^).

To further showcase the capabilities of our ink system and printer, a more complex curvy structure–a butterfly–was printed under the same optimized conditions. The left half of the butterfly was printed with two colors, while the right half was fabricated using only one color (375 nm). As illustrated in Figure 7, only the left half of the butterfly, i.e., the two‐color printed regions, formed reliably, emphasizing the excellent printability of our ink under simultaneous two‐color irradiation. The G‐code for the butterfly and its trajectory are provided in Supporting Information 2 and Figure S83. It is worth mentioning that a slightly rough surface texture on the printed structures was observed under microscope inspection. Although polyDIO and SA‐3arm exhibit excellent miscibility in acetophenone, they may form micelle‐like assemblies in solution that persist even after filtering the ink, indicating intrinsic nanoscale heterogeneity, potentially causing light scattering during printing.

For future work, synergistic two‐color printing offers potential for fabricating hollow and complex structures that are challenging, or even impossible to achieve with conventional 3D printing techniques, such as DLP or SLA.^[^ ^11^, ^12^ ^]^ The advantage arises from the unique requirement that polymer network formation only occurs when both wavelengths are simultaneously present. As a result, intricate architectures may be constructed with high spatial precision and resolution.

## Conclusion

We demonstrate the successful translation of a synergistic two‐color photochemical reaction from the small‐molecule level into a macromolecular photoresist system capable of advanced two‐color gated lithographic fabrication. By covalently integrating diarylindenone epoxide (DIO) and strained azobenzene (SA) photoswitches into defined multi‐functional polymer architectures, we achieve dual‐wavelength‐gated crosslinking. Systematic kinetic studies revealed that the synergistic interplay of 375 and 430 nm irradiation substantially accelerates network formation compared to single‐wavelength exposure, highlighting its potential for additive manufacturing. Implementation in a custom‐built two‐color laser lithography platform allows for the fabrication of intricate geometries in a synergistic manner under specifically determined conditions, such as segmented rings and asymmetric designs, demonstrating the versatility and robustness of our approach. The intrinsic dual‐gate mechanism provides a powerful means to suppress undesired crosslinking, opening pathways toward high‐precision, high resolution advanced printing. Our study thus establishes synergistic two‐color lithography as a promising strategy for next‐generation additive manufacturing, merging molecular‐level photochemical control with application‐oriented lithographic fabrication.

## Supporting Information

The authors have cited additional references within the Supporting Information.^[^ ^52^, ^53^ ^]^

## Conflict of Interests

The authors declare no conflict of interest.

## Supporting information



Supporting Information

Supporting Information

Supporting Information

Supporting Information

## Data Availability

The data that support the findings of this study are available in the supplementary material of this article.
