# Supplementary material for: Synergistic Two‐Color Photochemical Polymer Network Formation and Lithography
Source: Angew Chem Int Ed Engl. 2025 Oct 3;64(48):e202518815. doi: 10.1002/anie.202518815 (PMC12643345; doi:10.1002/anie.202518815)
Supplement: Supplementary file 1 — Supporting Information [file ANIE-64-e202518815-s001.pdf]

Supplementary Information

Synergistic Two-Color Photochemical Polymer Network Formation and Lithography

Jan Hobich,[1,2‡] Xingyu Wu,[1,2‡] Florian Feist,[1,2] Willie Scheibel,[1,2] Natalia Herdt,[1,2] Paul Somers,[1] Eva Blasco,[3] Hatice Mutlu,[4,5]* Christopher Barner-Kowollik[1,2,6]**

[1] Institute of Nanotechnology (INT), Karlsruhe Institute of Technology (KIT), Kaiserstraße 12, 76131 Karlsruhe, Germany.

[2] Institute of Functional Interfaces (IFG), Karlsruhe Institute of Technology (KIT), Kaiserstraße 12, 76131 Karlsruhe, Germany.

[3] Institute for Molecular Systems Engineering and Advanced Materials, Heidelberg University, im Neuenheimer Feld 225, Heidelberg 69120, Germany.

[4] Rheinland-Pfälzische Technische Universität Kaiserslautern-Landau (RPTU), Department of Chemistry, Technical Polymer Chemistry, Erwin-Schrödinger-Straße, Gebäude 52–54, 67663 Kaiserslautern, Germany.

[5] Leibniz-Institut für Verbundwerkstoffe GmbH, Department of Polymer Chemistry, Erwin-Schrödinger-Straße 58, Gebäude 58 / Raum 523, 67663 Kaiserslautern, Germany.

[6] School of Chemistry and Physics, Centre for Materials Science, Queensland University of Technology (QUT), 2 George Street, Brisbane, QLD 4000, Australia.

E-mail:

eva.blasco@uni-heidelberg.de, hatice.mutlu@rptu.de, christopher.barner-kowollik@kit.edu, christopher.barnerkowollik@qut.edu.au

Contents

1 Materials and Methods 4

1.1 Materials 4

1.2 Nuclear Magnetic Resonance (NMR) Spectroscopy 4

1.3 Liquid Chromatography-Mass Spectrometry (LC-MS) 4

1.4 Size Exclusion Chromatography (SEC) 5

1.5 Ultraviolet-Visible (UV-Vis) Spectroscopy 5

1.6 Attenuated Total -Reflectance Infrared Spectroscopy (ATR-IR) 6

1.7 Thermogravimetric Analysis (TGA) 6

1.8 Differential Scanning Calorimetry (DSC) 6

1.9 Flash Column Chromatography 6

1.10 Optical Microscopy 6

1.11 Light Emitting Diode (LED) Characterization 6

2 Synthesis 8

2.1 PolyDIO 8

2.2 SA-3arm 12

3 Two-color Photoreactions Using LED Lamps 16

3.1 Reaction setup 16

3.2 Small molecule test reactions 17

3.3 Testing acetophenone as solvent 21

3.4 SEC-tracked crosslinking 22

4 NMR Spectra of the Photoresists 26

5 Polymerization Quenching Effect of SA 28

6 Incorporation Ratio of DIO in PolyDIO 30

7 NMR Spectra 31

8 LC-MS Measurements 61

9 UV-Vis Spectra 63

10 ATR-IR Spectra 65
11 Thermal Characterization 66
12 Two-color Lithography 67
12.1 Setup 67
12.2 Sample preparation and lithography 68
12.3 Line testing 69
12.4 Intricate structures 70
13 References 72

1 Materials and Methods

1.1 Materials

The following chemicals were used as received without further purification:

Acryloyl chloride (96%, Alfa Aesar), 2-Aminothiophenol (98%, Thermo Fisher Scientific), Ammonium chloride (99%, Carl Roth), 2-Bromo-3-nitrophenol (98%, BLD Pharm), 6-Bromohexan-1-ol (98.2%, BLD Pharm), 2-Bromo-4-hydroxybenzaldehyde (98.2%, Ambeed), 1,8-Diazabicyclo[5.4.0]undec-7-ene (98%, Merck), Diphenylacetylene (99.9%, BLD Pharm), 1-Dodecanethiol (98%, Alfa Aesar), Hydrogen peroxide (30%, Carl Roth), Iron(III) chloride hexahydrate (99%, Carl Roth), Methacryloyl chloride (97%, Sigma Aldrich), Palladium(II) acetate (99.9%, BLD Pharm), Potassium carbonate (99%, Alfa Aesar), Potassium hydroxide (85%, Carl Roth), Sodium acetate (99%, Acros Organics), Sodium bicarbonate (99%, Carl Roth), Sodium hydroxide (98%, Carl Roth), Sodium sulfate (99%, Carl Roth), Tetrabutylammonium chloride (95%, BLD Pharm), Triethylamine (99%, Sigma Aldrich), Trimethylolpropane tris(3-mercaptopropionate) (95%, Sigma Aldrich), Zinc powder (99.9%, Thermo Fisher Scientific), Acetonitrile (HPLC grade, VWR), Acetophenone (99%, Sigma Aldrich), Dichloromethane (99%, GPR Rectapur), Dimethylformamide (99.8%, anhydrous, Thermo Fisher Scientific), Ethanol (99.5%, GPR Rectapur), Tetrahydrofuran (99.9%, anhydrous, Thermo Fisher Scientific), Tetrahydrofuran (HPLC grade, Carl Roth), Cyclohexane (reagent grade, VWR), Ethyl acetate (reagent grade, VWR), Toluene (HPLC grade, Carl Roth), CD_3CN (99.8%, Eurisotop), $CDCl_3$ (99.5%, Eurisotop), DMSO-d_6 (99.8%, Eurisotop).

1.2 Nuclear Magnetic Resonance (NMR) Spectroscopy

^{1}H- and ^{13}C-NMR spectra of the compounds were recorded on a *Bruker* AM 400, equipped with a 5 mm BBO-Probe, (^{1}H: 400 MHz, ^{13}C: 101 MHz). The δ-scale was normalized relative to the magnetic resonance arising from the residual solvent signal of $CDCl_3$ (δ = 7.26 ppm), CD_3CN (δ = 1.94 ppm) and DMSO-d_6 (δ = 2.50 ppm) for ^{1}H spectra, and $CDCl_3$ (δ = 77.16 ppm), CD_3CN (δ = 118.26 ppm) and DMSO-d_6 (δ = 39.52 ppm) for ^{13}C spectra, respectively.[1] The multiplicities were reported using the following abbreviations: s for singlet, d for doublet, t for triplet, q for quartet, p for pentet, m for multiplet and br for broad signal.

1.3 Liquid Chromatography-Mass Spectrometry (LC-MS)

LC-MS measurements were performed on an *Agilent* 1260 Infinity II system consisting of a quaternary pump (GB7111B), autosampler (G7129A, 100 µL sample loop), a temperature-controlled column oven (G7114A) and a variable UV-Vis detector (G7114A, VWD, flow cell G7114A 018, d = 10 mm, V = 14 µL). Separation was performed on a C18 HPLC column

(*Agilent* Poroshell 120 EC-C18 4.6x100 mm, 2.7 μm) operating at 40 °C. A gradient of acetonitrile:H_2O 45:55 – 100:0 (v/v) (additive 10 mmol L^{-1} ammonium formate) at a flow rate of 1 mL min^{-1} during 9 min was used as the eluting solvent. The flow was directed into an *Agilent* MSD (G613BA, AP-ESI ion source). The instrument was calibrated in the *m*/*z* range 118-2121 in positive mode and 113-2233 in the negative mode, using a premixed calibration solution (*Agilent*). The following parameters were used: spray chamber flow: 12 L min^{-1}, drying temperature: 350 K, Capillary Voltage: 3000 V, Fragmentor Voltage: 100 V.

1.4 Size Exclusion Chromatography (SEC)

The SEC measurements were conducted on a *PSS* SECurity2 system containing a *PSS* SECurity Degasser, *PSS* SECurity TCC6000 Column Oven (35 °C for THF), *PSS* SDV Column Set (8x50 mm 5 μm precolumn, 8x300 mm 5 μm analytical columns, 100000 Å and 1000 Å) and an *Agilent* 1260 Infinity II Isocratic Pump, Agilent 1260 Infinity II Standard Autosampler, *Agilent* 1260 Infinity II Diode Array and Multiple Wavelength Detector, *Agilent* 1260 Infinity II Refractive Index Detector (35 °C). HPLC grade THF was used as eluting solvents at a flow rate of 1 mL min^{-1}. Narrow disperse linear poly(methyl methacrylate) (PMMA) standards (M_n 831 $g \cdot mol^{-1}$ to $1.89 \cdot 10^6$ $g \cdot mol^{-1}$, PSS ReadyCal) were used as a calibrant. Molecular weight and dispersity analyses were performed via the PSS WinGPC UniChrom software (version 8.2). The polymer samples were dissolved in THF (~2 mg mL^{-1}) at ambient temperature and filtered through a 0.2 μm PTFE syringe filter.

1.5 Ultraviolet-Visible (UV-Vis) Spectroscopy

a) UV-Vis spectra for SA-OH, DIO-OH and polyDIO were recorded on an *Agilent* Cary 5000 UV-VIS-NIR spectrophotometer. Measurements were performed at ambient temperature using a *Hellma Analytics* quartz high precision cell (*108-F-10-40*) with a path length of 10 mm. Samples were prepared in acetonitrile or acetophenone, filtered through a 0.2 μm PTFE syringe filter and measured at ambient temperature.
b) UV-Vis spectra of the photoswitching of SA-3arm were recorded with a different setup due to the extremely short half-life times of the SA species in the millisecond range.[2] An *Ocean Insight* DH-2000 UV-VIS-NIR Light Source was coupled via optic fibers (*P400-025-SR*) to an *Ocean Insight* Ocean-FX spectrometer, sensitive from 200 to 850 nm, via an *Ocean Insight Square One* cuvette holder. Measurements were performed at ambient temperature using a *Hellma Analytics* quartz high precision cell (*108-F-10-40*) with a path length of 10 mm. Samples were prepared in acetophenone, filtered through a 0.2 μm PTFE syringe filter and measured at ambient temperature.

1.6 Attenuated Total -Reflectance Infrared Spectroscopy (ATR-IR)

All IR measurements were performed on a *Bruker* Alpha II ATR-IR from 400-4000 cm^{-1} at ambient temperature.

1.7 Thermogravimetric Analysis (TGA)

TGA measurements were carried out on the *TA* Instruments TGA 5500 under nitrogen atmosphere using platinum TGA sample pans and with a heating rate of 10 K min^{-1} over a temperature range from 25 to 600 °C.

1.8 Differential Scanning Calorimetry (DSC)

Thermal properties were measured on a *TA* DSC 2500 with a heating rate of 20 K·min^{-1} from -90 °C and 100 °C in *TA* Tzero sample holder. The glass transition temperature T_g was determined in the second heating run to eliminate possible interference from the polymer's thermal history.

1.9 Flash Column Chromatography

Flash column chromatography was performed on a CombiFlash Rf+ (*Teledyne ISCO*). Factions were collected based on a UV detector (254, 280 nm and detection of the full UV-Vis spectrum). *Interchim* Puriflash 15 and 30 µm Silica-HP columns were used for the separations. The raw products were – unless otherwise noted deposited – on Celite® 565 by dissolving, mixing and evaporation off the volatiles under reduced pressure. Subsequently, the Celite® was transferred into a Teledyne plunger pre-column filled to 1/3 with aspherical silica gel 35-60 µm.

1.10 Optical Microscopy

2D images were acquired by digital optical microscope DSX1000 (Olympus) with a 10×long working distance objective. The feature sizes of structures were analyzed with DSX software.

1.11 Light Emitting Diode (LED) Characterization

For the photoreactions a 365 nm LED by *Boston Photonics* (2850 mW, Product Number: VC2X2C45L9-365), a 375 nm LED by *Boston Photonics* (3300 mW, Product Number: VC2X2C45L9-375) and a 430 nm LED by *Lumixtar* (30 W, Product Number: WL-P30EP4545UV140-430) were employed.

LED emission spectra (**Figure S1**) and output energies were recorded using an *Opsytec Dr. Gröbel* Spectroradiometer SR600 at the same distance (30 mm) from the sensor as in the photoreactions from the center of the crimp vial. LEDs were cooled during measurement to minimize any thermal effects on the emission power or sensor performance.

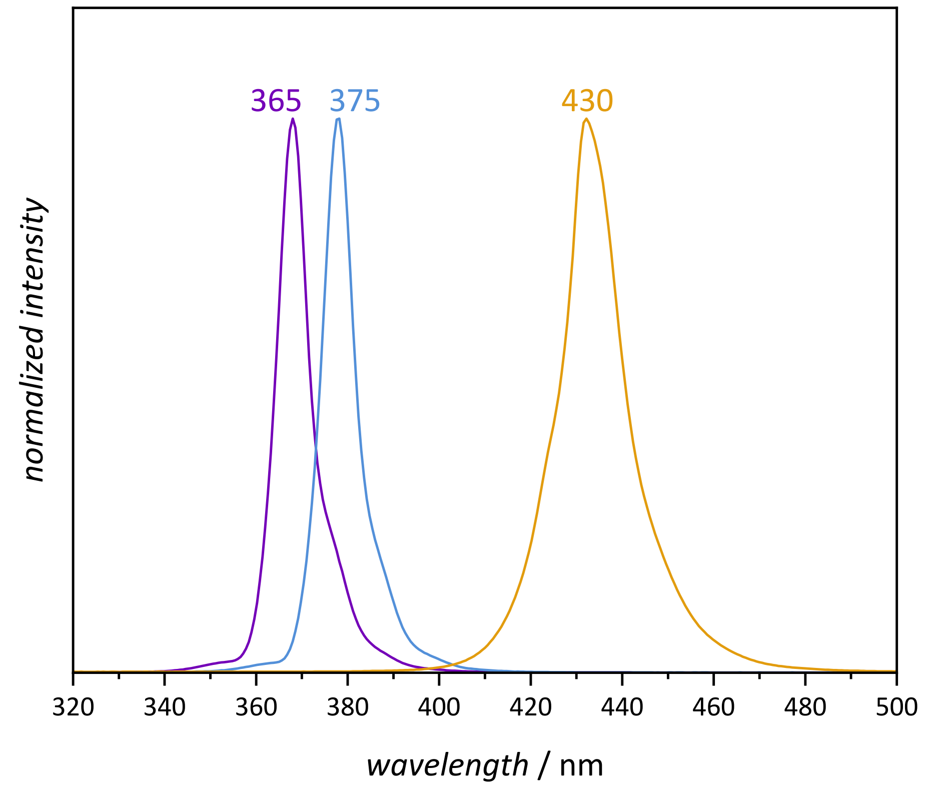

Figure S1. Emission spectra of LED light employed for irradiation, i.e., 365 nm (purple), 375 nm (blue) and 430 nm (yellow).

2 Synthesis

2.1 PolyDIO

2.1.1 5-Hydroxy-2,3-diphenyl-1*H*-inden-1-one (1)

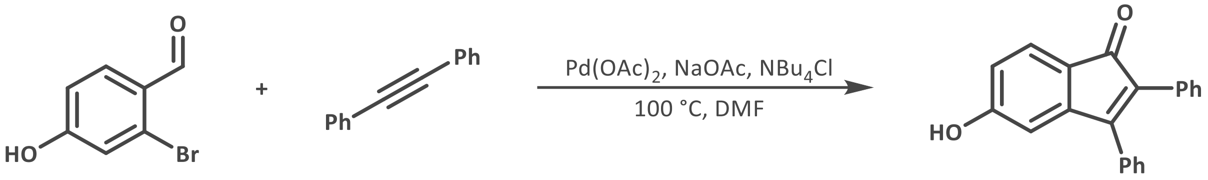

2-Bromo-4-hydroxy-benzaldehyde (4.00 g, 19.9 mmol, 1.00 eq.), diphenylacetylene (3.90 g, 21.9 mmol, 1.10 eq.), sodium acetate (NaOAc, 6.53 g, 79.6 mmol, 4.00 eq.), tetrabutylammonium chloride (NBu_4Cl, 5.53 g, 19.9 mmol, 1.00 eq.) and palladium(II) acetate ($Pd(OAc)_2$, 223 mg, 995 μmol, 0.05 eq.) were suspended in anhydrous dimethylformamide (DMF, 50 mL) and purged with nitrogen for 15 minutes. The reaction mixture was heated to 100 °C under continuous stirring for 48 h. Subsequently, water (100 mL) was added after cooling to ambient temperature in order to quench the reaction. The aqueous phase was extracted with ethyl acetate (EA, 2 × 200 mL), washed with saturated $NH_4Cl_{(aq.)}$ (200 mL), diluted $NaOH_{(aq.)}$ (3 × 200 mL) and diluted $HCl_{(aq.)}$ (200 mL), and dried over Na_2SO_4. After removal of the organic solvent, the crude product was purified via flash column chromatography (cyclohexane 70:30 EA, *v/v*). The product was obtained as a dark red crystalline solid (1.92 g, 32%).

^{1}H NMR (400 MHz, DMSO-*d$_6$*): δ (ppm) = 10.50 (s, 1H), 7.50 – 7.43 (m, 3H), 7.41 (d, *J* = 7.9 Hz, 1H), 7.38 – 7.33 (m, 2H), 7.31 – 7.23 (m, 3H), 7.21 – 7.14 (m, 2H), 6.64 (dd, *J* = 7.9, 2.0 Hz, 1H), 6.56 (d, *J* = 2.0 Hz, 1H).

^{13}C NMR (101 MHz, DMSO-*d$_6$*): δ (ppm) = 194.2, 163.2, 152.8, 147.8, 132.9, 132.2, 130.8, 129.7, 129.3, 128.9, 128.3, 128.0, 127.7, 125.2, 120.7, 113.5, 110.5.

2.1.2 5-((6-Hydroxyhexyl)oxy)-2,3-diphenyl-1*H*-inden-1-one (2)

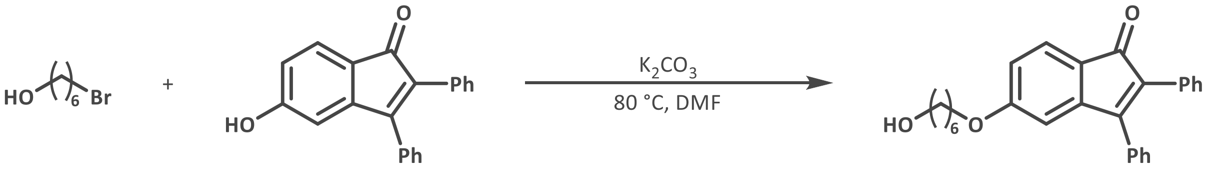

1 (1.92 g, 6.43 mmol, 1.00 eq.), 6-bromohexan-1-ol (2.33 g, 1.32 mL, 12.9 mmol, 2.00 eq.) and potassium carbonate (1.78 g, 12.9 mmol, 2.00 eq.) were dispersed in anhydrous dimethylformamide (DMF, 50 mL). The reaction mixture was stirred at 80 °C for 48 hours,

then ethyl acetate (EA, 150 mL) was added. The organic phase was washed with brine (3 × 200 mL) and water (200 mL), and the solvent removed under reduced pressure. The product was purified via flash column chromatography (cyclohexane 50:50 EA, *v/v*) and obtained as an orange solid (1.75 g, 68%).

^{1}H NMR (400 MHz, DMSO-d_6): δ (ppm) = 7.50 (d, *J* = 8.0 Hz, 1H), 7.45 (dd, *J* = 5.0, 1.9 Hz, 3H), 7.40 – 7.33 (m, 2H), 7.31 – 7.24 (m, 3H), 7.20 – 7.14 (m, 2H), 6.82 (dd, *J* = 8.1, 2.1 Hz, 1H), 6.59 (d, *J* = 2.1 Hz, 1H), 4.35 (t, *J* = 5.2 Hz, 1H), 4.01 (t, *J* = 6.4 Hz, 2H), 3.38 (td, *J* = 6.4, 5.1 Hz, 2H), 1.69 (p, *J* = 6.7 Hz, 2H), 1.46 – 1.24 (m, 6H).

^{13}C NMR (101 MHz, DMSO-d_6): δ (ppm) = 194.1, 163.7, 153.0, 147.2, 133.2, 131.9, 130.6, 129.7, 129.4, 128.9, 128.3, 128.0, 127.8, 124.9, 122.2, 111.6, 110.4, 68.2, 60.6, 32.4, 28.5, 25.3, 25.2.

2.1.3 3-((6-Hydroxyhexyl)oxy)-1a,6a-diphenyl-1a,6a-dihydro-6*H*-indeno[1,2-*b*]oxiren-6-one (DIO-OH)

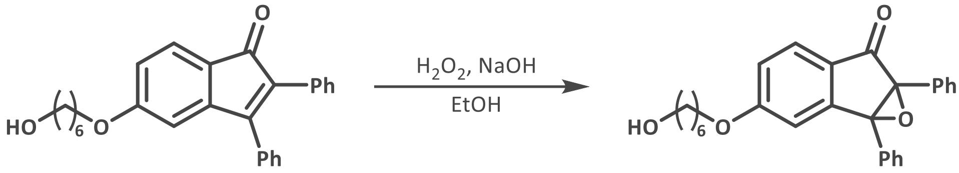

2 (1.75 g, 4.39 mmol, 1.00 eq.) was dissolved in a small amount of dichloromethane (DCM) and added to ethanol (200 mL) under stirring. Next, 30% $H_2O_{2(aq.)}$ (6.3 mL) was added, followed by dropwise addition of 4 M $NaOH_{(aq.)}$ 13.2 mL). The reaction mixture was stirred at ambient temperature for 1 hour. Over time, the orange solution turned colorless due to the oxidation and a white solid precipitated (NaOH). The solvent was removed under reduced pressure, and subsequently, water and ethyl acetate were added to the suspension until all solids were dissolved. The product was extracted with ethyl acetate (1 × 100 mL), washed with brine (2 × 200 mL), water (200 mL) and dried over Na_2SO_4. After removal of the solvent under reduced pressure, the product DIO-OH was obtained as a yellowish viscous liquid (1.67 g, 92%) and used without further purification.

^{1}H NMR (400 MHz, MeCN-d_3): δ (ppm) = 7.80 (d, *J* = 8.4 Hz, 1H), 7.49 – 7.40 (m, 2H), 7.37 – 7.25 (m, 8H), 7.05 (dd, *J* = 8.4, 2.3 Hz, 1H), 6.85 (d, *J* = 2.2 Hz, 1H), 4.01 (t, *J* = 6.6 Hz, 2H), 3.48 (td, *J* = 6.4, 5.2 Hz, 2H), 2.53 (t, *J* = 5.3 Hz, 1H), 1.73 (dt, *J* = 8.0, 6.5 Hz, 2H), 1.54 – 1.31 (m, 6H).

^{13}**C NMR** (101 MHz, MeCN-*d*$_3$): δ (ppm) = 195.7, 165.8, 152.7, 130.9, 129.8, 129.8, 129.5, 129.3, 129.0, 129.0, 128.8, 128.3, 128.1, 117.2, 112.3, 72.9, 72.9, 69.8, 62.5, 33.5, 29.6, 26.4, 26.3.

MS (*m/z*): calculated for $[C_{27}H_{16}O_4+H]^+$: 415.19, found 415.2.

2.1.4 3-((6-Hydroxyhexyl)oxy)-1a,6a-diphenyl-1a,6a-dihydro-6*H*-indeno[1,2-*b*]oxiren-6-one (3)

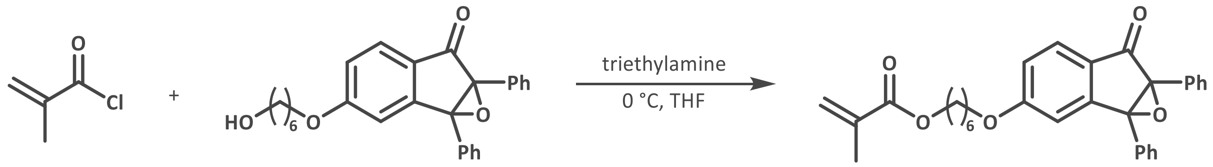

DIO-OH (809 mg, 1.95 mmol, 1.00 eq.) was dissolved in anhydrous tetrahydrofuran (THF, 20 mL) under nitrogen atmosphere and cooled to 0 °C with an ice bath. Triethylamine (988 mg, 1.36 mL, 9.76 mmol, 5.00 eq.) was added, followed by methacryloyl chloride (953 µL, 1.02 g, 9.76 mmol, 5.00 eq.) in a dropwise manner. The reaction mixture was stirred for 3 days. To quench the reaction, methanol (5 mL) was added and stirred for 5 minutes. The solvents were removed under reduced pressure and the crude product redissolved in ethyl acetate (EA) (100 mL). Subsequently, the organic phase was washed with saturated $NaHCO_{3(aq.)}$ (2 × 200 mL) and water (200 mL) and dried over Na_2SO_4. After removal of the organic solvent, the crude product was purified via flash column chromatography (cyclohexane 85:15 EA, *v/v*). The product was obtained as a yellowish viscous liquid (704 mg, 75%).

^{1}**H NMR** (400 MHz, MeCN-*d*$_3$): δ (ppm) = 7.80 (d, *J* = 8.4 Hz, 1H), 7.48 – 7.41 (m, 2H), 7.37 – 7.26 (m, 8H), 7.05 (dd, *J* = 8.5, 2.3 Hz, 1H), 6.85 (d, *J* = 2.2 Hz, 1H), 6.02 (dt, *J* = 1.8, 1.0 Hz, 1H), 5.57 (p, *J* = 1.6 Hz, 1H), 4.10 (t, *J* = 6.5 Hz, 2H), 4.03 (t, *J* = 6.5 Hz, 2H), 1.89 (t, *J* = 1.3 Hz, 3H), 1.80 – 1.71 (m, 2H), 1.70 – 1.61 (m, 2H), 1.52 – 1.35 (m, 4H).

^{13}**C NMR** (101 MHz, MeCN-*d*$_3$): δ (ppm) = 195.7, 168.0, 165.7, 152.7, 137.8, 131.0, 129.9, 129.8, 129.5, 129.3, 129.0, 129.0, 128.8, 128.3, 128.1, 125.6, 117.2, 112.3, 72.9, 79.7, 65.3, 29.5, 29.2, 26.3, 26.2, 26.2, 18.5.

MS (*m/z*): calculated for $[C_{31}H_{30}O_5+H]^+$: 483.22, found 483.3.

2.1.5 Poly(DIO-*co*-EHA) copolymer (polyDIO)

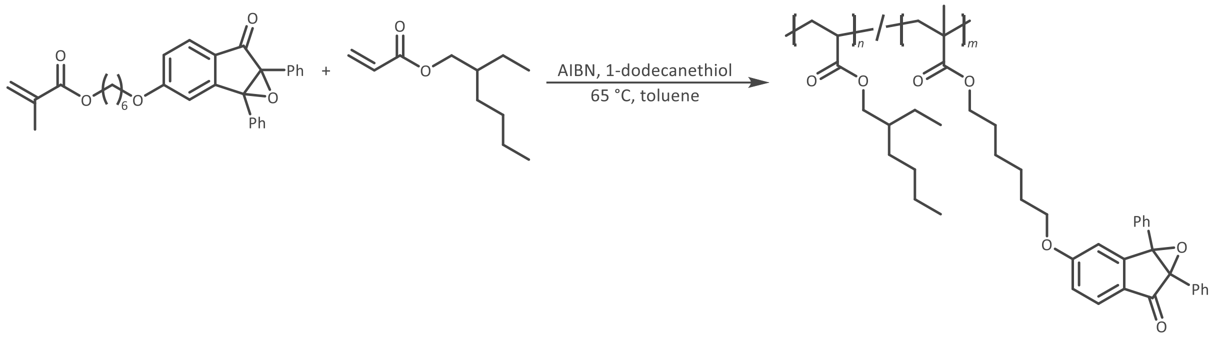

3 (1.61 g, 3.34 mmol, 1.00 eq.) and 2-ethylhexyl acrylate (EHA, 3.97 mL, 3.49 g, 18.9 mmol, 5.67 eq.) were prepared in a 25 mL round bottom flask under nitrogen atmosphere. Azobisisobutyronitrile (AIBN, 32.9 mg, 200 µmol, 0.06 eq.) and 1-dodecanethiol (54.1 mg, 64.0 µL, 267 µmol, 0.08 eq.) were dissolved in toluene (15 mL) and added to the comonomers. Next, the solution was bubbled with a slow stream of nitrogen flow for 15 minutes, sealed and stirred at 65 °C for 4 hours. The solvent was removed under reduced pressure, and the polymer was isolated by adding 20 mL methanol and stirring for 10 minutes at 40 °C and decanting the solvent. This washing process was repeated 5 times to remove leftover residual small molecules. Subsequently, the residual solvent was removed under reduced pressure and the product was obtained as a colorless, slightly turbid viscous liquid (4.54 g, 89%, M_n = 12 600 g mol^{-1}, $Đ$ = 1.72).

^{1}H NMR (400 MHz, $CDCl_3$): δ (ppm) = 7.83 (d, J = 5.9 Hz, 1H), 7.40 (d, J = 6.5 Hz, 2H), 7.33 – 7.21 (m, 8H), 6.96 (d, J = 8.3 Hz, 1H), 6.82 (s, 1H), 4.10 – 3.70 (m), 2.59 – 0.55 (m, aliphatic protons).

^{13}C NMR (101 MHz, $CDCl_3$): δ (ppm) = 194.9, 174.6, 164.7, 151.7, 130.1, 128.7, 128.6, 128.4, 128.4, 128.1, 128.1, 127.9, 127.8, 127.6, 115.7, 111.7, 72.0, 71.8, 68.7, 67.1, 64.8, 45.4, 41.7, 38.7, 35.6, 30.3, 29.8, 29.5, 29.0, 28.7, 25.8, 23.7, 23.1, 14.2, 10.9.

2.2 SA-3arm

2.2.1 6-(4-Bromo-3-nitrophenoxy)hexan-1-ol (4)

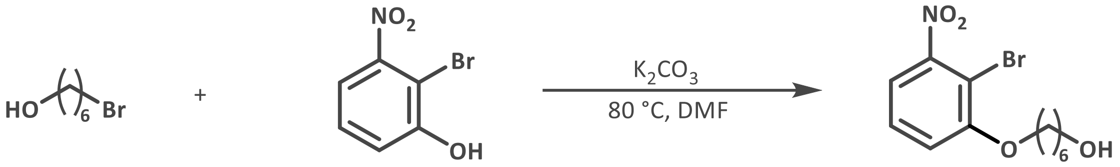

2-Bromo-3-nitro-phenol (9.83 g, 45.1 mmol, 1.00 eq.), 6-bromohexan-1-ol (8.85 mL, 12.2 g, 67.6 mmol, 1.50 eq.) and potassium carbonate (12.5 g, 90.2 mmol, 2.00 eq.) were dispersed in anhydrous dimethylformamide (DMF, 50 mL). The reaction mixture was stirred at 80 °C for 48 hours and then ethyl acetate (EA, 150 mL) was added. The organic phase was washed with brine (3 × 200 mL) and water (200 mL), and the solvent removed under reduced pressure. The product was obtained as a deep red-purple viscous liquid (12.7 g, 88%). The product was used in the next step without further purification.

^{1}H NMR (400 MHz, DMSO-d_6): δ (ppm) = 7.55 (t, J = 8.1 Hz, 1H), 7.49 (dd, J = 8.1, 1.5 Hz, 1H), 7.38 (dd, J = 8.2, 1.6 Hz, 1H), 4.35 (t, J = 5.2 Hz, 1H), 4.14 (t, J = 6.3 Hz, 2H), 3.39 (td, J = 6.4, 5.1 Hz, 2H), 1.75 (dt, J = 8.2, 6.4 Hz, 2H), 1.51 – 1.31 (m, 6H).

^{13}C NMR (101 MHz, DMSO-d_6): δ (ppm) = 156.1, 151.5, 129.7, 116.5, 116.0, 102.8, 69.6, 60.6, 32.5, 28.4, 25.3, 25.1.

2.2.2 6-(2-((2-Aminophenyl)thio)-3-nitrophenoxy)hexan-1-ol (5)

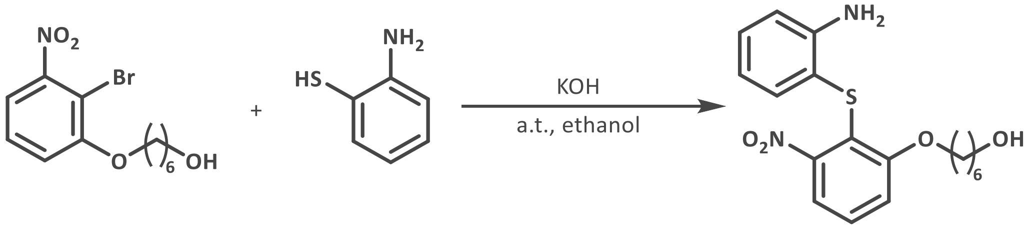

4 (12.7 g, 39.8 mmol, 1.00 eq.) was dissolved in ethanol (100 mL) and the solution was bubbled with nitrogen for 15 minutes. Next, potassium hydroxide (4.47 g, 79.6 mmol, 2.00 eq.) and 2-aminothiophenol (8.52 mL, 9.97 g, 79.6 mmol, 2.00 eq.) were added, and the reaction mixture was stirred for 20 hours at ambient temperature. The solvent was removed under reduced pressure, and the residuals redissolved in ethyl acetate (100 mL), washed with brine (2 × 300 mL) and water (300 mL) and dried over Na_2SO_4. After removal of the organic solvent, the crude product was purified via flash column chromatography (cyclohexane 40:60 EA, *v/v*). The

product was obtained as a viscous orange oil that solidified to an orange-red waxlike solid (12.5 g, 84%).

^{1}H NMR (400 MHz, DMSO-d_6): δ (ppm) = 7.50 (t, J = 8.2 Hz, 1H), 7.43 (dd, J = 8.1, 1.2 Hz, 1H), 7.28 (dd, J = 8.3, 1.3 Hz, 1H), 7.03 – 6.92 (m, 2H), 6.67 (dd, J = 8.1, 1.4 Hz, 1H), 6.45 (td, J = 7.5, 1.4 Hz, 1H), 5.23 (s, 2H), 4.34 (t, J = 5.1 Hz, 1H), 3.93 (t, J = 6.4 Hz, 2H), 3.37 (td, J = 6.5, 5.1 Hz, 2H), 1.57 – 1.45 (m, 2H), 1.44 – 1.33 (m, 2H), 1.32 – 1.16 (m, 4H).

^{13}C NMR (101 MHz, DMSO-d_6): δ (ppm) = 158.9, 154.1, 148.7, 133.3, 130.4, 129.2, 116.6, 115.7, 115.1, 114.9, 114.8, 114.7, 69.2, 60.7, 32.4, 28.2, 25.2.

2.2.3 4-Bromo-3-nitrobenzoate 6-(dibenzo[*b*,*f*][1,4,5]thiadiazepin-1-yloxy)hexan-1-ol (SA-OH)

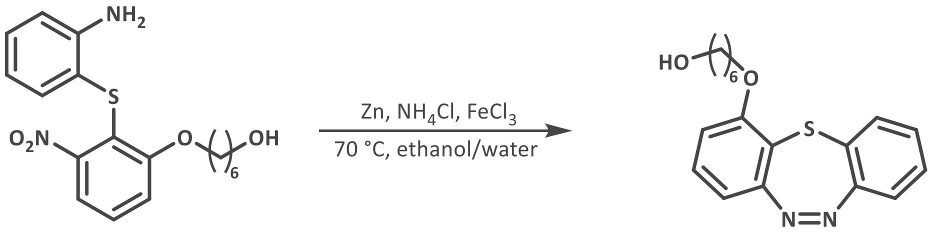

5 (2.50 g, 6.90 mmol, 1.00 eq.) was dissolved in ethanol (175 mL) and heated to 70 °C. Next, an aqueous 2 M ammonium chloride solution (31.6 mL) and zinc powder (1.71 g, 26.2 mmol, 3.80 eq.) were added, and stirred for 20 minutes at 70 °C. The reaction mixture was hot-filtered, and the filtrate directly poured into a solution of $FeCl_3 \cdot 6\ H_2O$ (3.73 g, 13.8 mmol, 2.00 eq.) in water (200 mL) and ice (200 g). While stirring for an additional 15 minutes, the color of the reaction mixture slowly turned black. Subsequently, acetic acid (100 mL) was added and stirring continued for 20 hours at ambient temperature. The acidic solution was neutralized with $NaHCO_3$ and NaOH, and then extracted with ethyl acetate (EA, 2 × 200 mL), followed by washing with water/brine (3 × 400 mL). The organic phase was dried over Na_2SO_4 and the solvent removed under reduced pressure. The product was purified via flash column chromatography (cyclohexane 50:50 EA, *v/v*) and obtained as a yellow-brownish viscous liquid (681 mg, 30%).

^{1}H NMR (400 MHz, MeCN-d_3): δ (ppm) = 7.55 (dd, J = 7.9, 1.6 Hz, 1H), 7.50 (td, J = 7.9, 7.5, 1.5 Hz, 1H), 7.40 (d, J = 8.1 Hz, 2H), 7.34 – 7.28 (m, 1H), 7.08 (dd, J = 8.0, 1.2 Hz, 1H), 6.95 (dd, J = 8.2, 1.2 Hz, 1H), 4.03 (t, J = 6.4 Hz, 2H), 3.51 (td, J = 6.5, 5.4 Hz, 2H), 1.79 (dq, J = 8.3, 6.4 Hz, 2H), 1.58 – 1.46 (m, 4H), 1.46 – 1.37 (m, 2H).

^{13}C NMR (101 MHz, MeCN-d_3): δ (ppm) = 157.7, 153.7, 153.3, 133.2, 132.1, 130.9, 130.7, 127.7, 120.7, 119.0, 113.6, 70.5, 62.5, 33.6, 29.7, 26.6, 26.3.

MS (*m/z*): calculated for $[C_{18}H_{20}N_2O_2S+H]^+$: 329.13, found 329.2.

2.2.4 4-bromo-3-nitrobenzoate 6-(dibenzo[*b,f*][1,4,5]thiadiazepin-1-yloxy)hexyl acrylate (6)

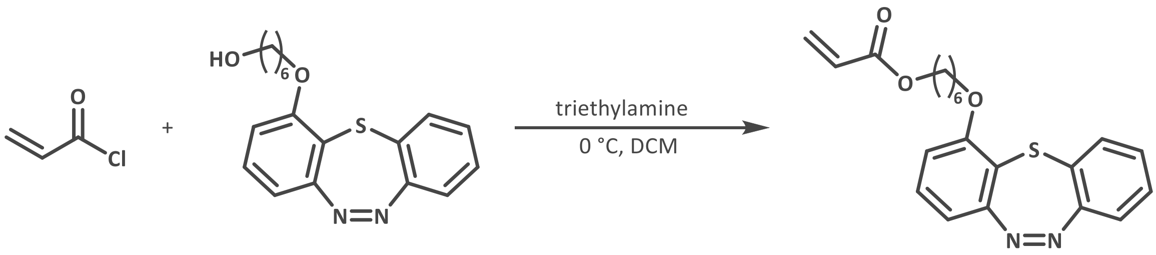

SA-OH (814 mg, 2.48 mmol, 1.00 eq.) was dissolved in DCM (70 mL) under nitrogen atmosphere and cooled to 0 °C with an ice bath. Triethylamine (NEt_3, 1.04 mL, 752 mg, 7.44 mmol, 3.00 eq.) was added and lastly acryloyl chloride (604 µL, 673 mg, 7.44 mmol, 3.00 eq.) in a dropwise manner. The reaction mixture was stirred for 24 hours and washed with saturated $NaHCO_{3(aq.)}$ (2 × 100 mL) and water (100 mL) and dried over Na_2SO_4. After removal of the organic solvent, the crude product was purified via flash column chromatography (cyclohexane 70:30 EA, *v/v*). The product was obtained as a yellow-orange viscous liquid (402 mg, 42%).

^{1}H NMR (400 MHz, $CDCl_3$): δ (ppm) = 7.58 (dd, *J* = 7.9, 1.4 Hz, 1H), 7.43 (td, *J* = 7.6, 1.4 Hz, 1H), 7.36 – 7.29 (m, 2H), 7.27 – 7.21 (m, 1H), 7.14 (dd, *J* = 7.9, 1.2 Hz, 1H), 6.81 (dd, *J* = 8.2, 1.2 Hz, 1H), 6.40 (dd, *J* = 17.3, 1.5 Hz, 1H), 6.13 (dd, *J* = 17.3, 10.4 Hz, 1H), 5.82 (dd, *J* = 10.4, 1.5 Hz, 1H), 4.19 (t, *J* = 6.7 Hz, 2H), 4.00 (t, *J* = 6.3 Hz, 2H), 1.90 – 1.81 (m, 2H), 1.74 (p, *J* = 6.9 Hz, 2H), 1.64 – 1.43 (m, 4H).

^{13}C NMR (101 MHz, $CDCl_3$): δ (ppm) = 166.5, 156.8, 153.1, 152.6, 132.4, 131.7, 130.7, 129.6, 129.4, 129.3, 128.7, 127.5, 120.6, 119.1, 112.3, 69.5, 64.6, 29.1, 28.8, 25.9, 25.8.

MS (*m/z*): calculated for $[C_{21}H_{22}N_2O_3S+H]^+$: 383.14, found 383.2.

2.2.5 SA-3arm

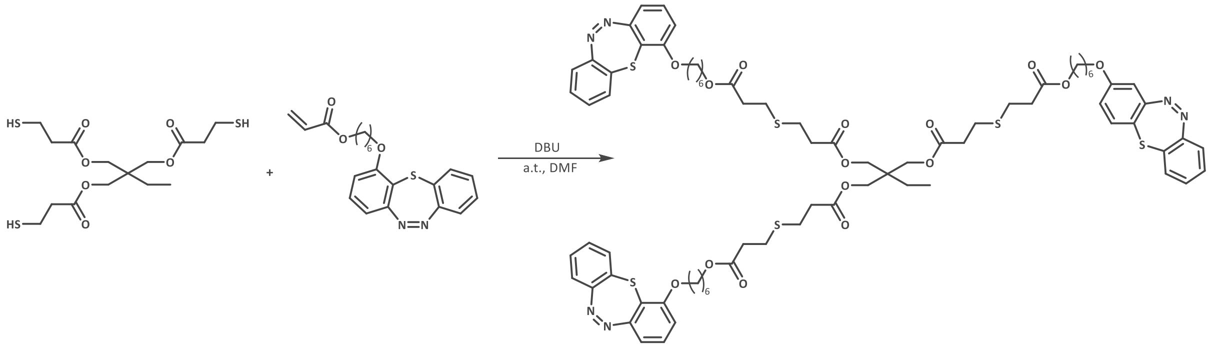

The acrylated photoswitch **6** (708 mg, 1.85 mmol, 1.00 eq.) and trimethylolpropane tris(3-mercaptopropionate) (152 µL, 184 mg, 463 µmol, 0.25 eq.) were dissolved in dimethylformamide (DMF, 10 mL). 1,8-Diazabicyclo [5.4.0]undec-7-ene (DBU, 13.8 µL, 14.1 mg, 92.6 µmol, 0.05 eq.) was added as a catalyst and the reaction mixture was stirred for 30 minutes. Then, ethyl acetate (EA, 100 mL) was added, and the organic phase was washed with brine (2 × 200 mL), then water (200 mL) and dried over Na_2SO_4. The solvent was removed under reduced pressure and the product was purified via flash column chromatography (cyclohexane 30:70 EA, *v/v*) and obtained as a very viscous, yellow liquid (568 mg, 79%). The excess starting material **6** was recovered from the flash column chromatography as well.

^{1}H NMR (400 MHz, $CDCl_3$): δ (ppm) = 7.57 (dd, *J* = 7.9, 1.4 Hz, 3H), 7.42 (td, *J* = 7.6, 1.4 Hz, 3H), 7.36 – 7.29 (m, 6H), 7.26 – 7.21 (m, 3H), 7.13 (dd, *J* = 8.0, 1.2 Hz, 3H), 6.81 (dd, *J* = 8.2, 1.2 Hz, 3H), 4.12 (t, *J* = 6.7 Hz, 6H), 4.04 (s, 6H), 4.00 (t, *J* = 6.3 Hz, 6H), 2.82 – 2.76 (m, 12H), 2.61 (q, *J* = 6.8 Hz, 12H), 1.90 – 1.79 (m, 6H), 1.70 (p, *J* = 6.9 Hz, 6H), 1.59 – 1.52 (m, 6H), 1.50 – 1.41 (m, 8H), 0.88 (t, *J* = 7.5 Hz, 3H).

^{13}C NMR (101 MHz, $CDCl_3$): δ (ppm) = 172.0, 171.6, 156.7, 153.1, 152.6, 132.4, 131.6, 129.6, 129.4, 129.3, 127.5, 120.5, 119.1, 112.3, 69.5, 64.9, 64.0, 40.9, 34.9, 34.7, 29.1, 28.7, 27.2, 27.1, 25.9, 25.8, 23.1, 7.5.

3 Two-color Photoreactions Using LED Lamps

3.1 Reaction setup

The reaction setup and sample preparation for the two-color test reactions were identical to the set-up we employed for our previous study on the synergistic two-color photochemistry of DIO and SA.[3]

Stock solutions of the DIO/SA combinations with 1,3,5-trimethoxybenzene (TMB) as an internal standard were prepared in 2 mL crimp vials by *LabCo Scientific* (Clear Glass Vial, 12x32 mm, Flat Bottom, Product Number: 355.100.100) under nitrogen atmosphere. The vial was crimped with a magnetic cap to facilitate attachment to the custom-made LED holder shown in **Figure S2** and irradiated with 365 nm (1.69 mW cm^{-2}, 3.10×10^{15} photons s^{-1} cm^{-2}) and 430 nm (27.9 mW cm^{-2}, 6.04×10^{16} photons s^{-1} cm^{-2}) while stirring at 500 rpm. Both heat sinks were fixed on opposing sides with a distance of 35 mm to the center of the reaction vial. Different irradiances at 365 nm were realized by pulsing the LED on the millisecond timescale (i.e., a 10 ms LED pulse and varying times between pulses), thus achieving precise control over low irradiances. All reactions were performed under one-color irradiation with 365 nm only and under two-color irradiation with 365 + 430 nm for comparison of the synergistic efficiencies.

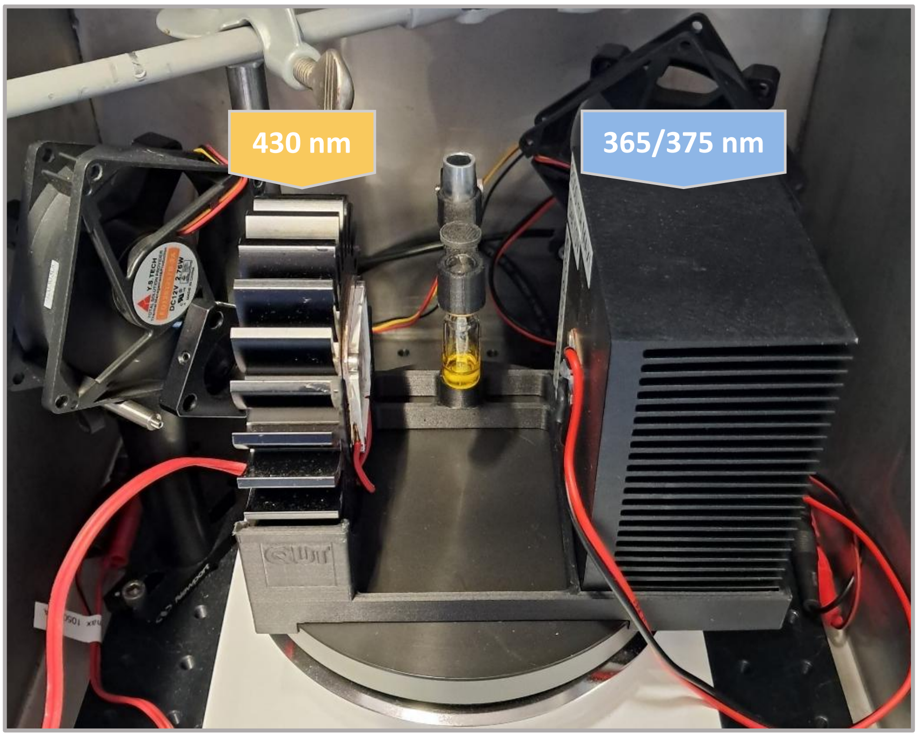

Figure S2. Representative setup for the photoreactions consisting of a magnetic stirrer, two LED light sources (365/375 and 430 nm) in a custom-made LED holder and fans for cooling.

After the photoreactions, 50 µL of the sample were diluted with 950 µL acetonitrile and passed through a syringe filter (0.2 µm, PTFE) into an LC-MS vial. In the LC traces for an absorbance at 235 nm, the peak integrals of TMB, SA and DIOSA were calculated. The yield Y of a reaction was determined according to the following formula

$$Y = 1 - \frac{I_{SA}}{I_{SA,0}}$$

where $I_{SA,0}$ is the integral of the SA compound at the beginning of the reaction, normalized by dividing by the integral of the internal standard TMB. I_{SA} is the normalized integral of the SA compound at the end of the reaction. Due to the fact that side reactions occur to DIO under irradiation,[3] tracking the DIO conversion to calculate the reaction yield is not applicable.

The synergistic ratio ϕ_{syn} was calculated via the following formula

$$\phi_{syn} = \frac{Y_2}{Y_1}$$

where Y_1 is the reaction yield of a one-color reaction, and Y_2 is the yield of the corresponding two-color reaction.

3.2 Small molecule test reactions

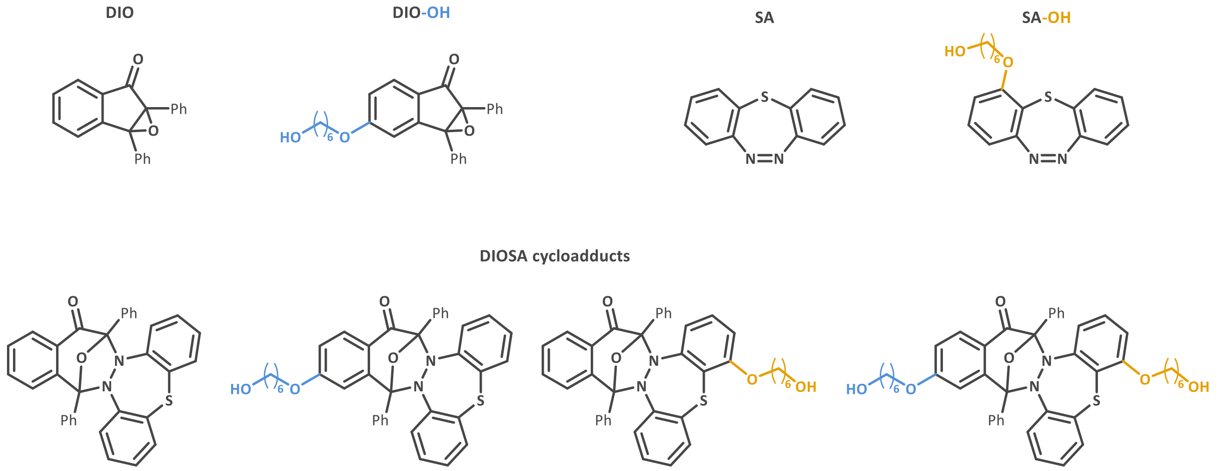

Figure S3. Schematic representation of different combinations of DIO- and SA-derivatives and their DIOSA cycloadducts that were investigated herein.

For the small-molecule test reactions, a 5 mM stock solution of DIO/DIO-OH, SA/SA-OH and TMB in HPLC grade acetonitrile (2 mL) was prepared and purged by bubbling with nitrogen.

Different stock solutions were prepared for the four different photoswitch combinations DIO/SA (as a reference), DIO-OH/SA DIO/SA-OH, DIO-OH/SA-OH as implied in **Figure S3**. For each sample, a stir bar and 250 µL of the stock solution, containing DIO (373 µg, 1.25 nmol, 1.00 eq.) or DIO-OH (518 µg, 1.25 nmol, 1.00 eq.), SA (265 µg,1.25 nmol, 1.00 eq.) or SA-OH (411 µg,1.25 nmol, 1.00 eq.) and TMB (105 µg, 0.625 nmol, 0.50 eq.), were added into a 2 mL crimp vial and crimped with a magnetic cap to facilitate attachment to the custom-made LED holder. For every combination of photoswitches a one- and a two-color reaction were performed, and the samples were irradiated at 365 nm (1.69 mW cm^{-2}, 3.10×10^{15} photons s^{-1} cm^{-2}) and 430 nm (27.9 mW cm^{-2}, 6.04×10^{16} photons s^{-1} cm^{-2}) for 10 minutes.

A comparison of the product yields for the one- and two-color reactions of different combinations of DIO/DIO-OH and SA/SA-OH in **Figure S4** shows a synergistic effect for all combinations and ϕ_{syn} for these reactions is shown in **Figure S5**. The successful formation of the diverse cycloadducts was proven via LC-MS of the crude reaction mixtures. The *m/z* for all protonated DIOSA derivatives ($[M+H]^+$), was found as shown in **Figure S6** to **Figure S9**.

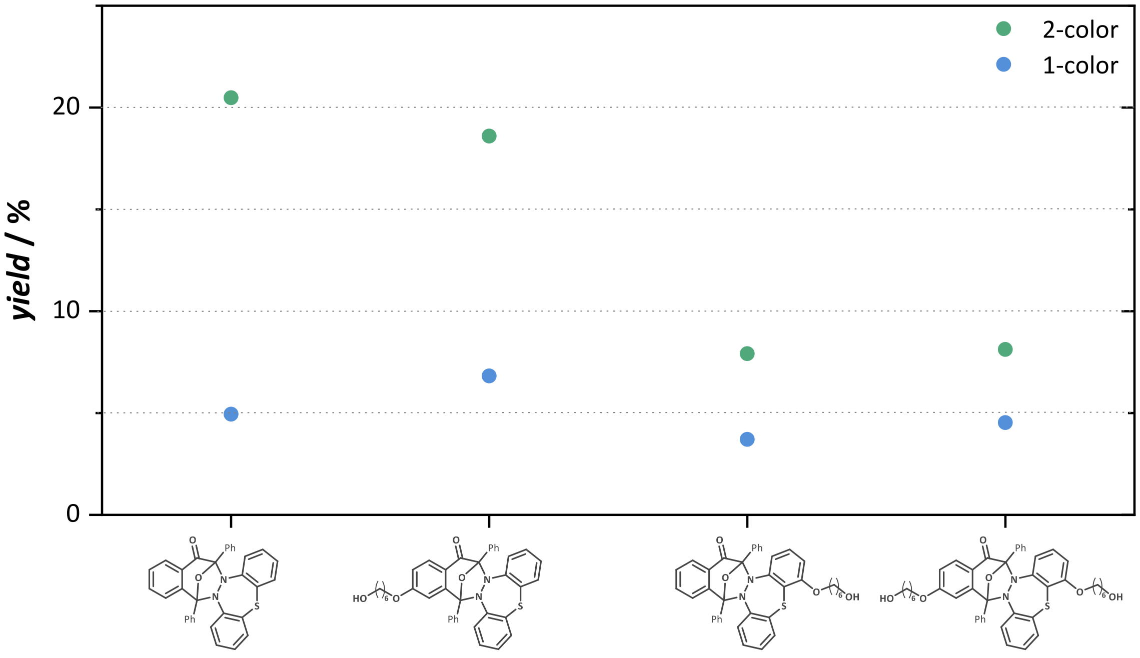

Figure S4. Comparison of the product yields for the one- and two-color reactions of different combinations of DIO/DIO-OH and SA/SA-OH.

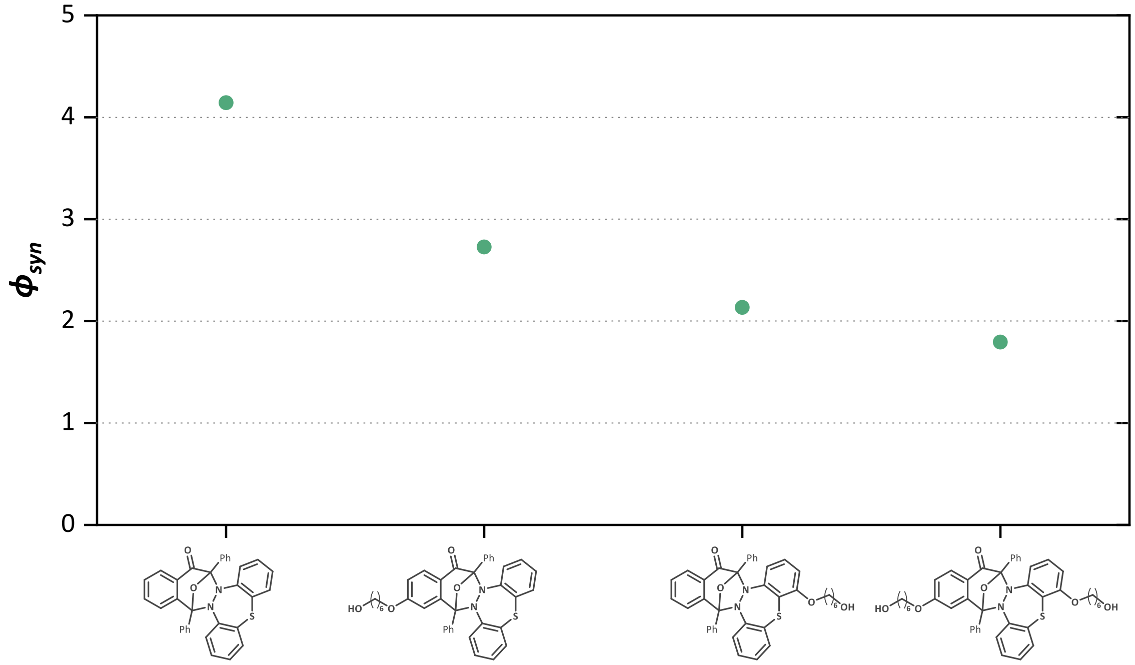

Figure S5. Comparison of the synergistic efficiencies (ϕ_{syn}) of the two-color reactions of different combinations of DIO/DIO-OH and SA/SA-OH.

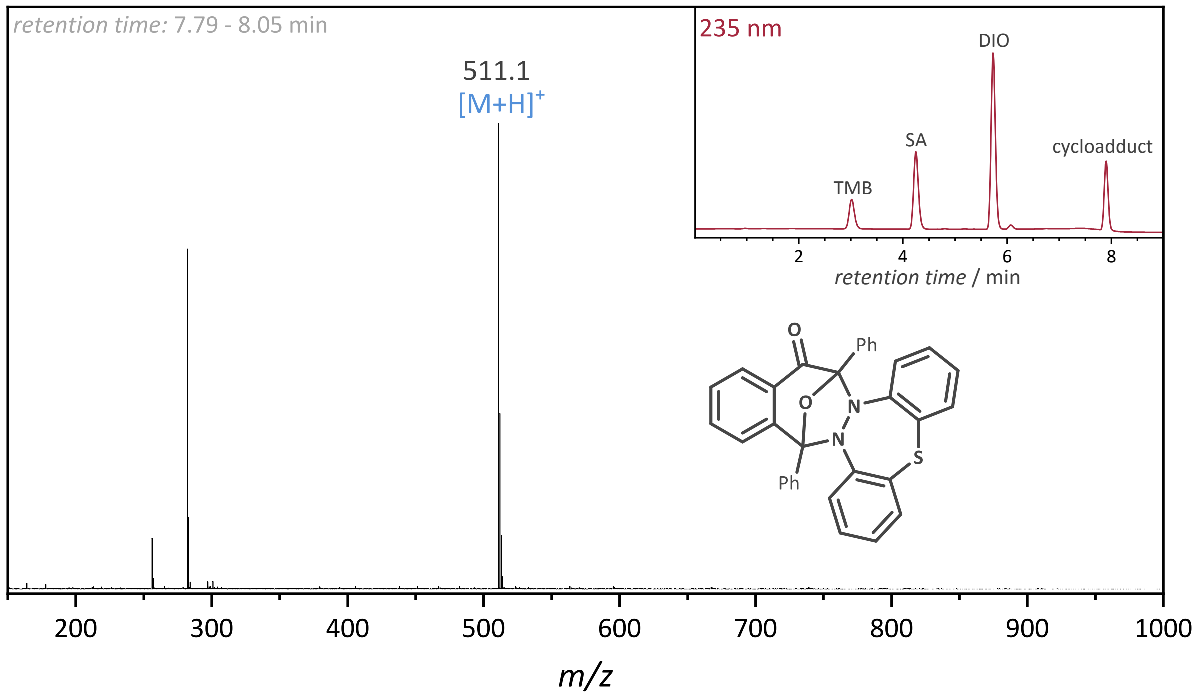

Figure S6. LC-trace (235 nm detector wavelength) of the crude mixture of the two-color photoreaction between DIO and SA and accumulated mass-spectra of the formed DIOSA derivative (cycloadduct).

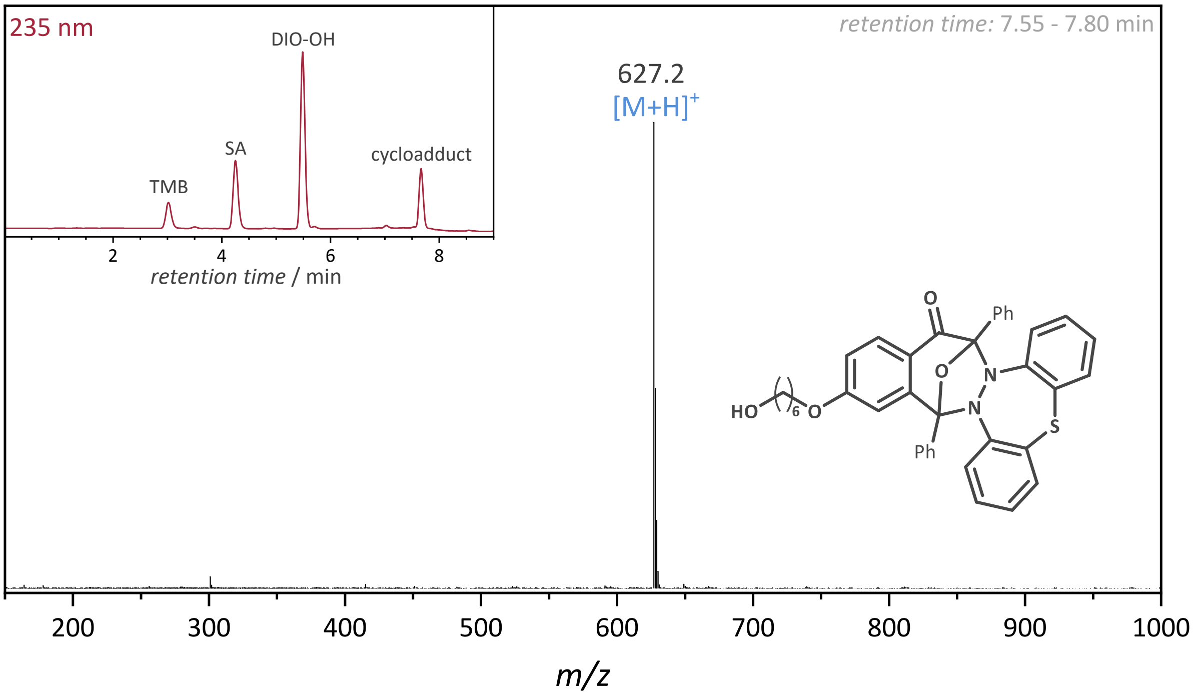

Figure S7. LC-trace (235 nm detector wavelength) of the crude mixture of the two-color photoreaction between DIO-OH and SA and accumulated mass-spectra of the formed DIOSA derivative (cycloadduct).

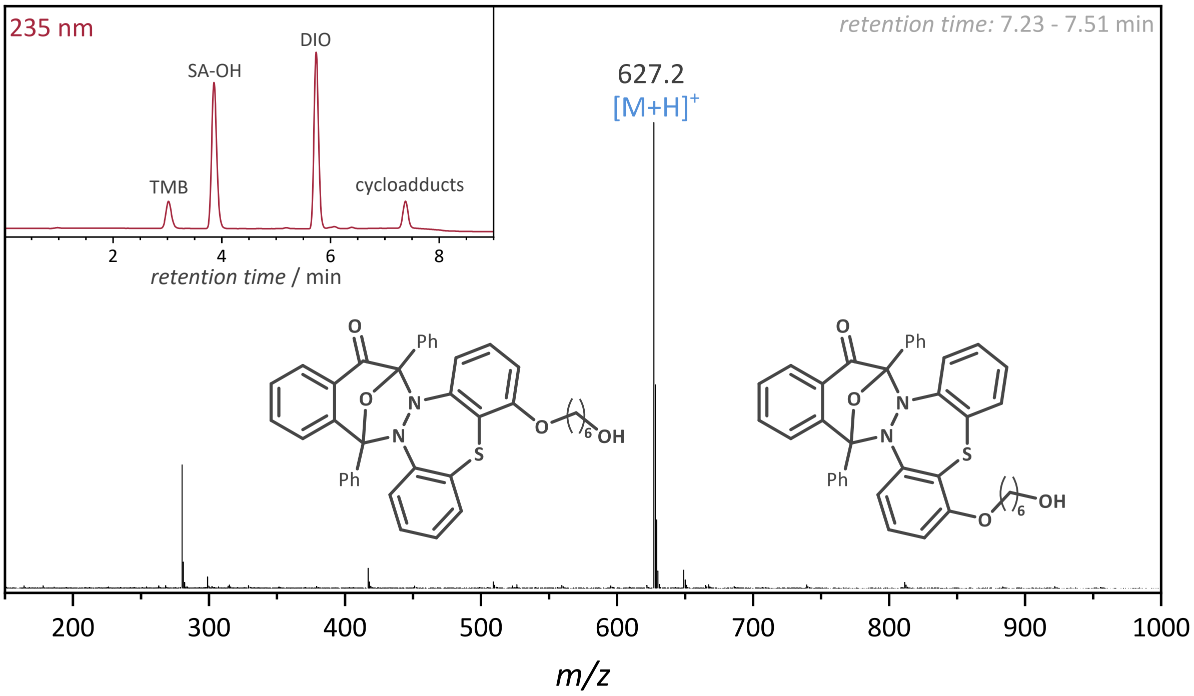

Figure S8. LC-trace (235 nm detector wavelength) of the crude mixture of the two-color photoreaction between DIO and SA-OH and accumulated mass-spectra of the formed DIOSA derivatives (cycloadduct).

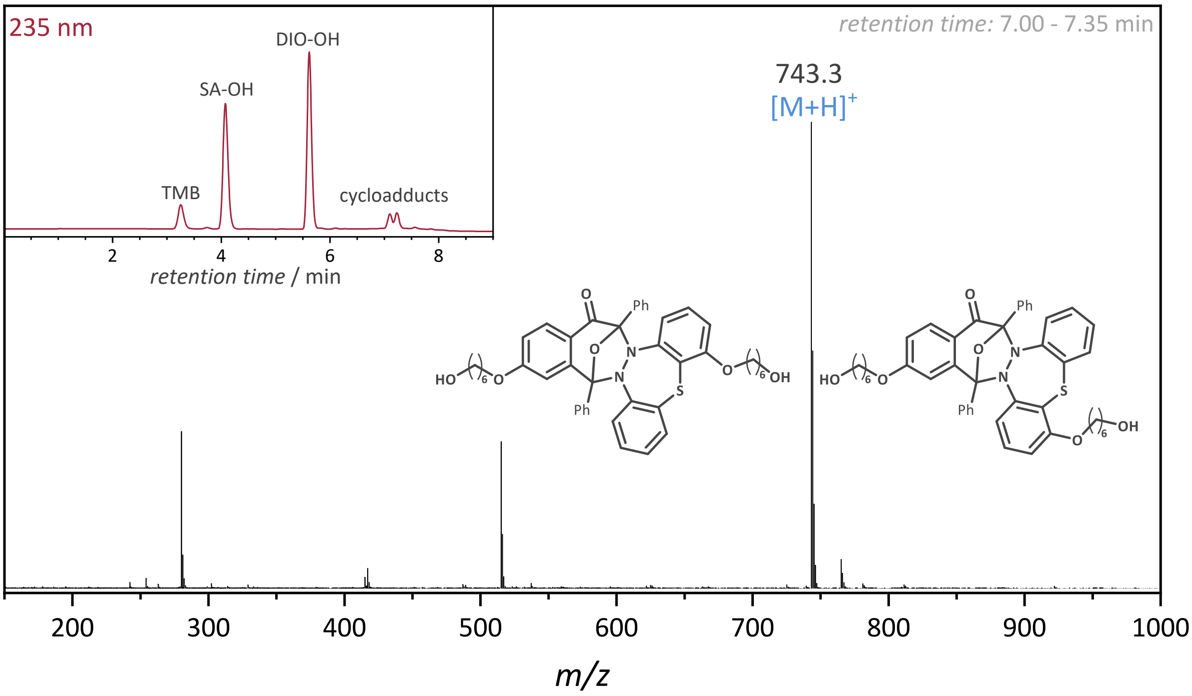

Figure S9. LC-trace (235 nm detector wavelength) of the crude mixture of the two-color photoreaction between DIO-OH and SA-OH and accumulated mass-spectra of the formed DIOSA derivatives (cycloadducts). Two product signals are present due to the formation of regioisomers.

3.3 Testing acetophenone as solvent

For testing acetophenone as a solvent for the synergistic reaction, the same reaction setup and conditions as for the reactions in **SI Section 3.2** were chosen. A 5 mM stock solution of DIO, SA and TMB in acetophenone (2 mL) was prepared and purged by bubbling with nitrogen. For each sample, a stirring bar and 250 µL of the stock solution, containing DIO (373 µg, 1.25 nmol, 1.00 eq.), SA (265 µg,1.25 nmol, 1.00 eq.) and TMB (105 µg, 0.625 nmol, 0.50 eq.), were added into a 2 mL crimp vial and crimped with a magnetic cap to facilitate attachment to the custom-made LED holder. For the one- and the two-color reaction, the samples were irradiated at 365 nm (1.69 mW cm^{-2}, 3.10×10^{15} photons s^{-1} cm^{-2}) and 430 nm (27.9 mW cm^{-2}, 6.04×10^{16} photons s^{-1} cm^{-2}) for 10 minutes.

Calculation of the product yields according to the calculations in **SI Section 3.1** were obtained as 2.4% and 18.0% for the one- and two-color reactions, respectively. The synergistic efficiency was calculated to be 7.5, which is higher than the same reaction in acetonitrile (4.1, compare **Figure S5**). The synergistic acceleration of DIOSA formation becomes apparent by comparing the product integrals in the LC traces of the stock solutions after irradiation (**Figure S10**).

Consequently, acetophenone was proven to be a reliable solvent for preparing photoresist formulations.

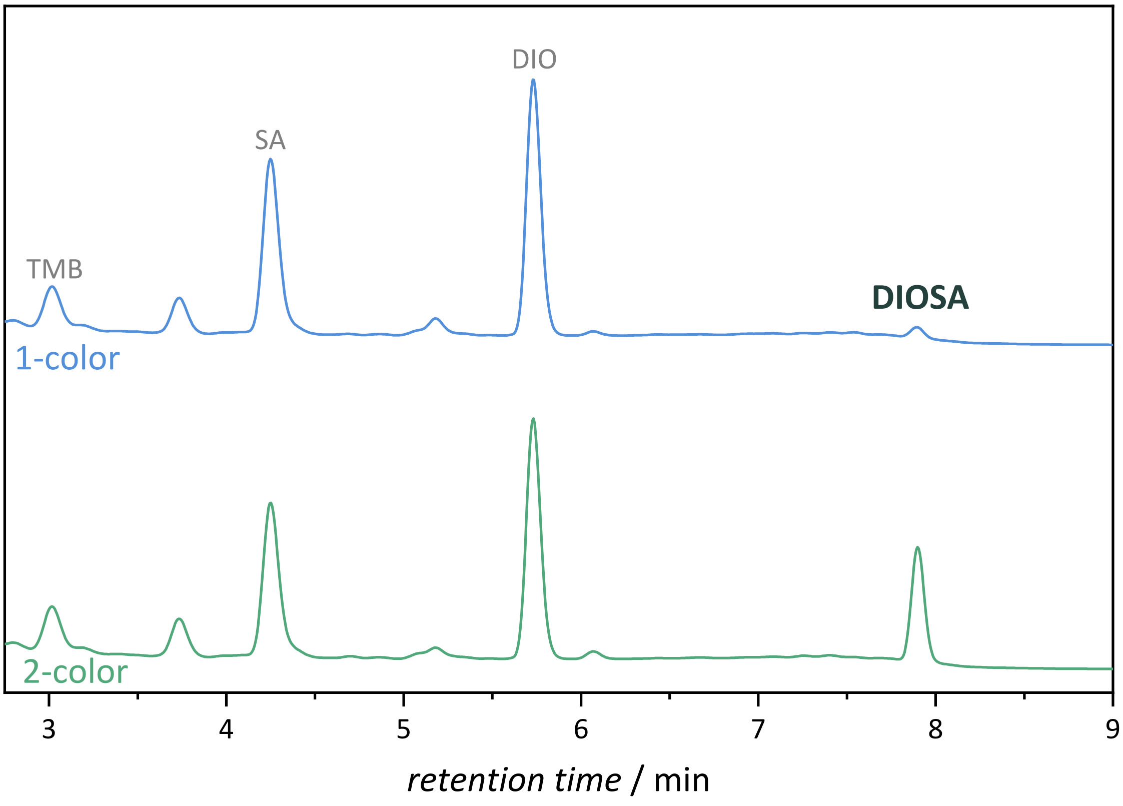

Figure S10. UV-detector LC traces at 235 nm of the reaction of DIO and SA to DIOSA in acetophenone under one- and two-color irradiation.

3.4 SEC-tracked crosslinking

For the SEC tracked crosslinking of polyDIO (184 mg, 100 µmol, 1.00 eq) and SA-3arm (51.1 mg, 100 µmol, 1.00 eq.), a stock solution in 2.1 mL acetophenone (90 wt%, 47.5 mM) was prepared and purged by bubbling with nitrogen (amount of substance, equivalents and concentration are given in regard to the photoswitching moieties). For each sample, a stir bar and 400 µL of the stock solution were added into a 2 mL crimp vial by *LabCo Scientific* (Clear Glass Vial, 12x32 mm, Flat Bottom, Product Number: 355.100.100) under nitrogen atmosphere. The vial was crimped with a magnetic cap and irradiated in the same reaction setup that was used for the small-molecule test reactions (**SI Section 3.2**). Photon fluxes of 1.55×10^{16} photons s^{-1} cm^{-2} for 365 and 375 nm and 6.04×10^{16} photons s^{-1} cm^{-2} for 430 nm were employed. Samples were taken every 30 minutes for 180 minutes and measured via SEC to investigate the qualitative differences in the increase of M_n during irradiation between one- (365/375 nm) and two-color (365/375 + 430 nm) reactions (**Figure S11** and **Figure S12**).

Additionally, 430 nm one-color irradiation was performed for 240 minutes without any significant changes as shown in **Figure S13**, showing the full orthogonality of this wavelength.

It should be noted that SEC gives only relative values regarding the molar masses of polymers, as the separation in the column depends on hydrodynamic volume rather than providing absolute molar masses and is calibrated against narrow-disperse PMMA standards. Additionally, only the soluble fraction is captured in the analysis. With increasing crosslink density, larger, insoluble network structures are retained by the syringe filter in the SEC sample preparation. This effect is supported by the increasing pressure required to pass the dissolved reaction mixture through the PTFE filter for longer reaction times during sample preparation. Despite these limitations, SEC remains a practical and effective tool for monitoring the relative extent of crosslinking, as it is easy to perform and provides clear insight into molar mass development during network formation[4] and therefore allows for comparison of the synergistic efficiency under different reaction conditions.

The DRI signal of the SEC elution traces, as a qualitative measure of the concentrations, were normalized to the SA-3arm signal to facilitate comparability (**Figure S11** and **S12**). The small molecule SA-3arm is consumed over time by the crosslinking reaction and its concentration decreases, while the concentration of the network in the polymer region continuously increases unless it is filtered off. Because this relative decrease of the concentration of SA-3arm that appears constant due to normalization, the concentration of the polymer traces seems to increase significantly over time. This effect is partly explained by the incorporation of SA-3arm into the network but mainly to the decrease of the DRI of the normalization standard.

In addition, two opposing effects have to be considered regarding the shifts to lower elution volumes over time. On the one hand, the molar mass of the polymer steadily increases with every new crosslinking reaction. On the other hand, a covalently crosslinked polymer network is much more compact with a lower hydrodynamic volume compared to a linear polymer chain of the same molar mass. Therefore, the apparent molar mass gained by comparison to linear polymer standards will drastically underestimate the true molar mass of the network, which will become more and more prominent for a growing network.

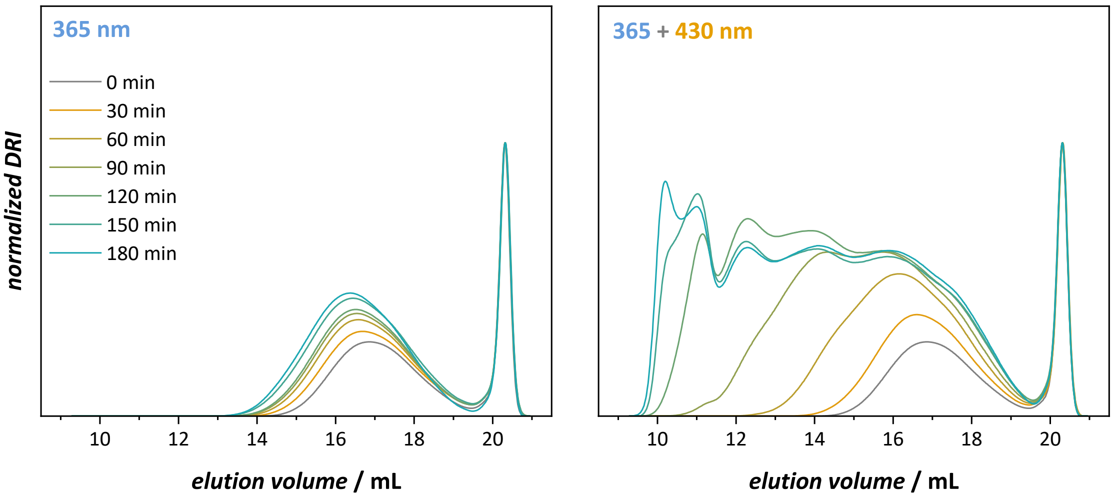

Figure S11. Evolution of SEC traces for the photoresist composed of polyDIO and SA-3arm in acetophenone under one-color (365 nm, left) and two-color (365 + 430 nm, right) irradiation over 180 minutes. Samples were taken every 30 minutes to monitor the progression of the crosslinking reaction.

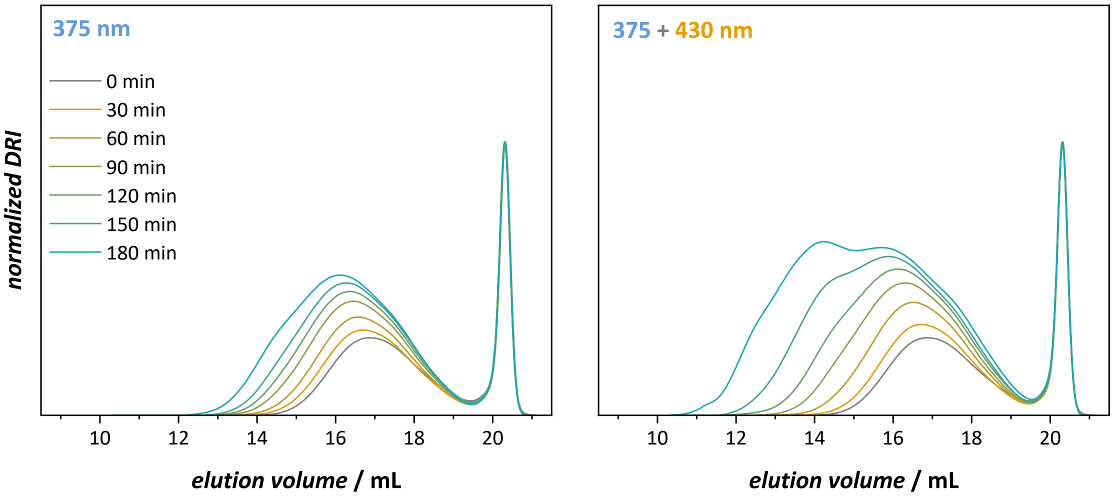

Figure S12. Evolution of SEC traces for the photoresist composed of polyDIO and SA-3arm in acetophenone under one-color (375 nm, left) and two-color (375 + 430 nm, right) irradiation over 180 minutes. Samples were taken every 30 minutes to monitor the progression of the crosslinking reaction.

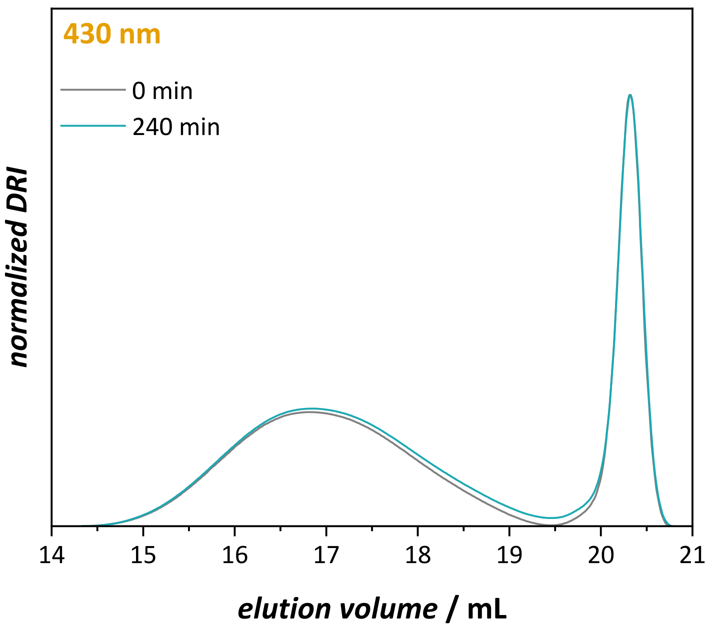

Figure S13. SEC traces of the photoresist composed of polyDIO and SA-3arm in acetophenone before (grey) and after 240 minutes of one-color irradiation at 430 nm (turquoise), showing no significant shift.

4 NMR Spectra of the Photoresists

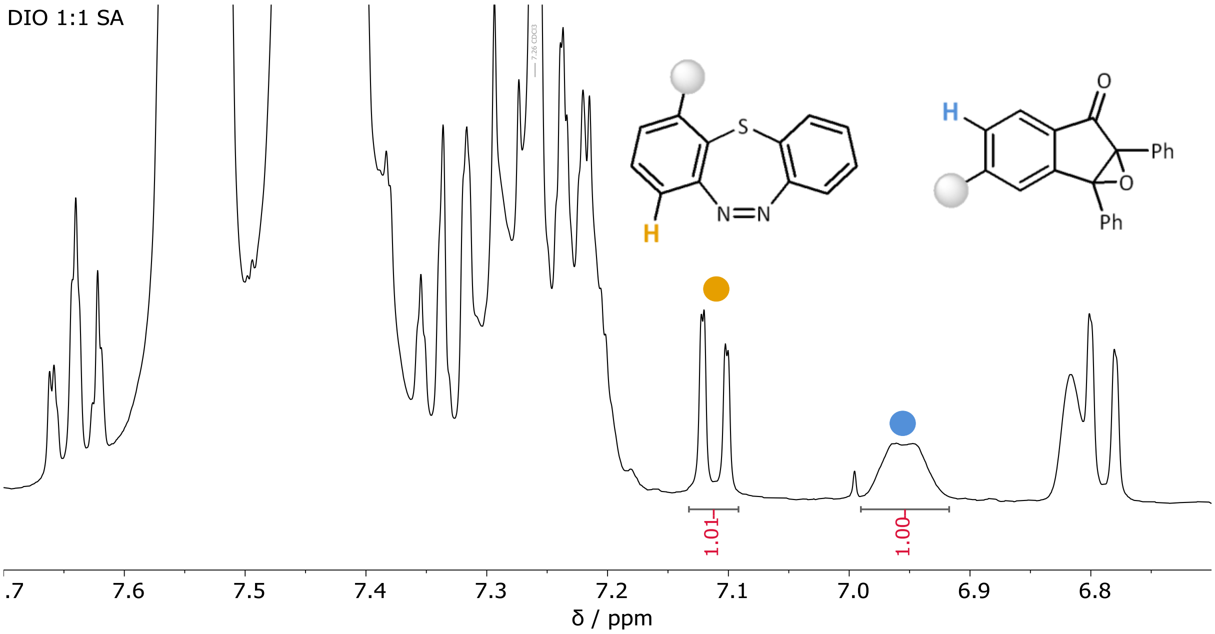

Figure S14. ^{1}H NMR (400 MHz) spectrum in $CDCl_3$ at ambient temperature of the photoresist formulation in acetophenone for the two-color SEC crosslinking tests, with a DIO:SA ratio of 1:1 ([DIO] = 47.5 mM, [SA] = 47.5 mM).

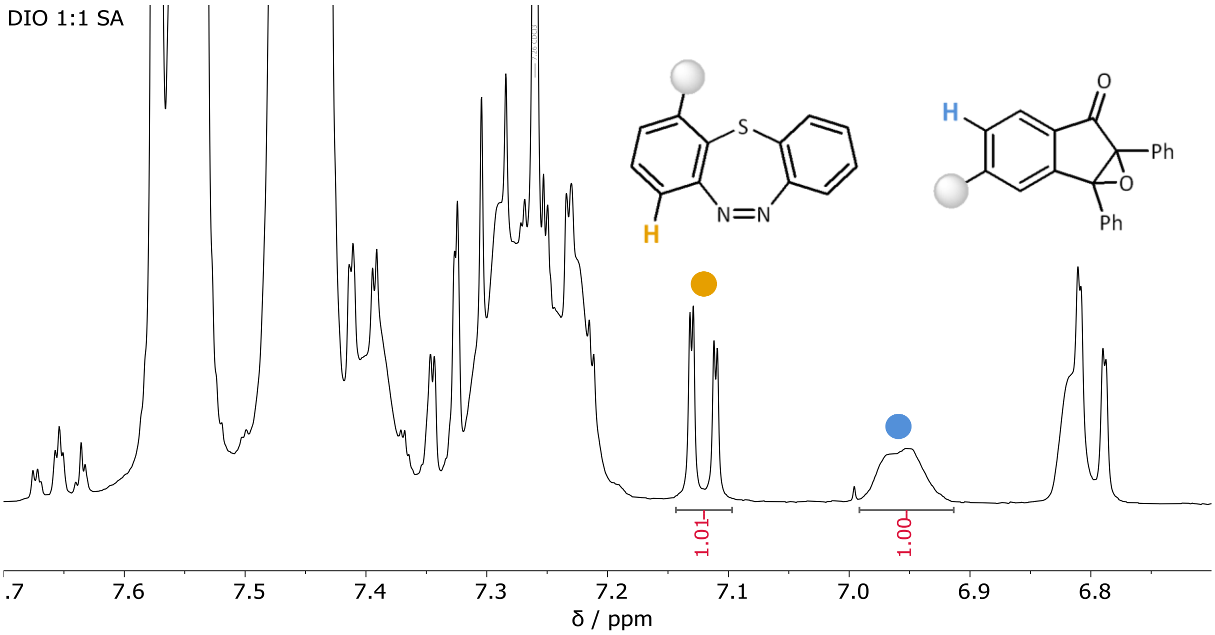

Figure S15. ^{1}H NMR (400 MHz) spectrum in $CDCl_3$ at ambient temperature of the photoresist formulation in acetophenone for two-color lithography with a DIO:SA ratio of 1:1 ([DIO] = 70.5 mM, [SA] = 70.5 mM).

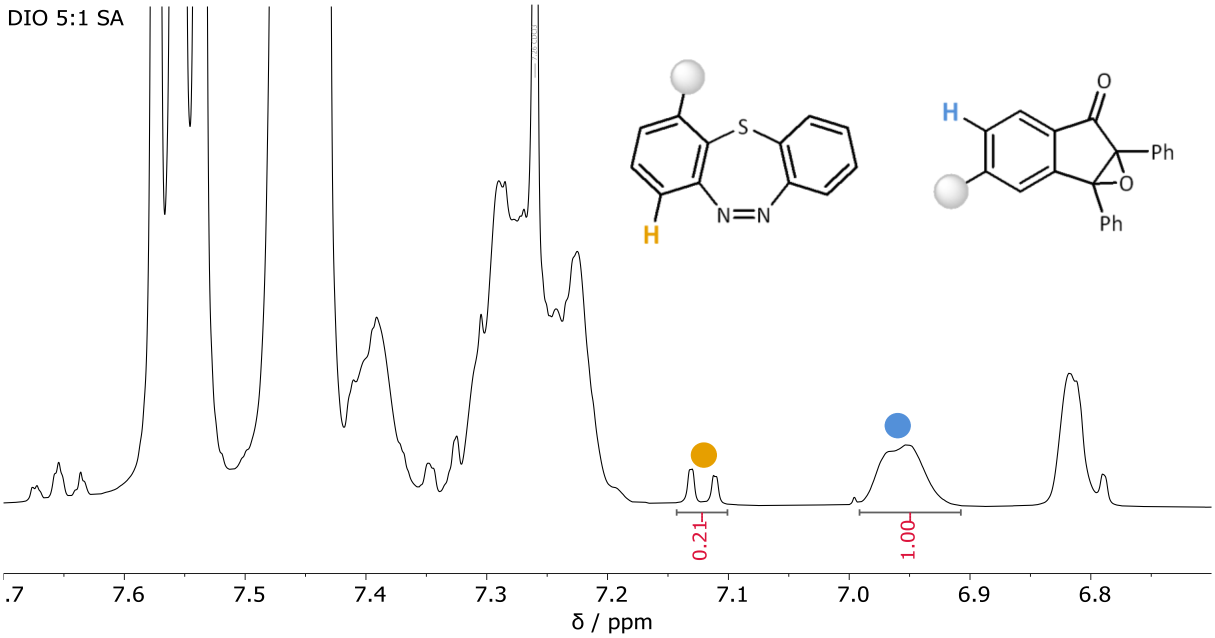

Figure S16. ^{1}H NMR (400 MHz) spectrum in $CDCl_3$ at ambient temperature of the photoresist formulation in acetophenone for two-color lithography with a DIO:SA ratio of 5:1 ([DIO] = 70.5 mM, [SA] = 14.1 mM).

5 Polymerization Quenching Effect of SA

No polymer formation was observed when we tried to prepare a polymer under the same conditions as polyDIO (**SI Section 2.1.5**) with a methacrylated SA-OH instead of the DIO monomer. Test polymerizations of EHA with different amounts of unmodified SA were performed in order to investigate the impact of SA on the polymerization, thermally initiated by AIBN. For the reactions EHA (284 µL, 250 mg, 1.36 mmol, 1.00 eq.), AIBN (1.34 mg, 8.14 µmol, 0.006 eq.) were dissolved in 0.9 mL toluene. Subsequently, 0, 0.5 or 5.5mol% SA were added, and the solutions were sparged with nitrogen. The polymerizations were then stirred at 65 °C for 20 hours and the crude mixtures were analyzed via NMR (**Figure S17**).

The progress of the polymerization was monitored via ^{1}H NMR spectroscopy, specifically by observing the resonance of the proton attached to the tertiary carbon of EHA, which appears at 4.08 ppm in the monomer. Upon polymerization, this resonance shifts upfield to 3.93 ppm and significantly broadens, indicating incorporation into the polymer backbone. In the absence of SA, this resonance nearly disappears (**Figure S17**, blue trace), suggesting near-complete monomer conversion. In contrast, when 0.5 mol% SA was present, a more prominent residual signal at 4.08 ppm was observed (**Figure S17**, yellow trace), indicative of a reduced polymerization rate. At an SA concentration of 5.5 mol%, no significant changes in the monomer signal and no polymer signal were detected (**Figure S17**, orange trace), confirming complete inhibition of polymer formation and demonstrating the quenching effect of SA on the radical polymerization process.

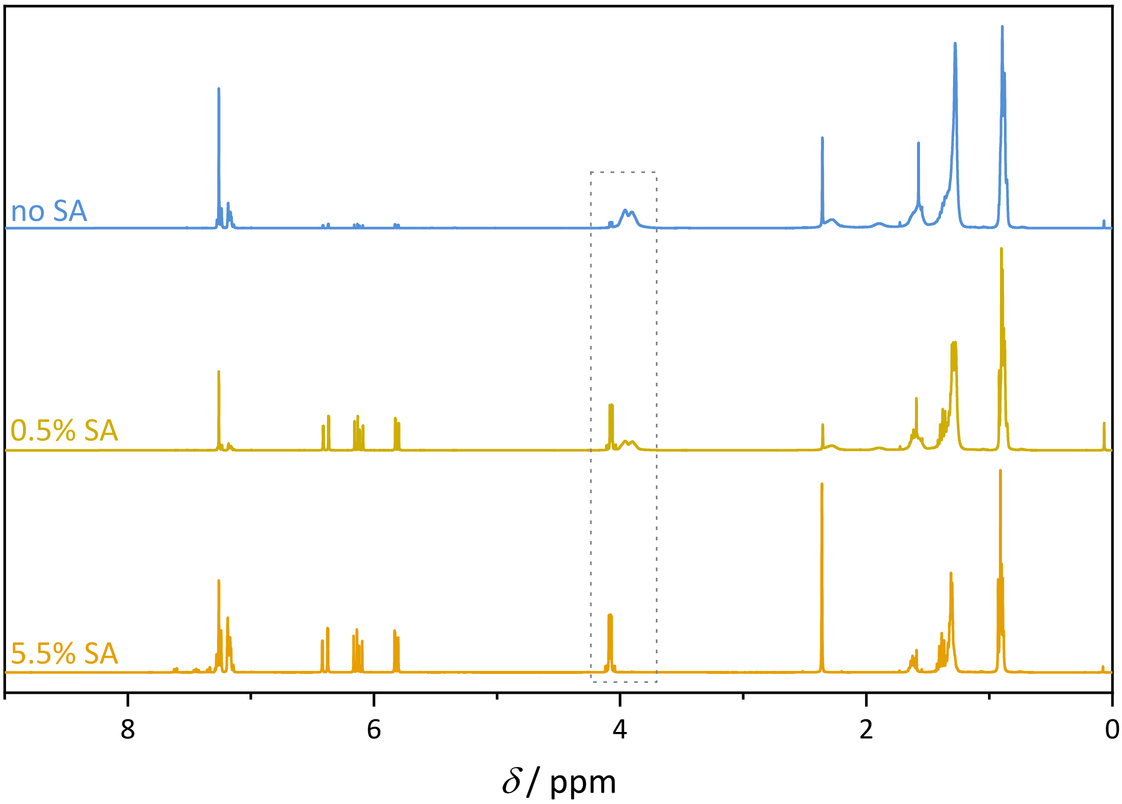

Figure S17. ^{1}H NMR spectra of the polymerizations of EHA in toluene at 65 °C after 20 hours in the presence of 0, 0.5 and 5.5mol% SA, showing a radical quenching by the SA moiety.

6 Incorporation Ratio of DIO in PolyDIO

The incorporation ratio x_{DIO} of DIO to EHA in polyDIO was calculated via integration of magnetic resonance 1 for DIO and of the overlapping resonances 5, 10 and 23 for EHA (**Figure S18**) according to the following formula

$$x_{DIO} = \frac{I_1}{(I_{5,10,23} - 4 * I_1)/(2 * I_1)} = \frac{1}{(18.85 - 4)/(2)} = 13.5\%$$

where I_1 and $I_{5,10,23}$ are the integrated values of the corresponding magnetic resonances 1 and 5, 10, 23, respectively.

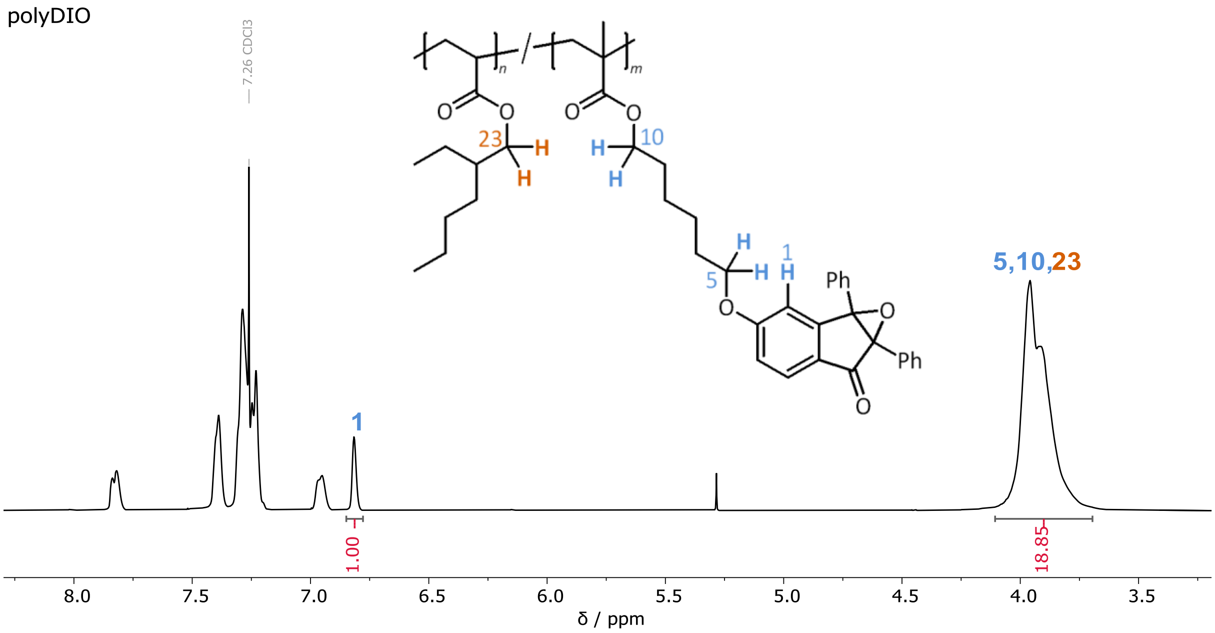

Figure S18. ^{1}H NMR (400 MHz) spectrum of **polyDIO** in $CDCl_3$ at ambient temperature. Highlighted in orange are the magnetic resonances of the EHA units and in blue of the DIO units that were used to calculate the DIO ratio.

7 NMR Spectra

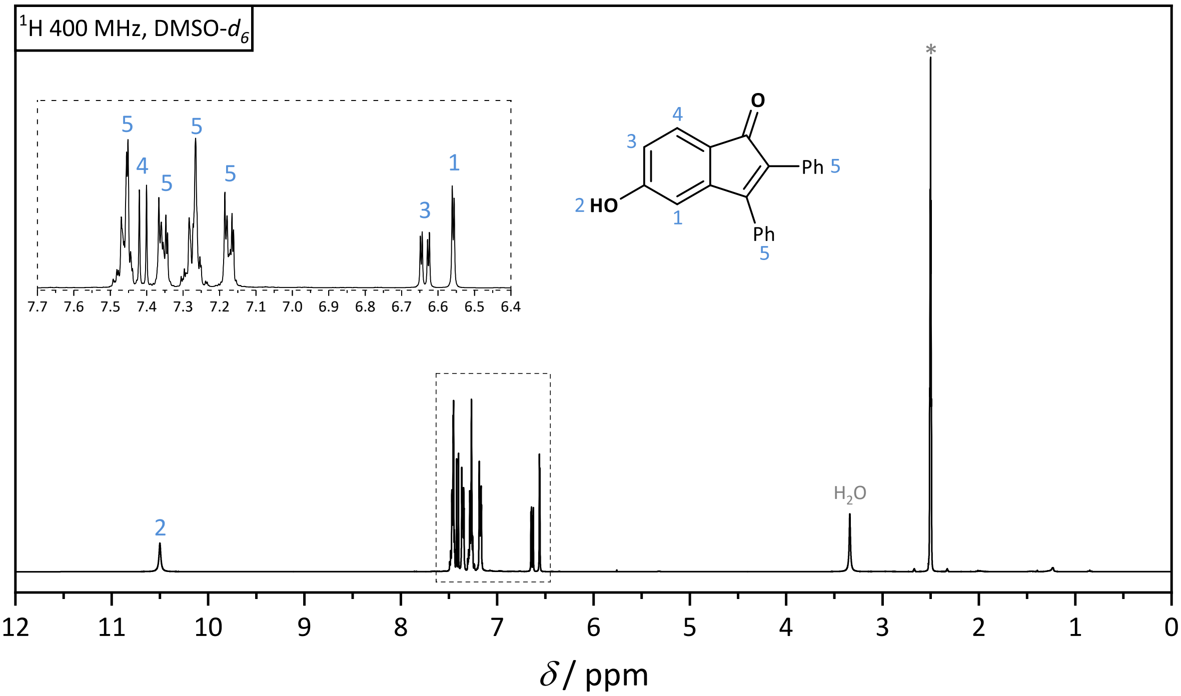

Figure S19. ^1H NMR (400 MHz) spectrum of **1** in DMSO-d_6 (*) at ambient temperature.

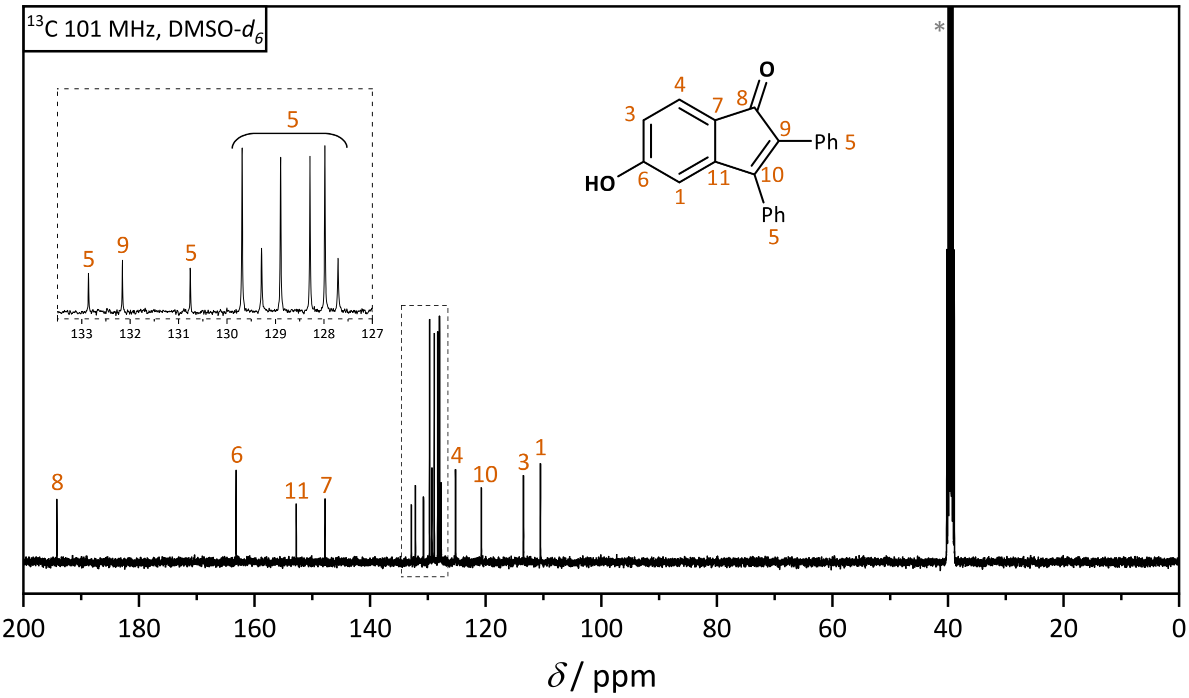

Figure S20. ^{13}C NMR (101 MHz) spectrum of **1** in DMSO-d_6 (*) at ambient temperature.

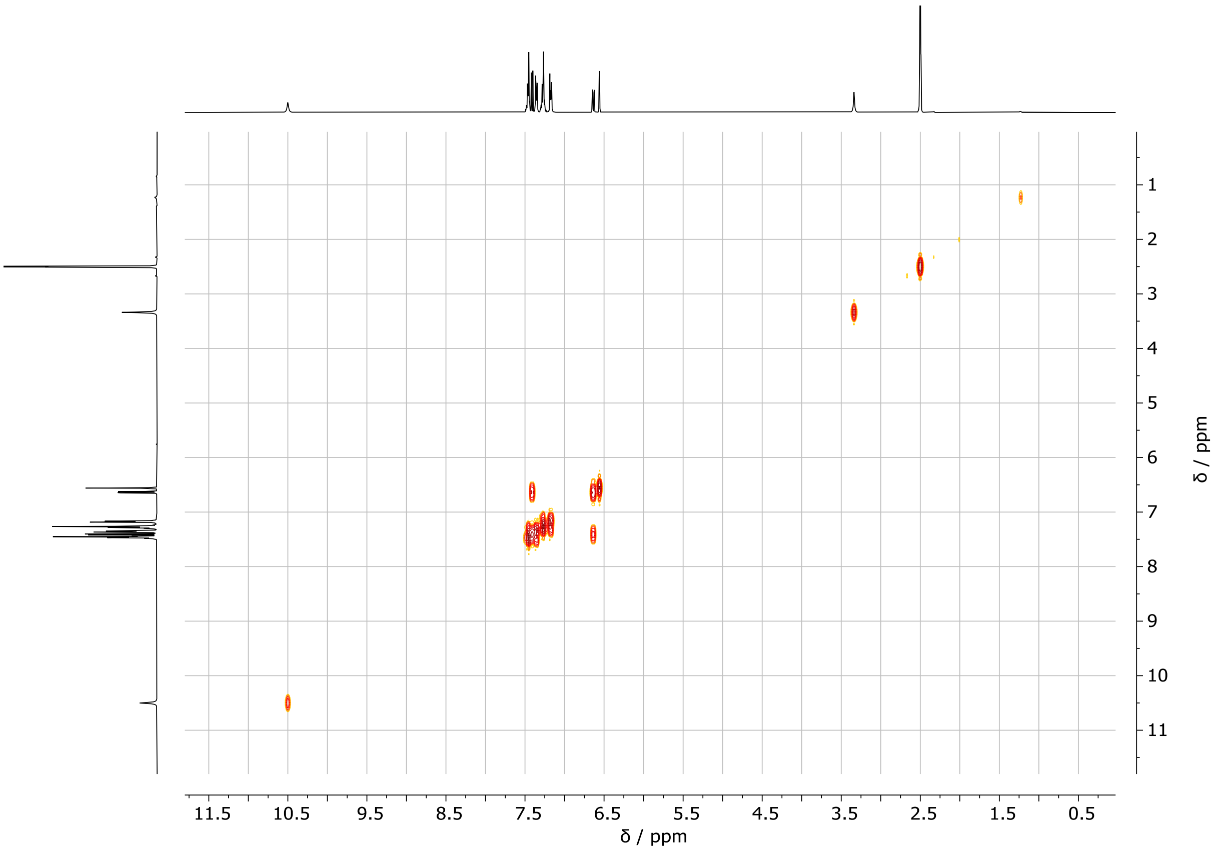

Figure S21. ^{1}H-^{1}H COSY (400 MHz) spectrum of **1** in DMSO-d_6 at ambient temperature.

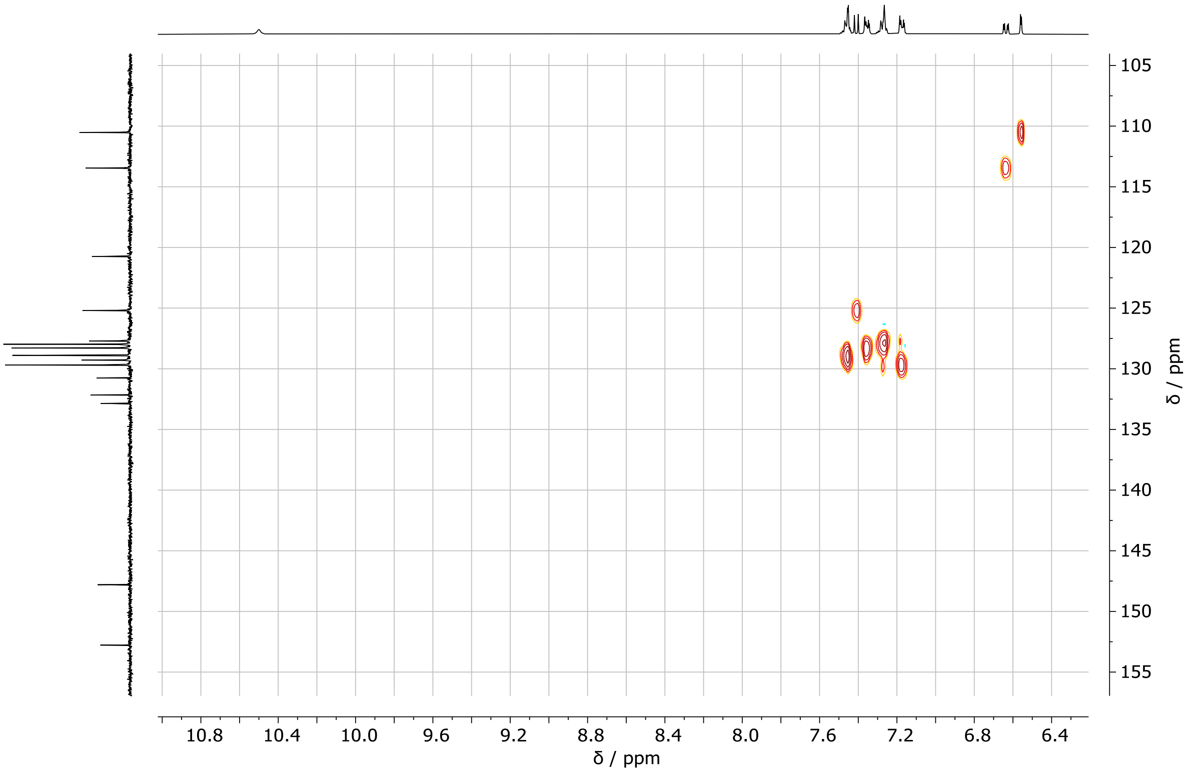

Figure S22. ^{1}H (400 MHz) - ^{13}C (101 MHz) HSQC spectrum of **1** in DMSO-d_6 at ambient temperature.

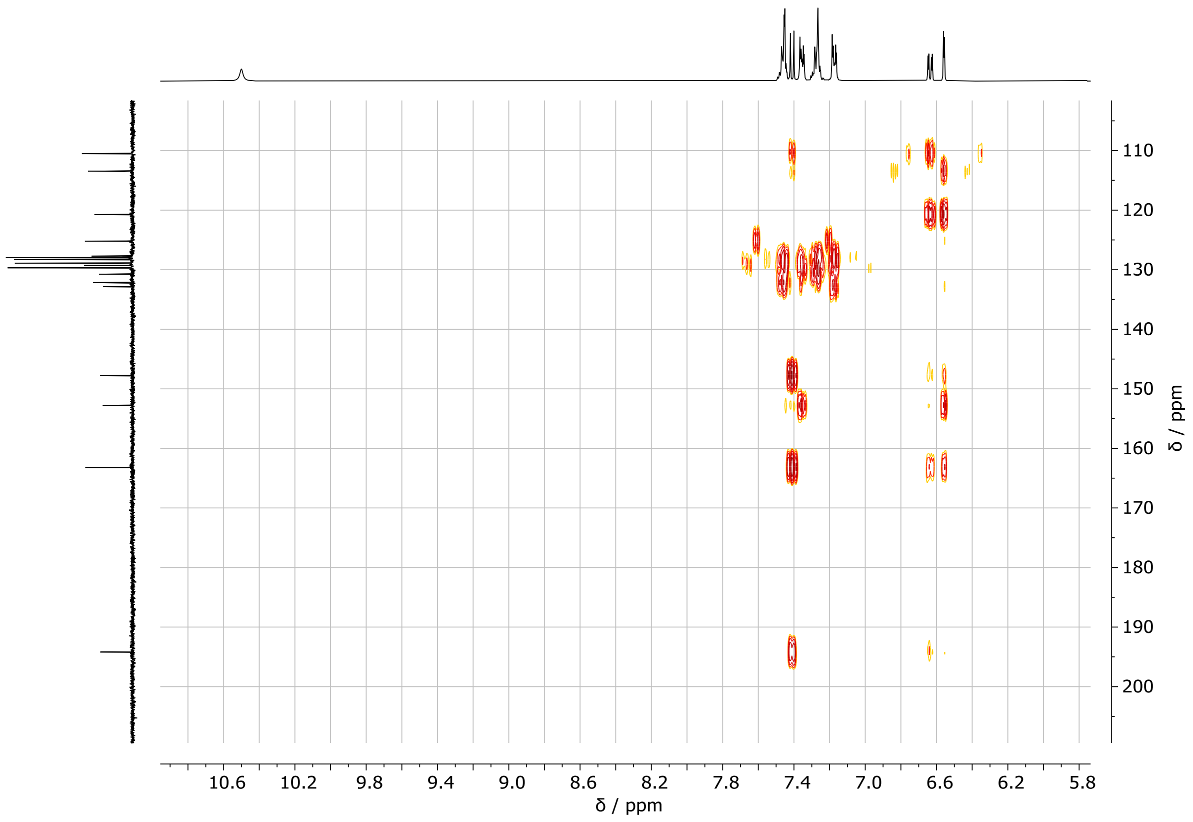

Figure S23. ^{1}H (400 MHz) - ^{13}C (101 MHz) HMBC spectrum of **1** in DMSO-d_6 at ambient temperature.

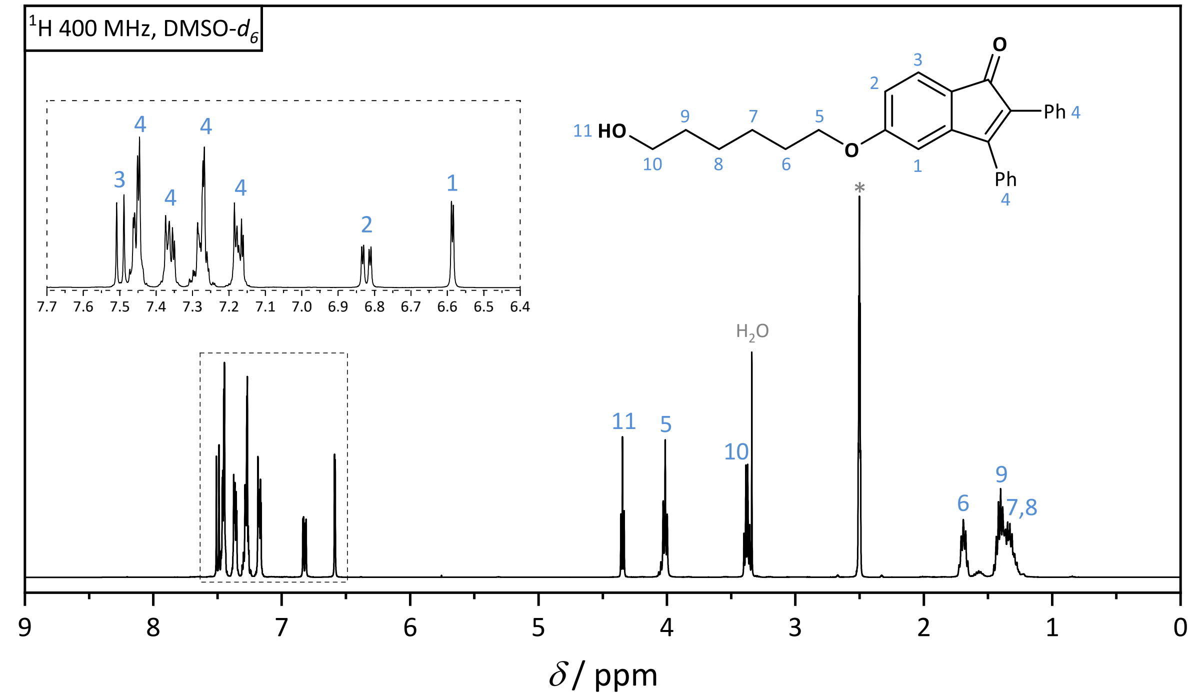

Figure S24. ^{1}H NMR (400 MHz) spectrum of **2** in DMSO-d_6 (*) at ambient temperature.

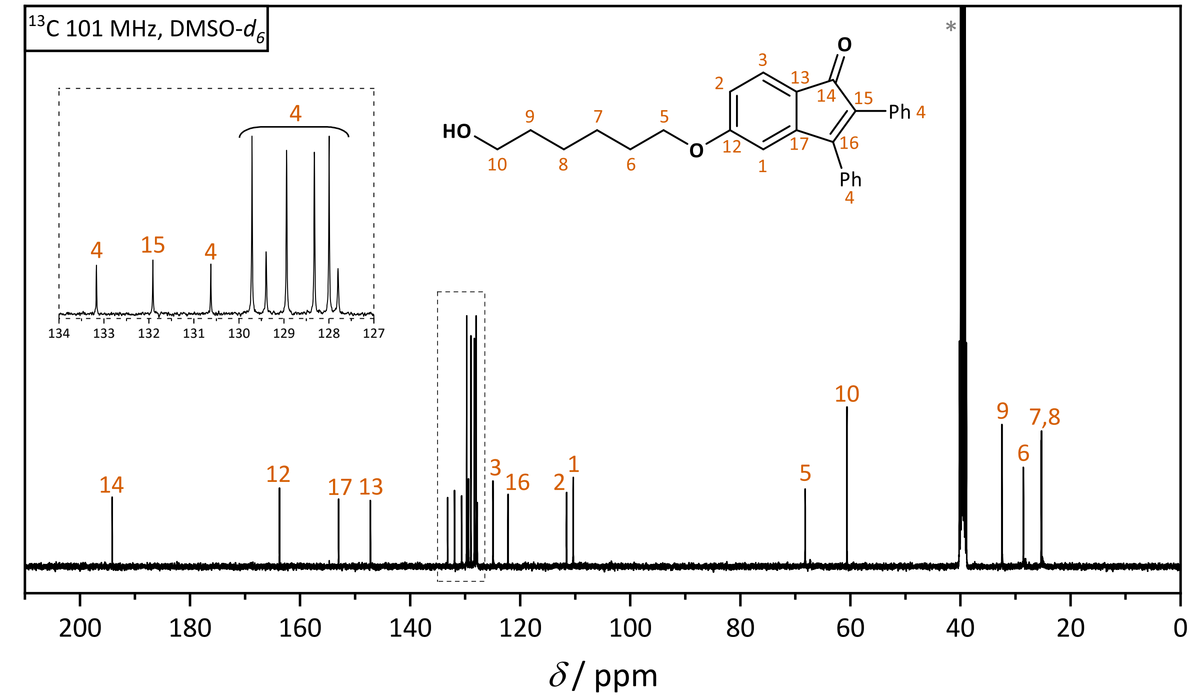

Figure S25. ^{13}C NMR (101 MHz) spectrum of **2** in DMSO-d_6 (*) at ambient temperature.

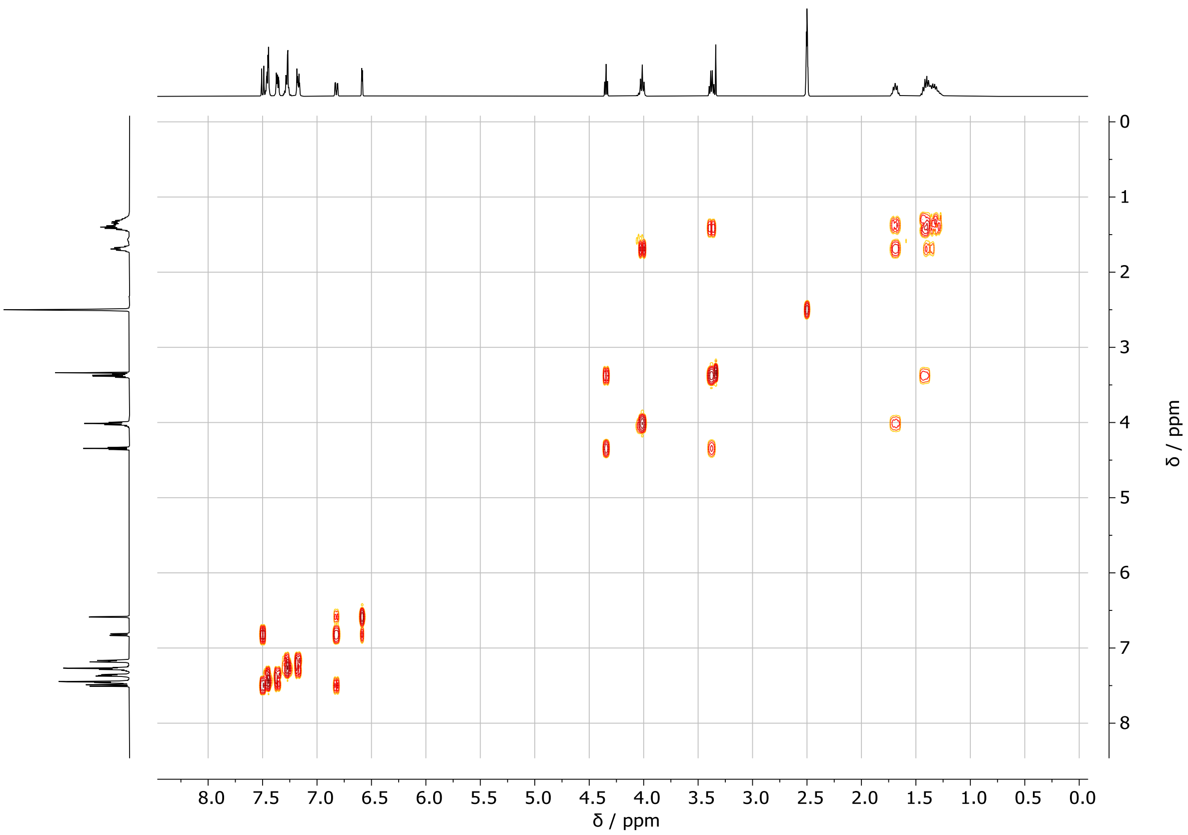

Figure S26. ^1H-^1H COSY (400 MHz) spectrum of **2** in DMSO-d_6 at ambient temperature.

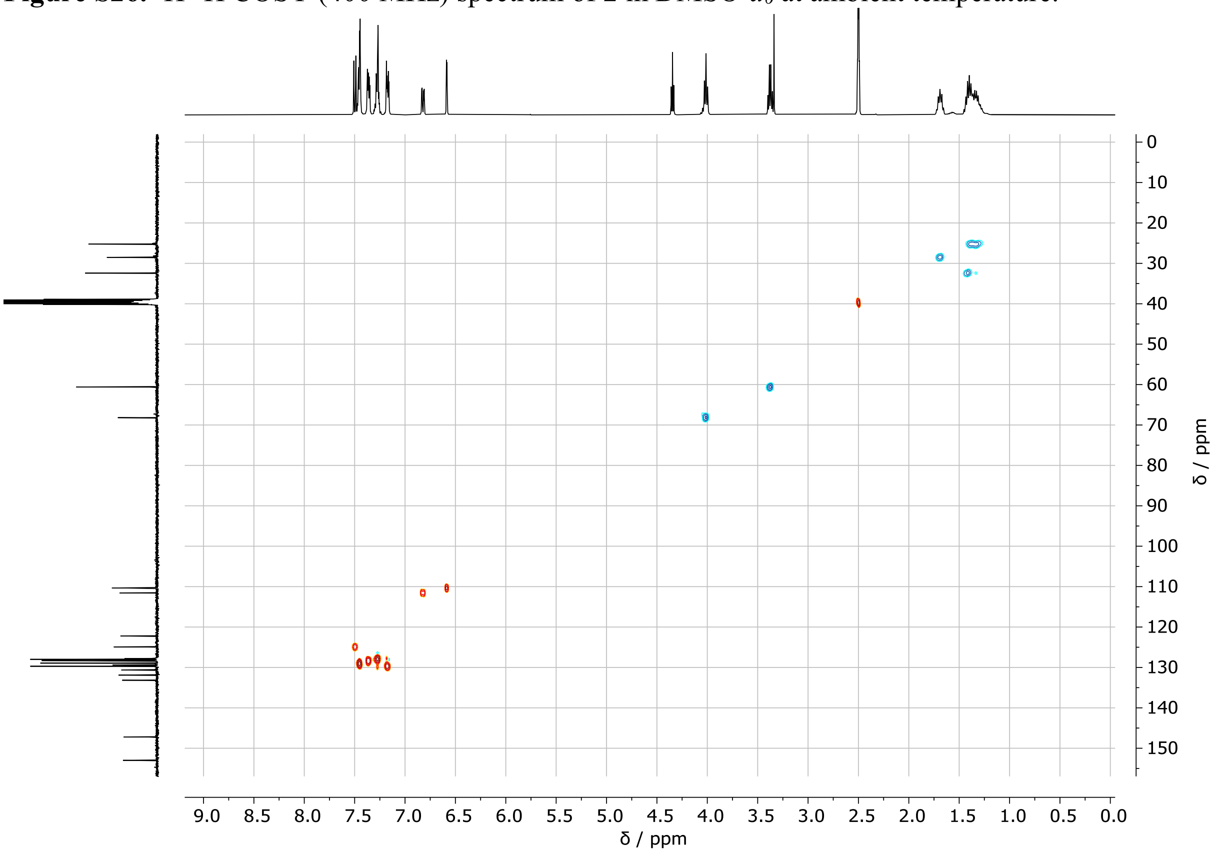

Figure S27. ^1H (400 MHz) - ^{13}C (101 MHz) HSQC spectrum of **2** in DMSO-d_6 at ambient temperature.

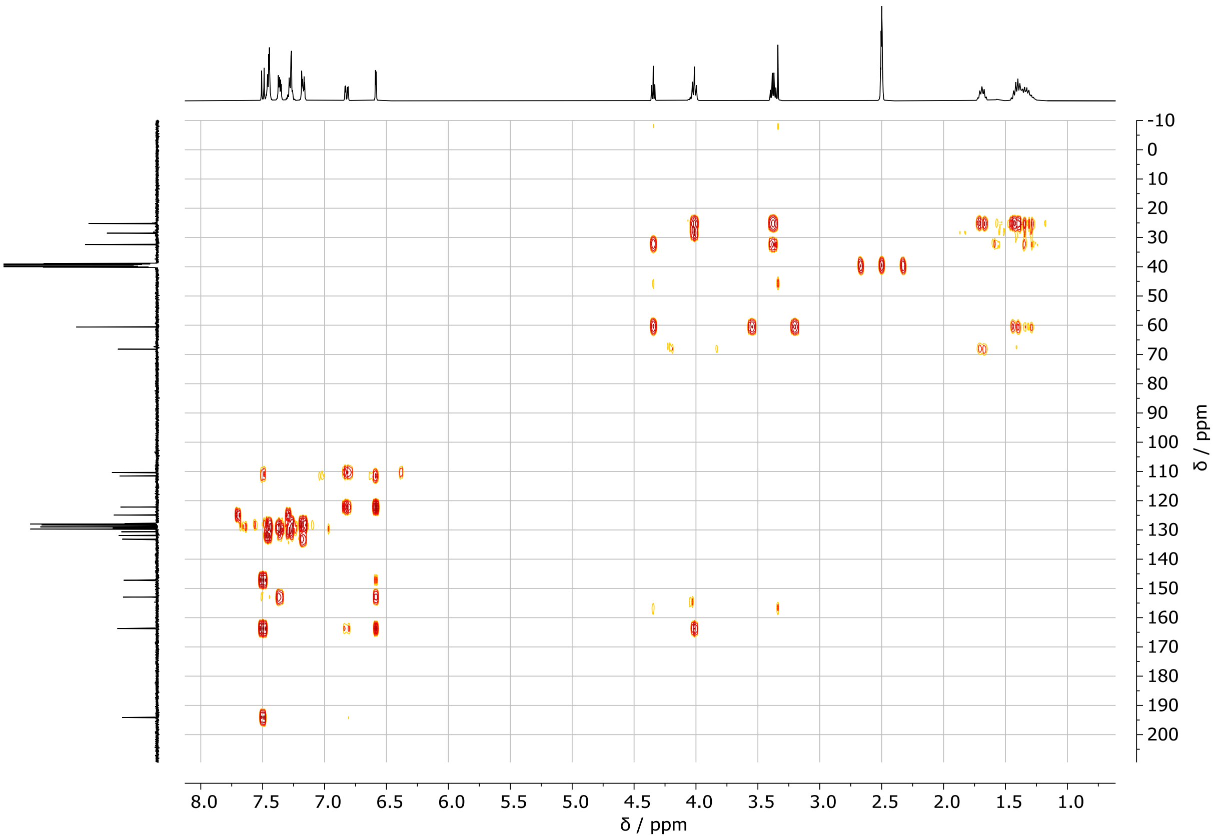

Figure S28. ^{1}H (400 MHz) - ^{13}C (101 MHz) HMBC spectrum of **2** in DMSO-d_6 at ambient temperature.

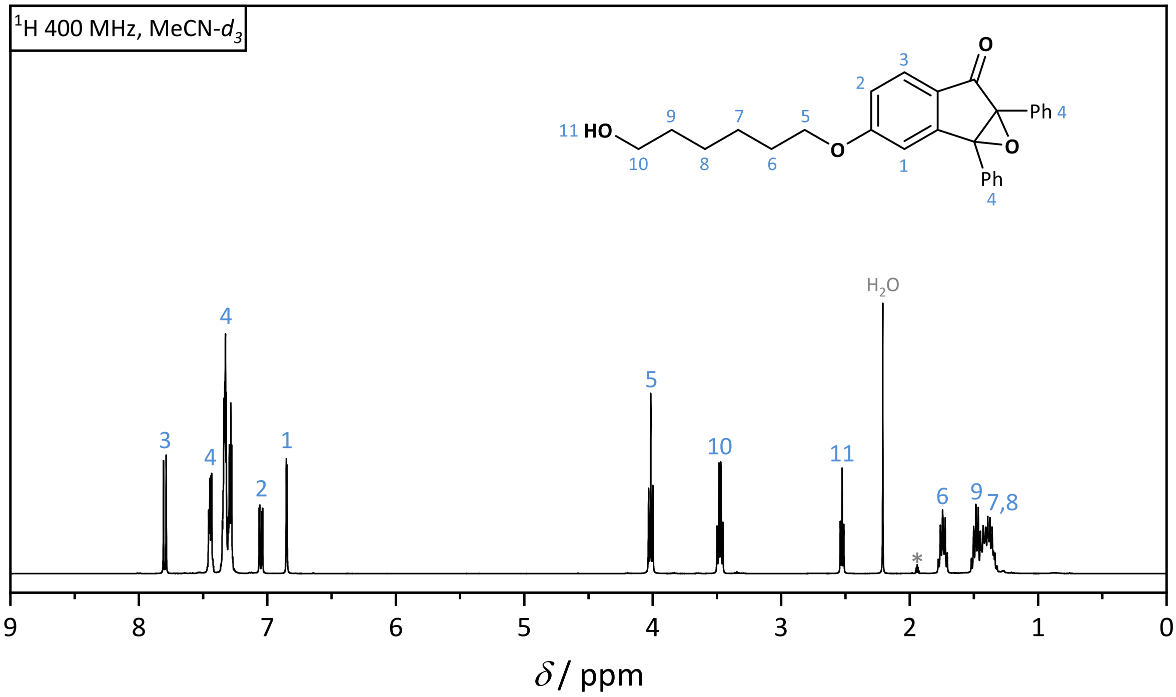

Figure S29. ^{1}H NMR (400 MHz) spectrum of DIO-OH in MeCN-d_3 (*) at ambient temperature.

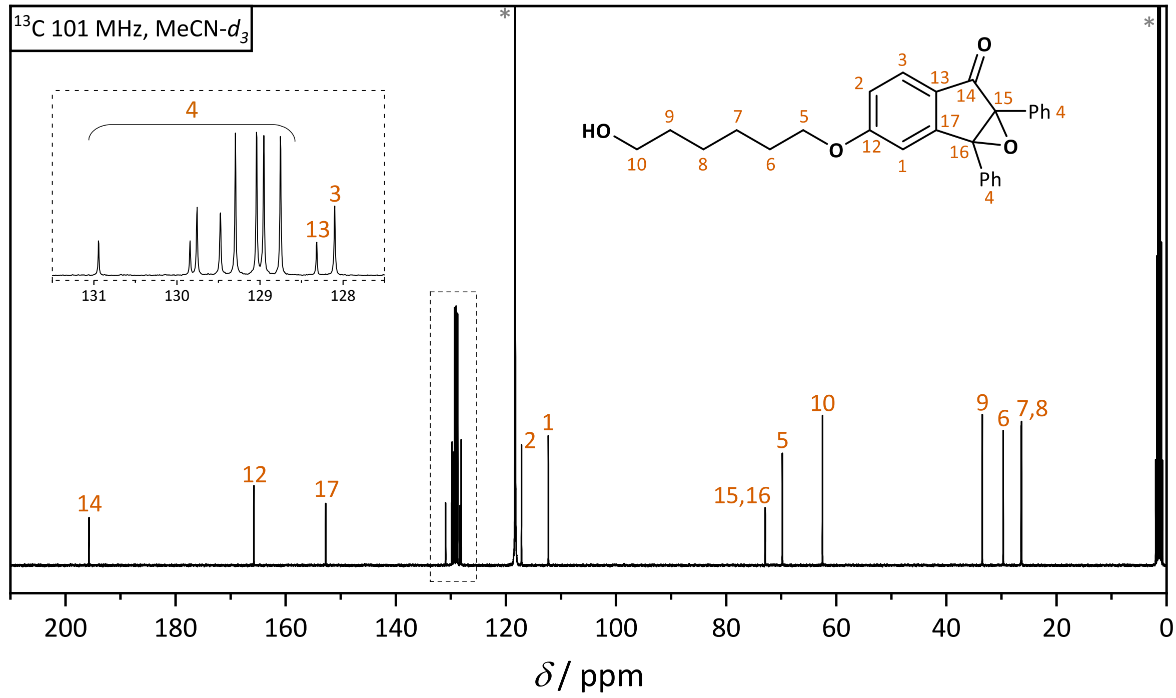

Figure S30. ^{13}C NMR (101 MHz) spectrum of DIO-OH in MeCN-d_3 (*) at ambient temperature.

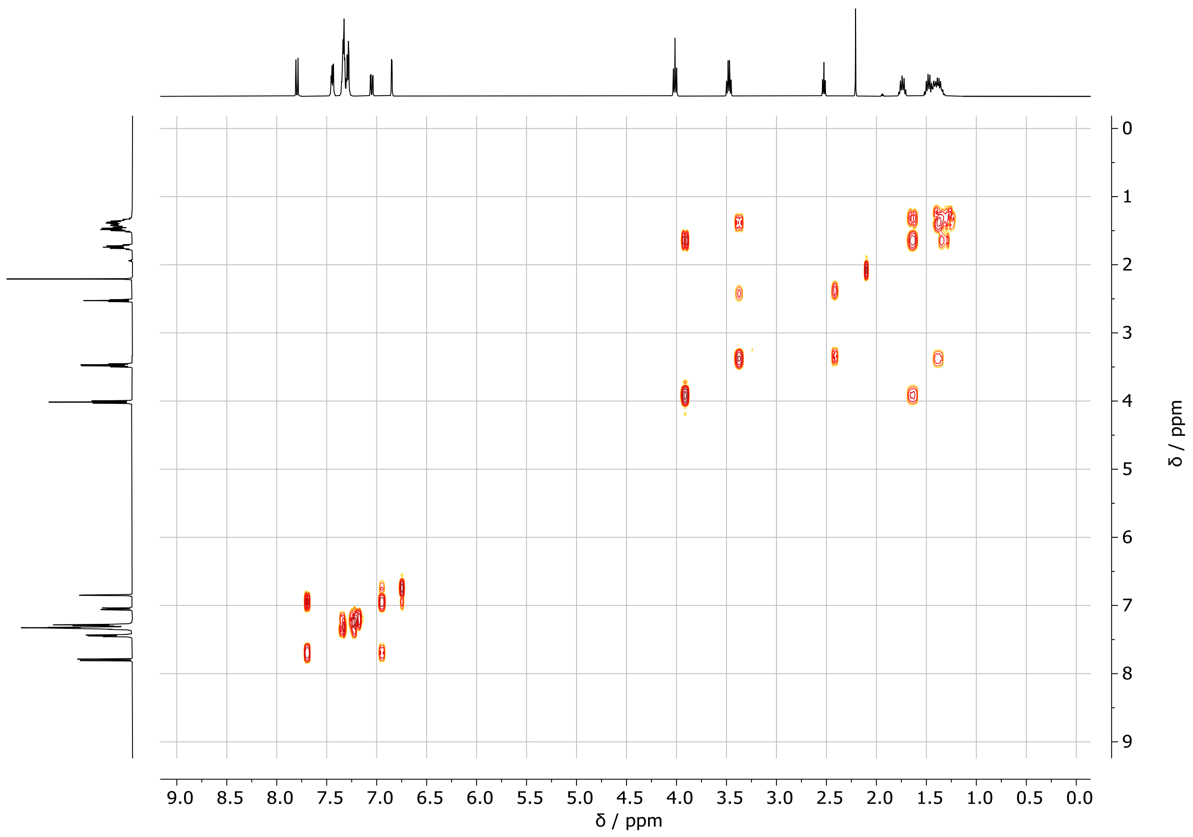

Figure S31. ^{1}H-^{1}H COSY (400 MHz) spectrum of DIO-OH in MeCN-d_3 at ambient temperature.

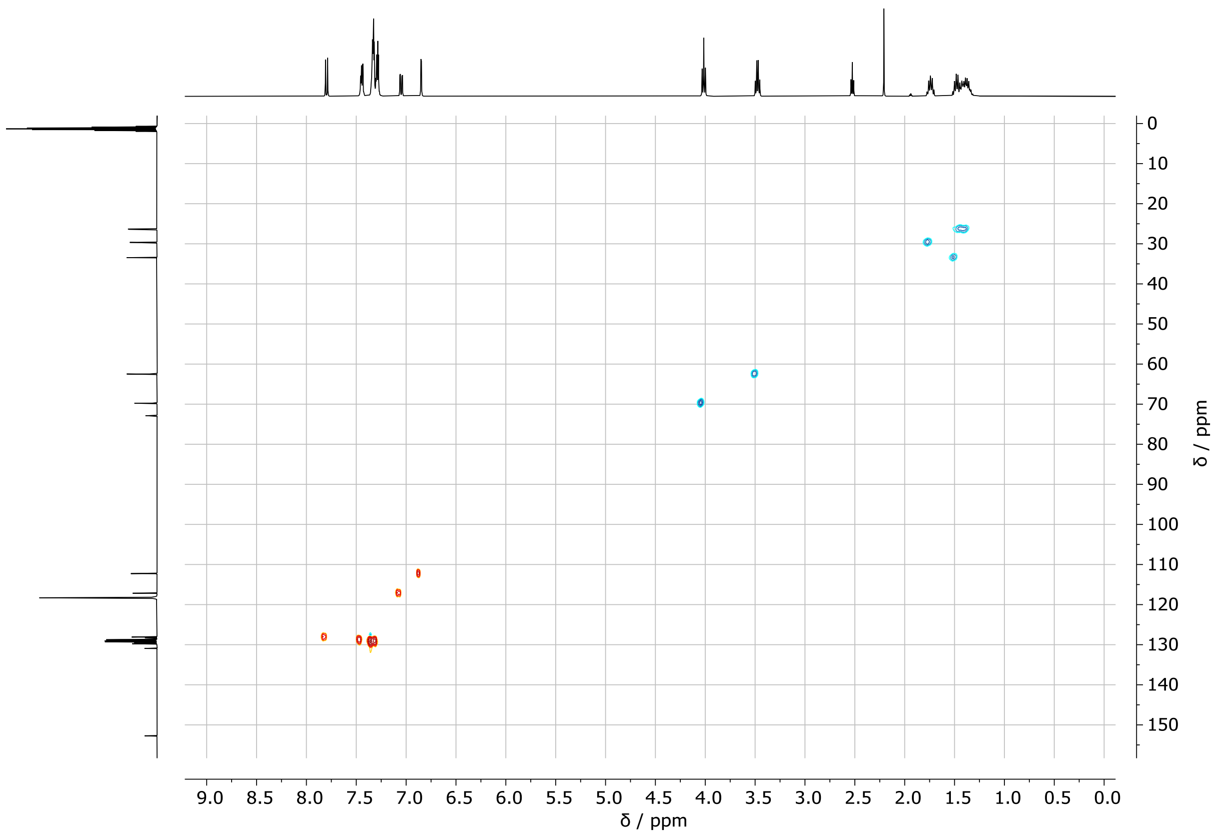

Figure S32. ^{1}H (400 MHz) - ^{13}C (101 MHz) HSQC spectrum of DIO-OH in MeCN-d_3 at ambient temperature.

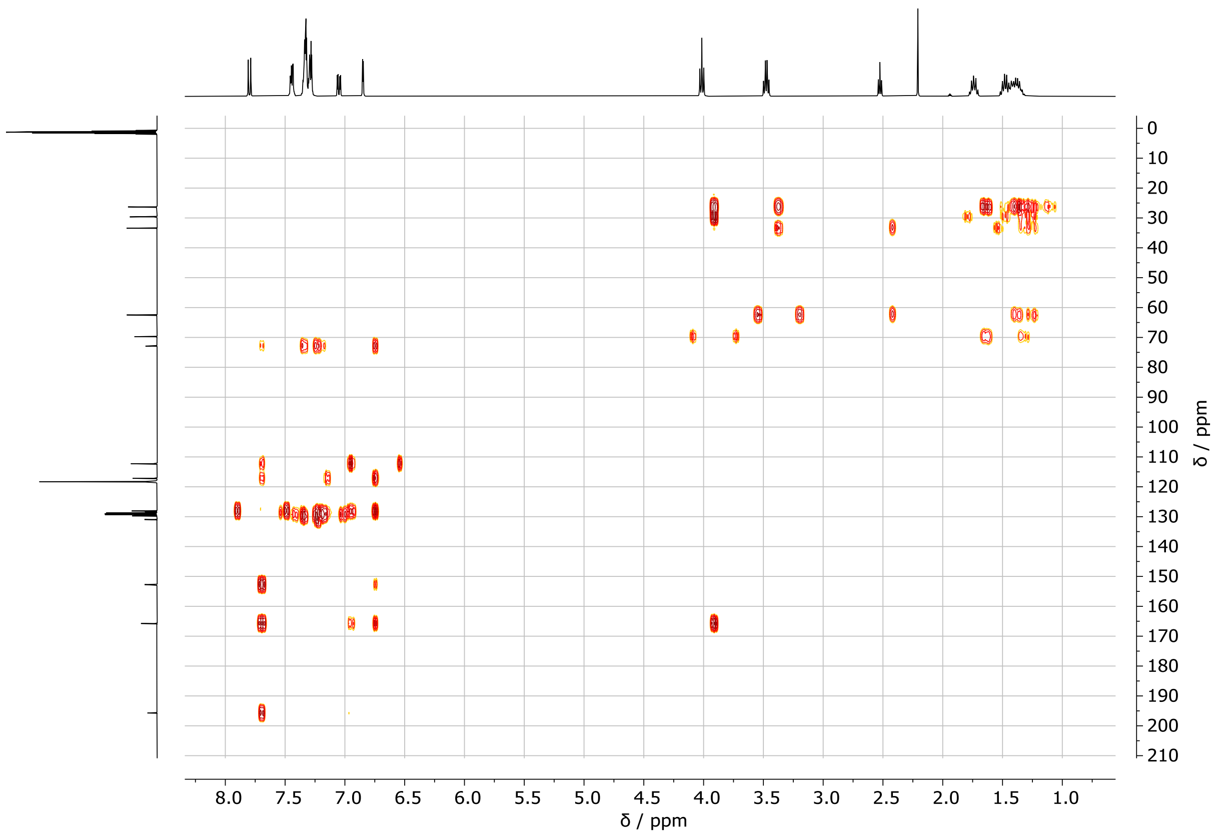

Figure S33. ^{1}H (400 MHz) - ^{13}C (101 MHz) HMBC spectrum of DIO-OH in MeCN-d_3 at ambient temperature.

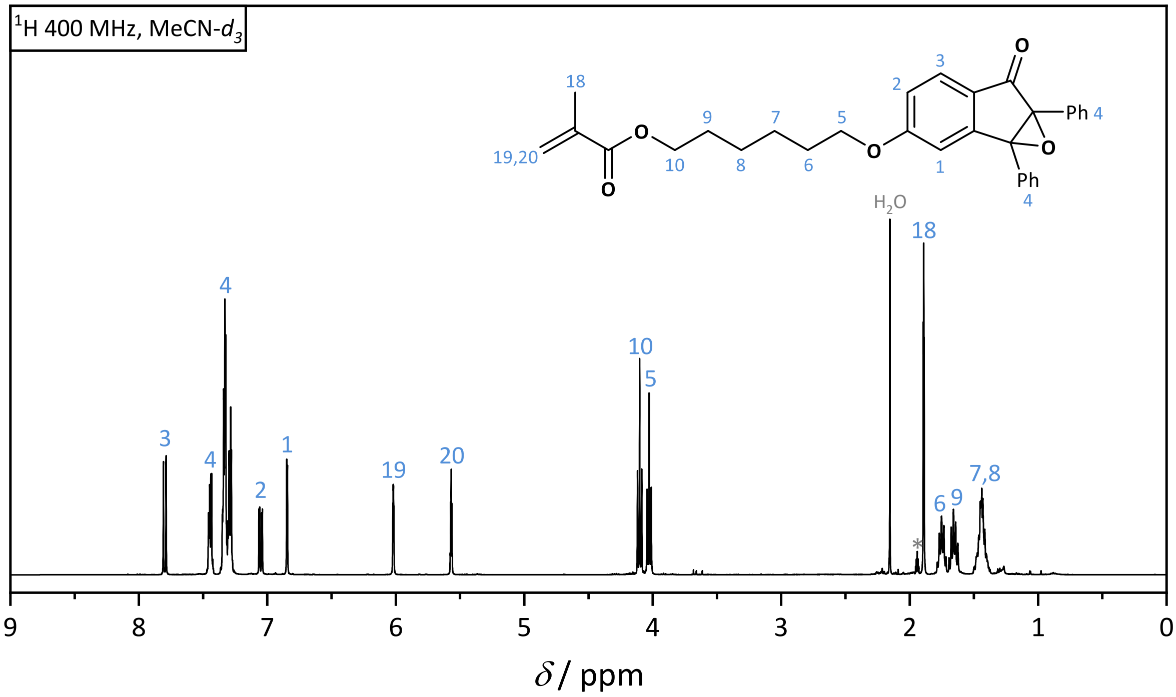

Figure S34. ^{1}H NMR (400 MHz) spectrum of **3** in MeCN-d_3 (*) at ambient temperature.

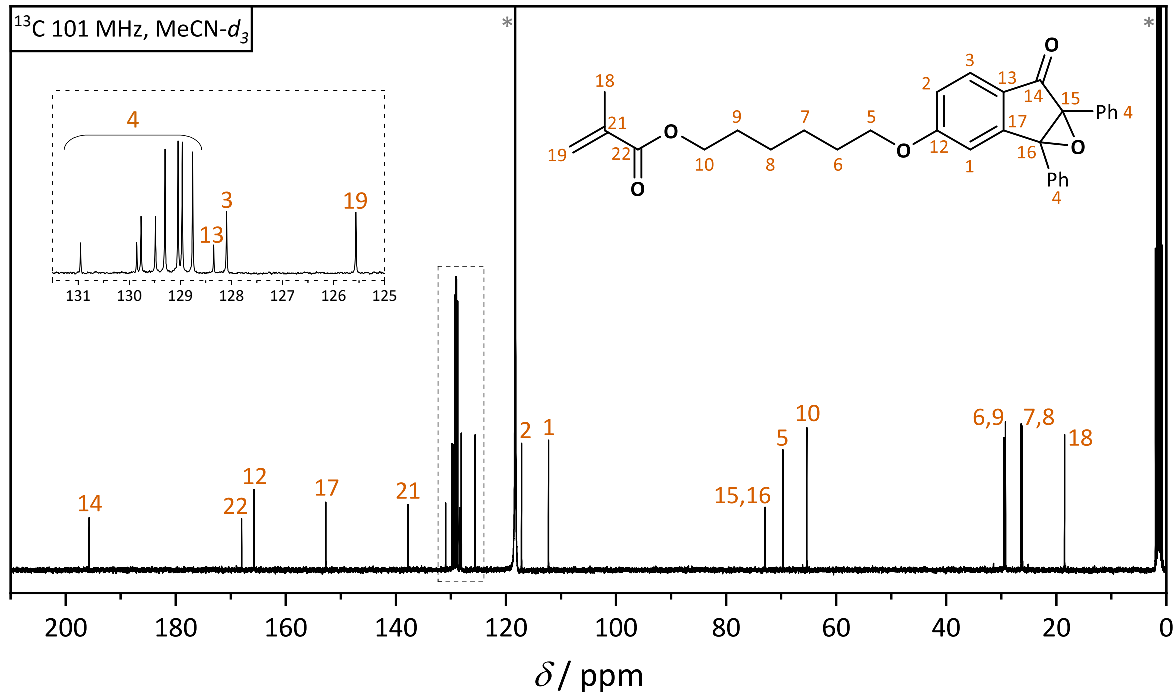

Figure S35. ^{13}C NMR (101 MHz) spectrum of **3** in MeCN-d_3 (*) at ambient temperature.

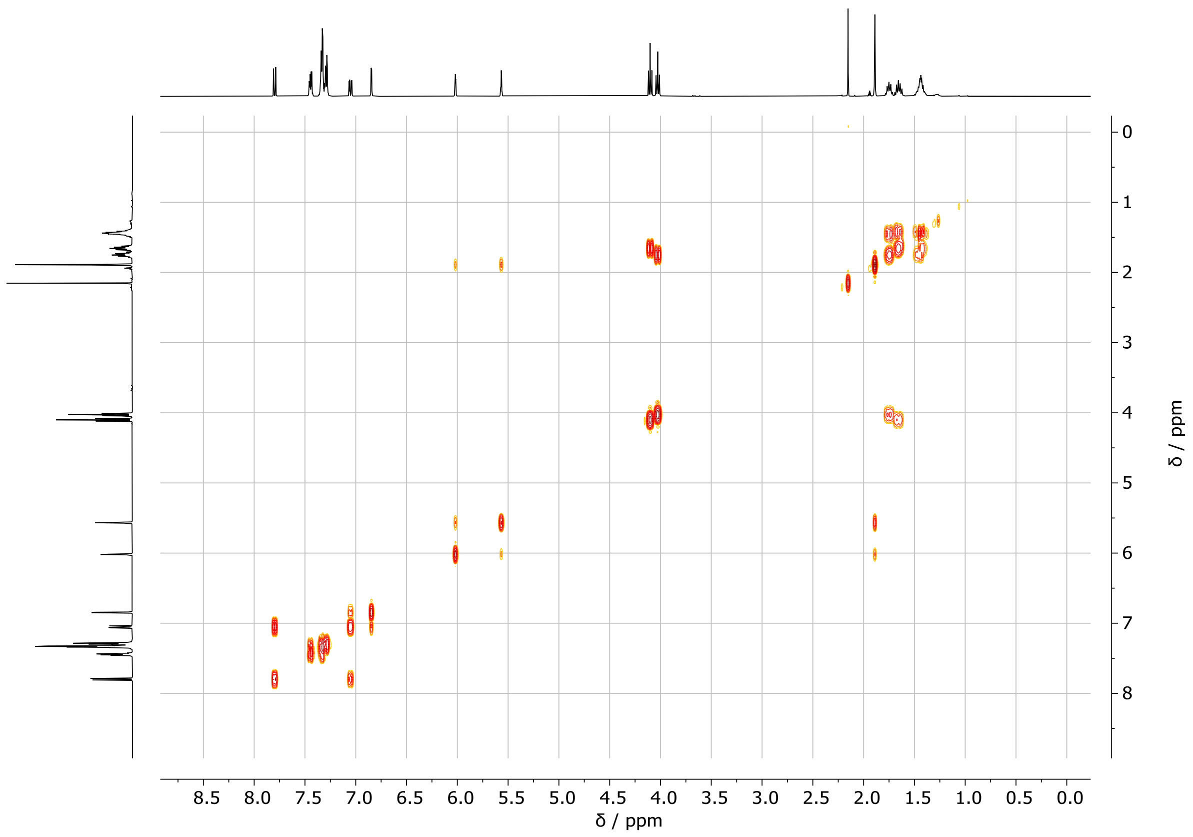

Figure S36. ^{1}H-^{1}H COSY (400 MHz) spectrum of **3** in MeCN-d_3 at ambient temperature.

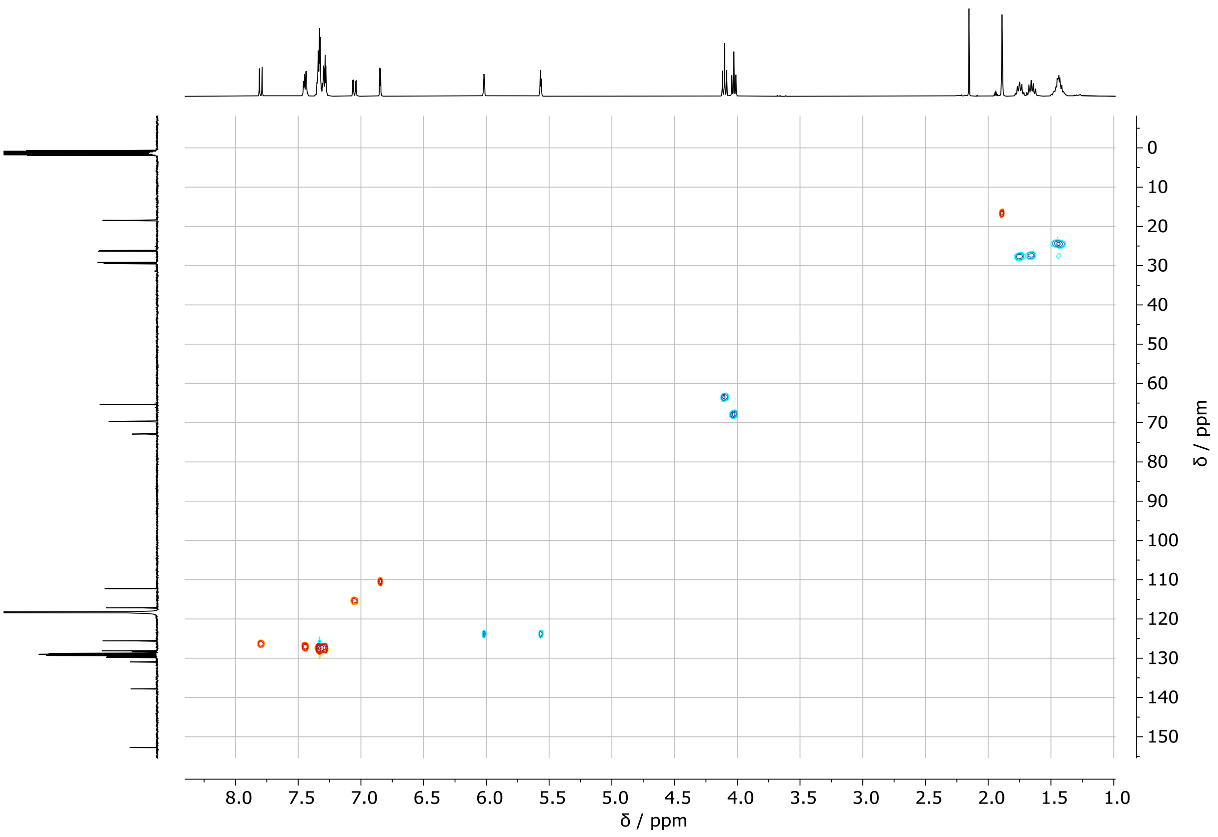

Figure S37. ^{1}H (400 MHz) - ^{13}C (101 MHz) HSQC spectrum of **3** in MeCN-d_3 at ambient temperature.

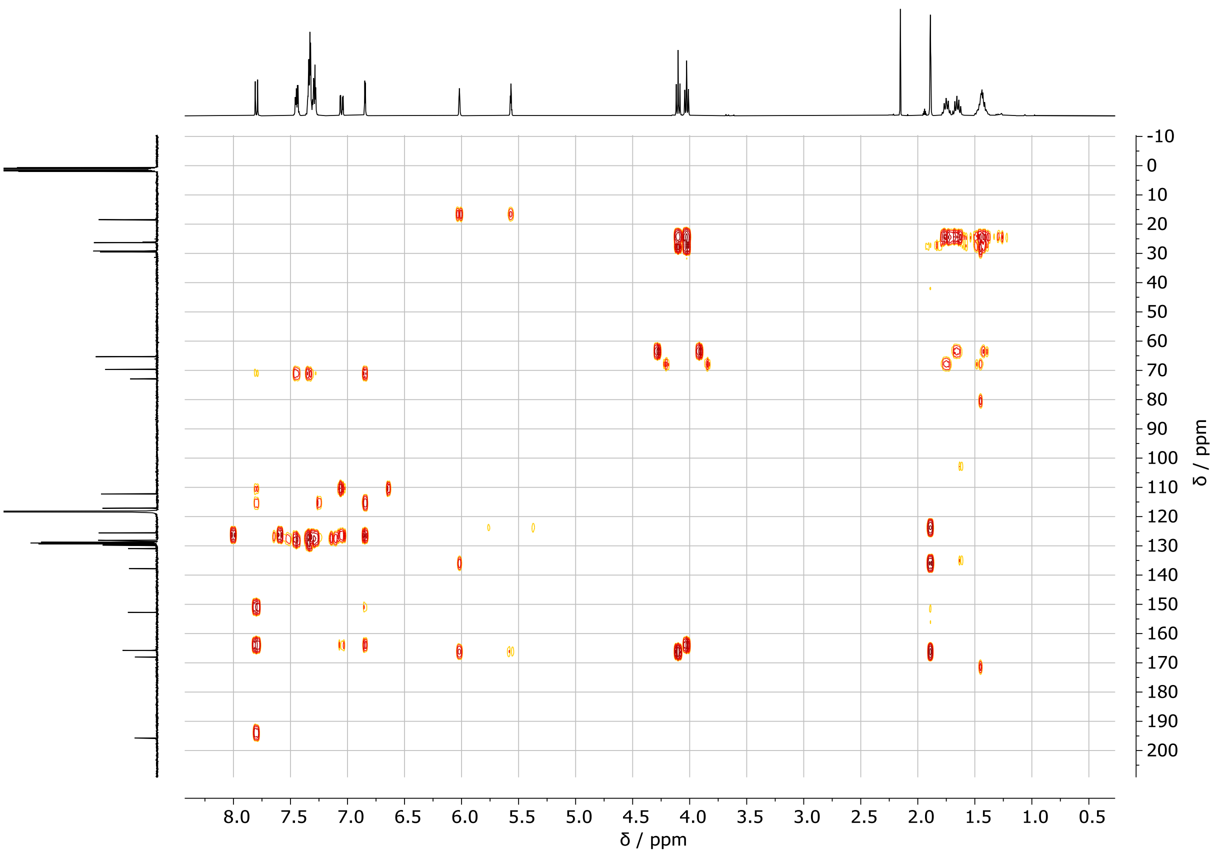

Figure S38. ^{1}H (400 MHz) - ^{13}C (101 MHz) HMBC spectrum of **3** in MeCN-d_3 at ambient temperature.

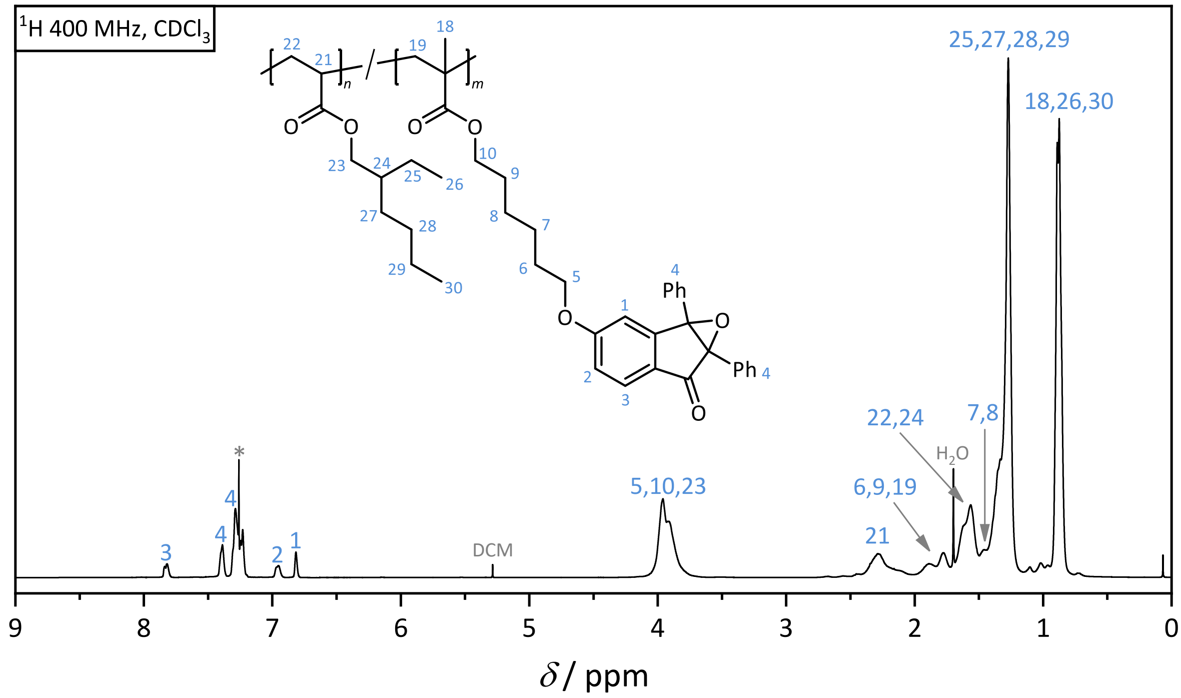

Figure S39. ^{1}H NMR (400 MHz) spectrum of polyDIO in $CDCl_3$ (*) at ambient temperature.

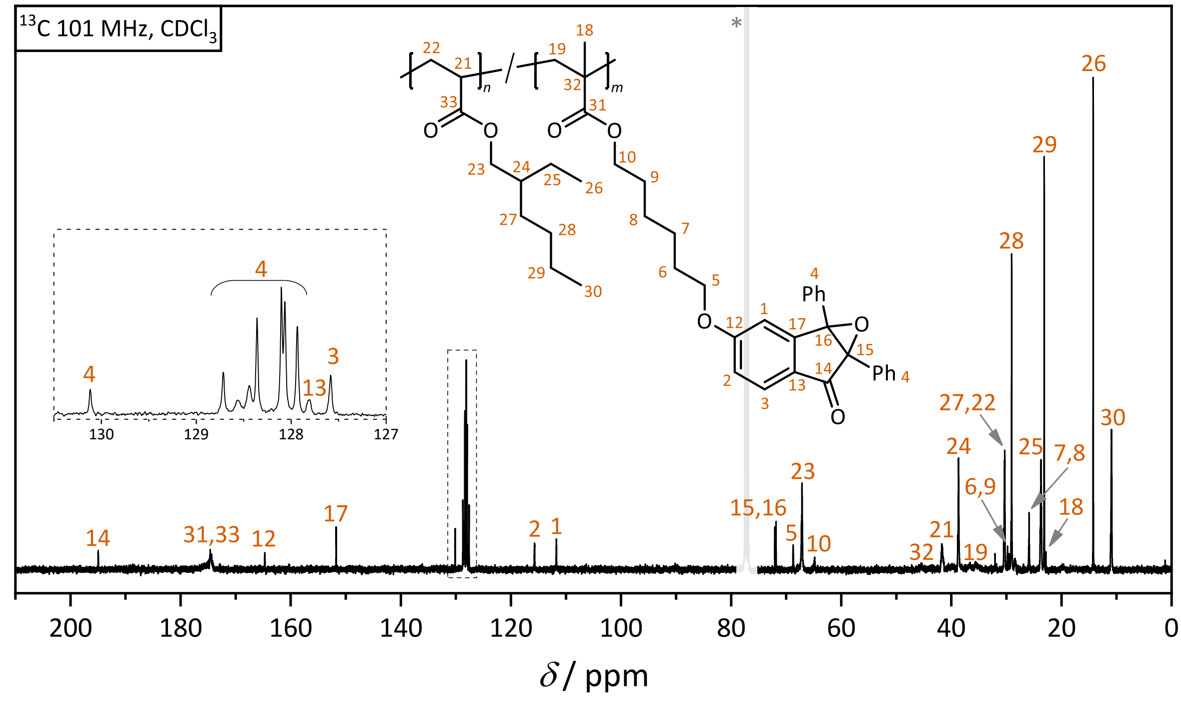

Figure S40. ^{13}C NMR (101 MHz) spectrum of polyDIO in $CDCl_3$ (*) at ambient temperature.

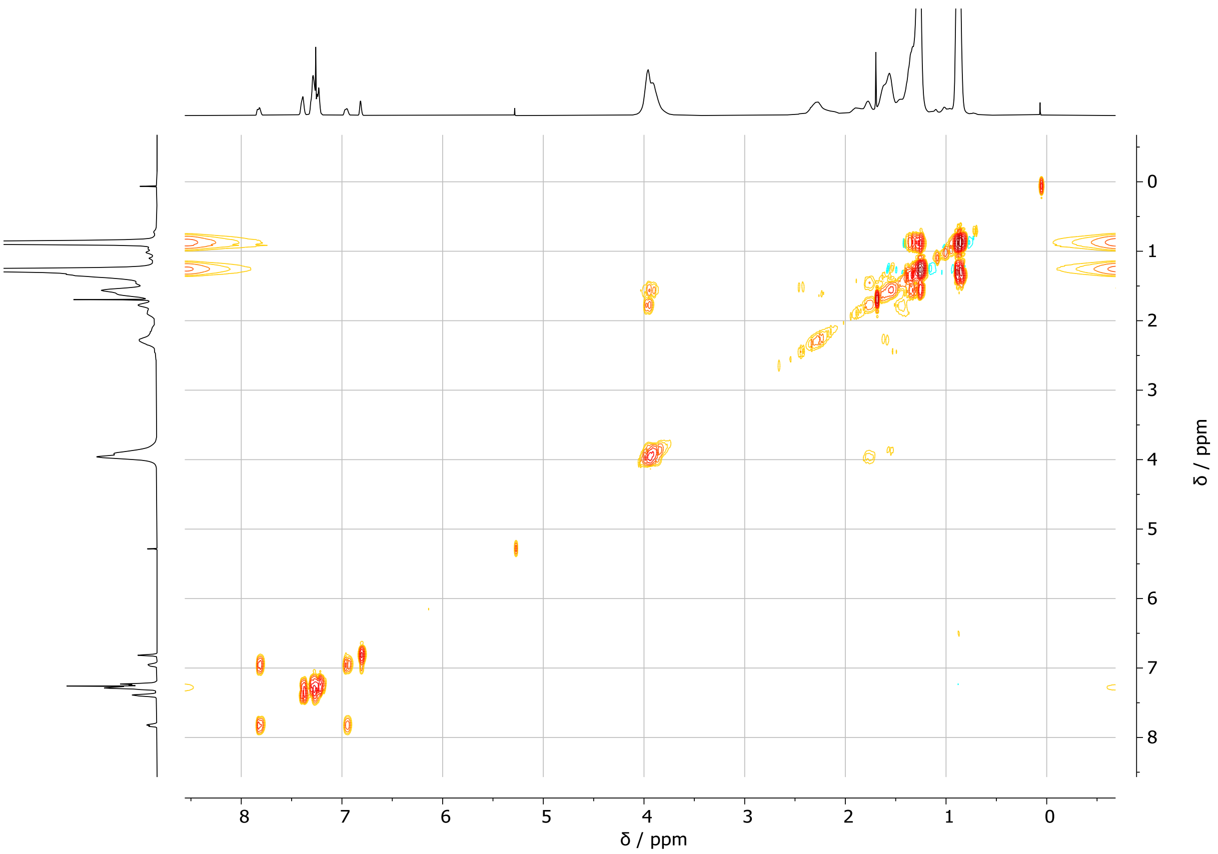

Figure S41. ^1H-^1H COSY (400 MHz) spectrum of polyDIO in $CDCl_3$ at ambient temperature.

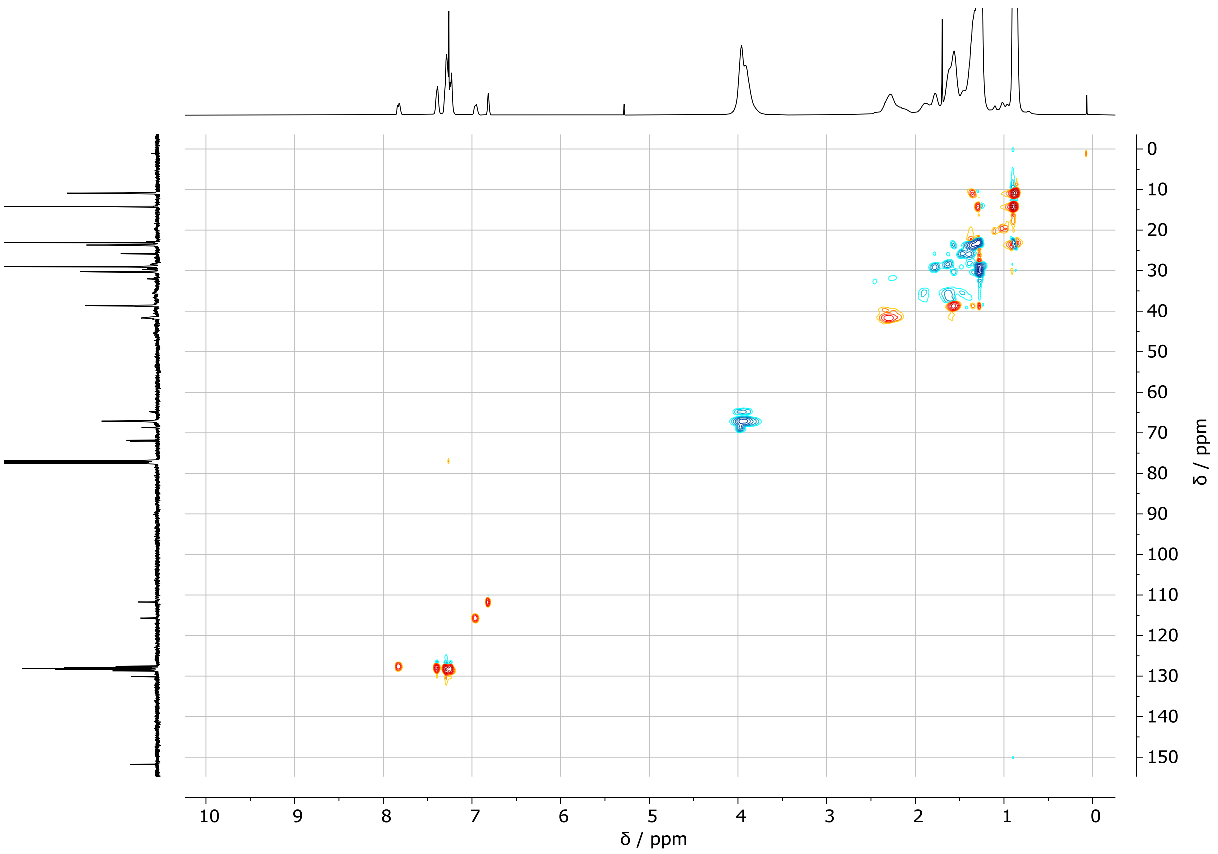

Figure S42. ^1H (400 MHz) - ^{13}C (101 MHz) HSQC spectrum of polyDIO in $CDCl_3$ at ambient temperature.

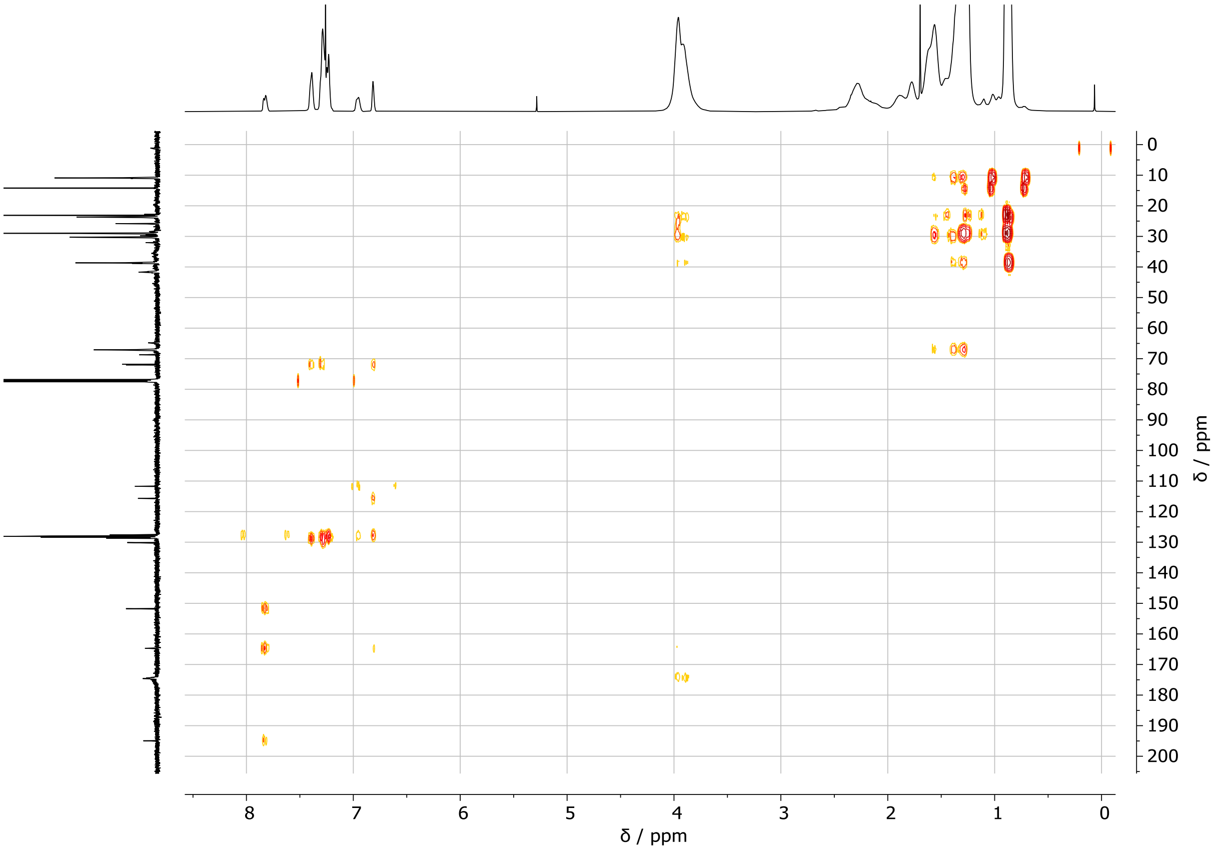

Figure S43. ^{1}H (400 MHz) - ^{13}C (101 MHz) HMBC spectrum of polyDIO in $CDCl_3$ at ambient temperature.

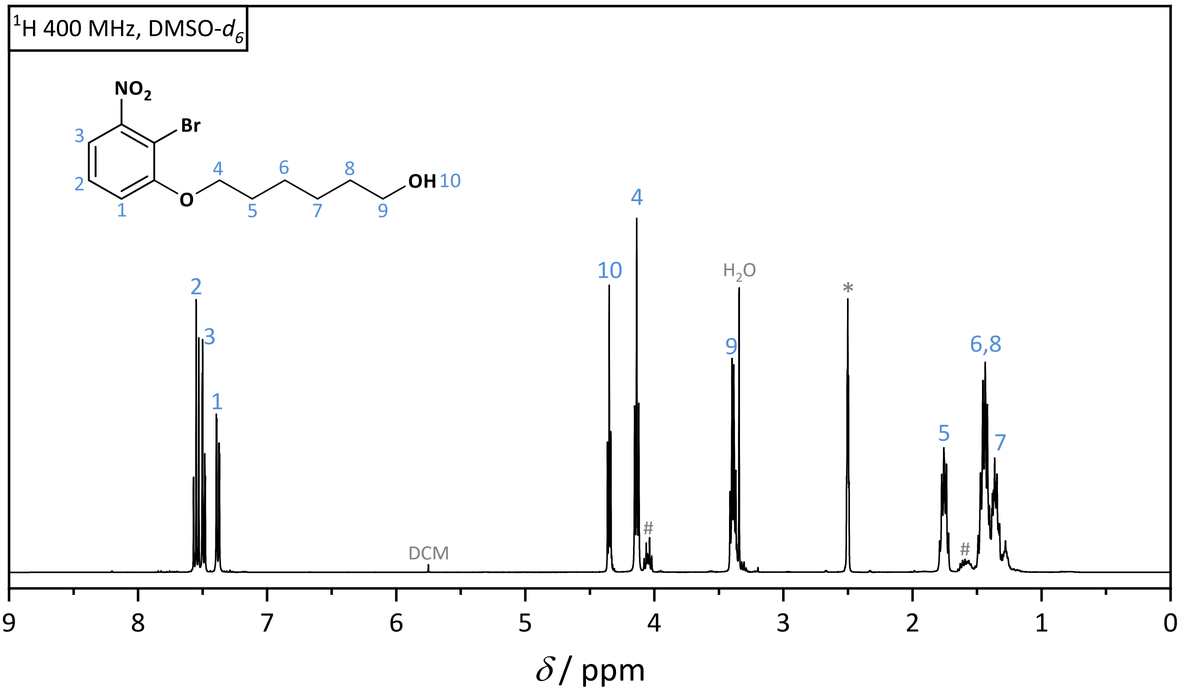

Figure S44. ^{1}H NMR (400 MHz) spectrum of **4** in DMSO-d_6 (*) at ambient temperature.

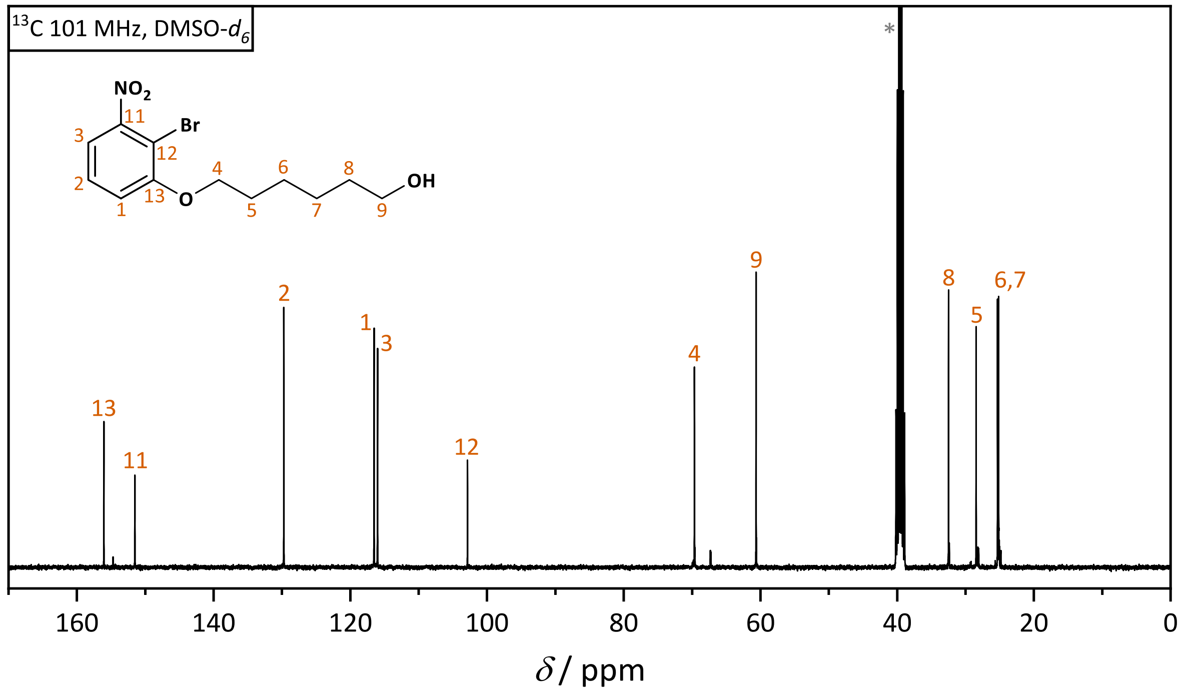

Figure S45. ^{13}C NMR (101 MHz) spectrum of **4** in DMSO-d_6 (*) at ambient temperature.

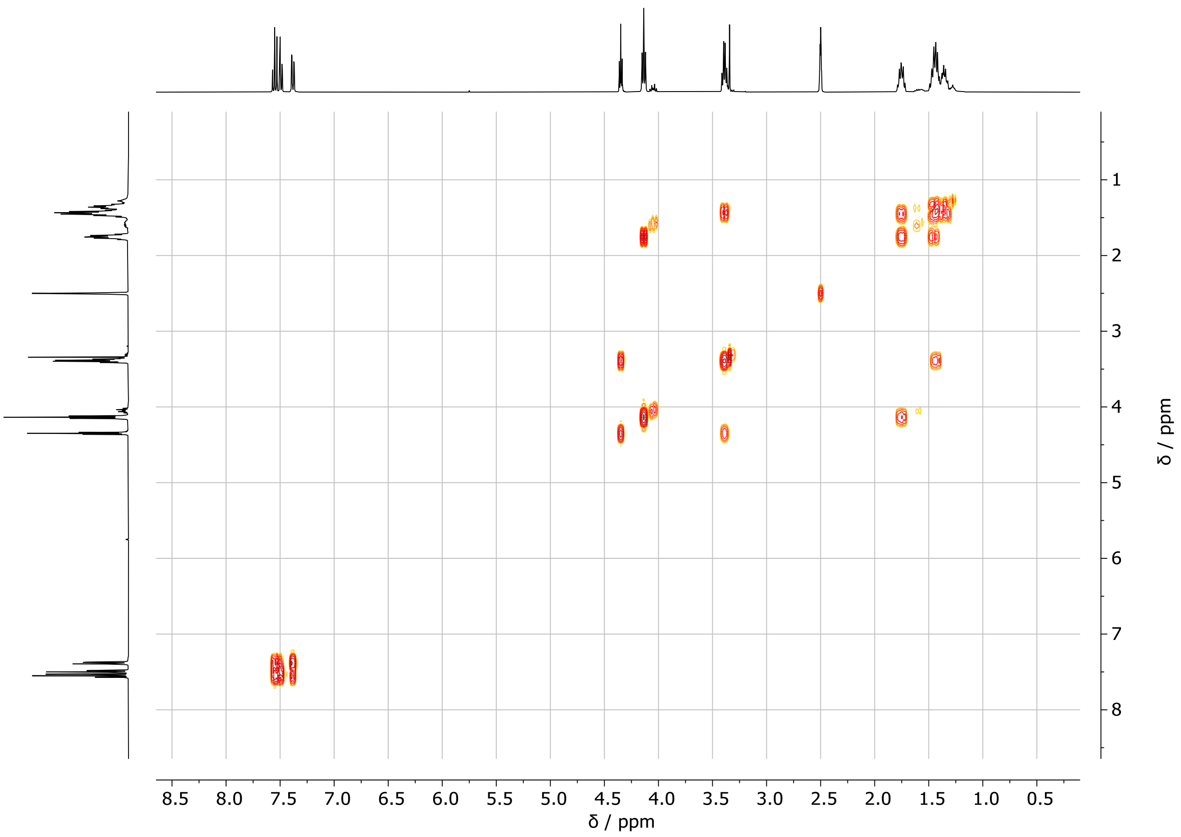

Figure S46. ^{1}H-^{1}H COSY (400 MHz) spectrum of **4** in DMSO-d_6 at ambient temperature.

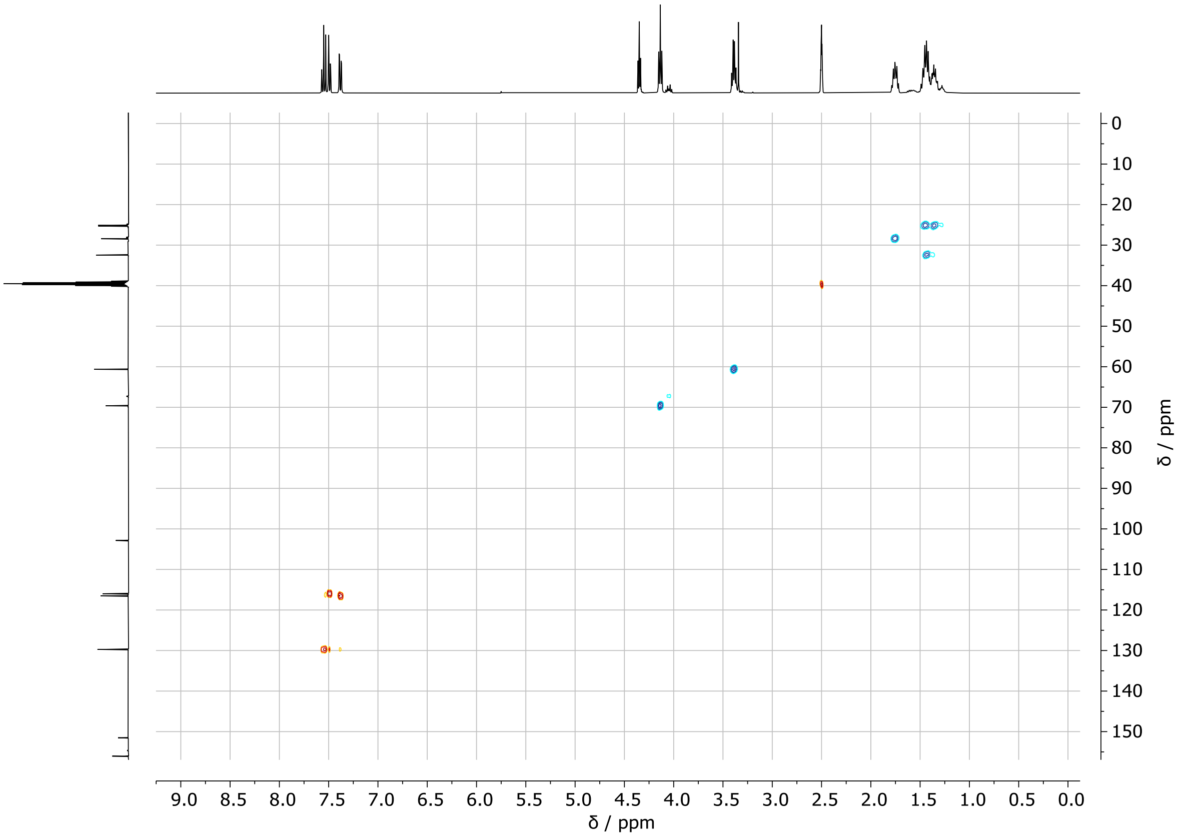

Figure S47. ^{1}H (400 MHz) - ^{13}C (101 MHz) HSQC spectrum of **4** in DMSO-d_6 at ambient temperature.

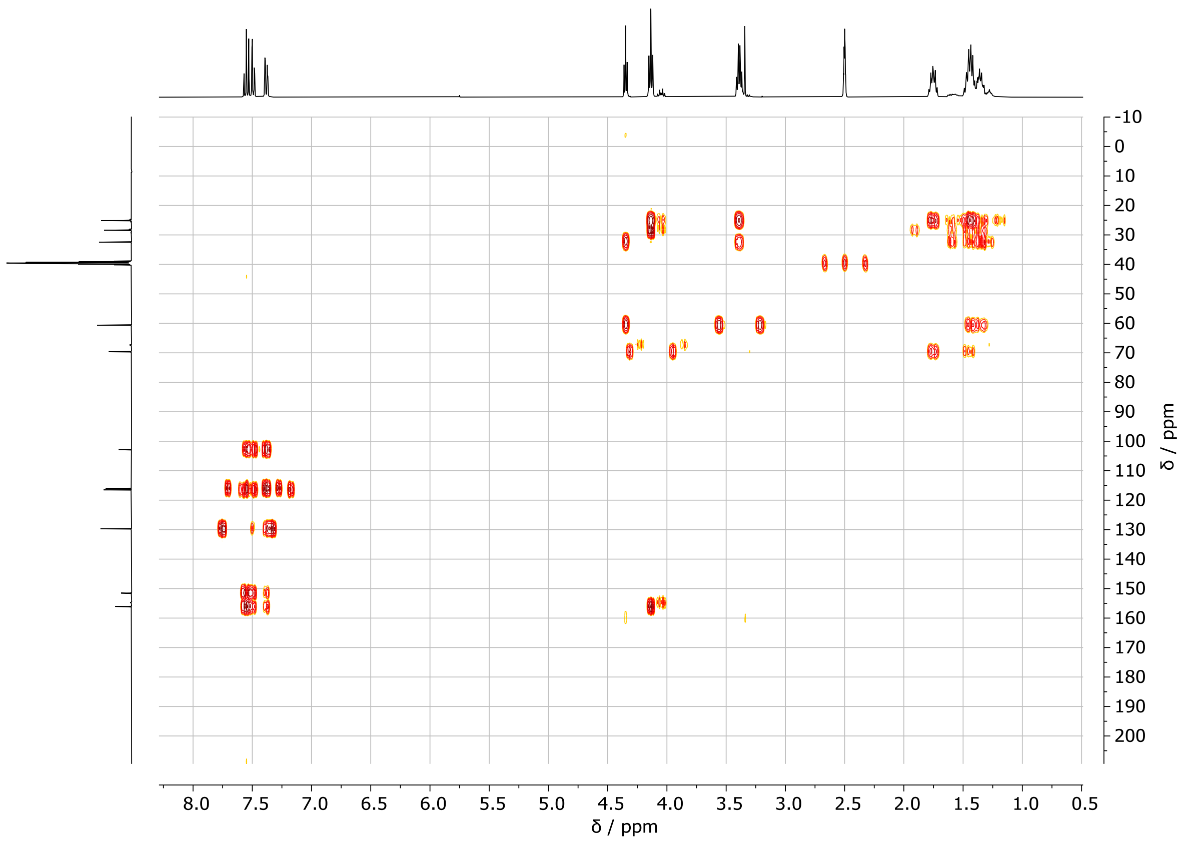

Figure S48. ^{1}H (400 MHz) - ^{13}C (101 MHz) HMBC spectrum of **4** in DMSO-d_6 at ambient temperature.

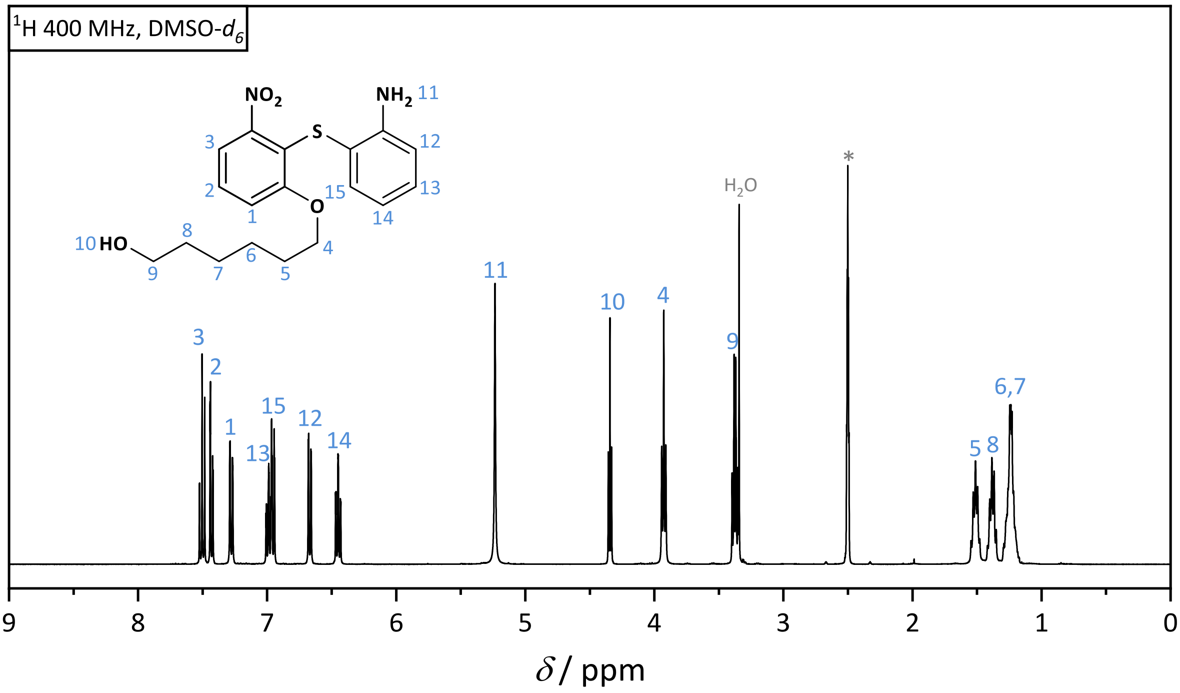

Figure S49. ^{1}H NMR (400 MHz) spectrum of **5** in DMSO-d_6 (*) at ambient temperature.

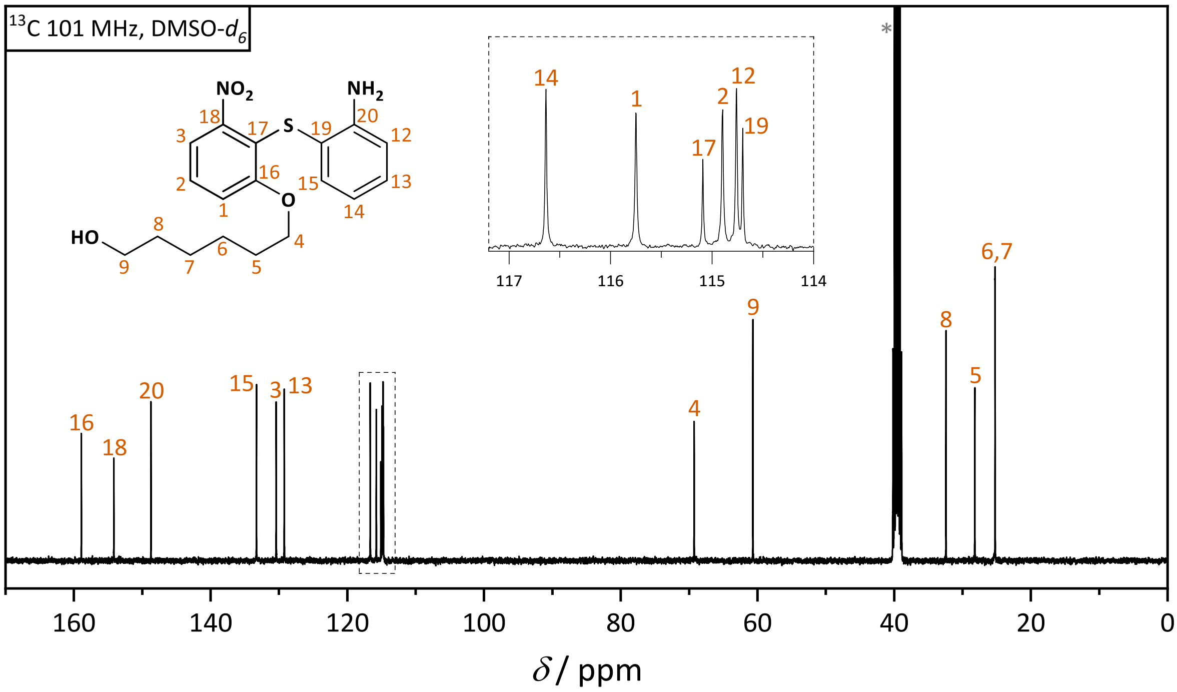

Figure S50. ^{13}C NMR (101 MHz) spectrum of **5** in DMSO-d_6 (*) at ambient temperature.

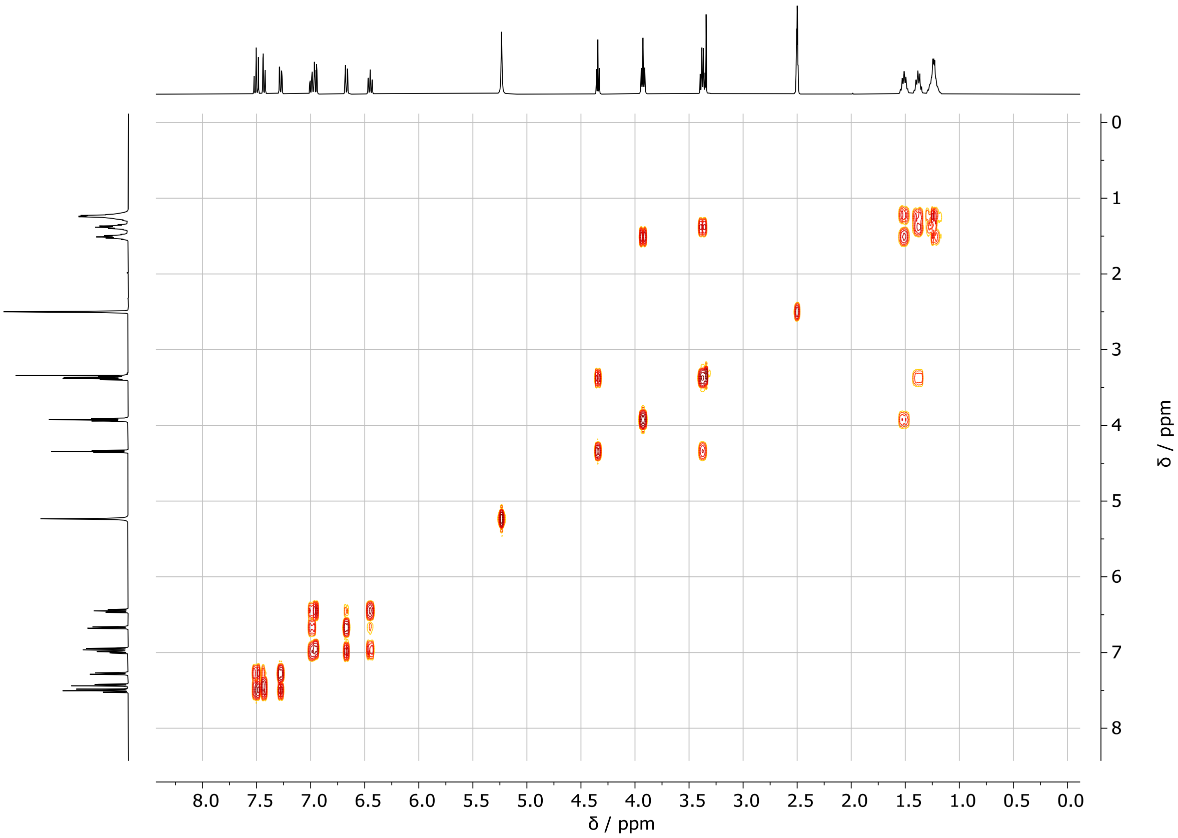

Figure S51. ^{1}H-^{1}H COSY (400 MHz) spectrum of **5** in DMSO-d_6 at ambient temperature.

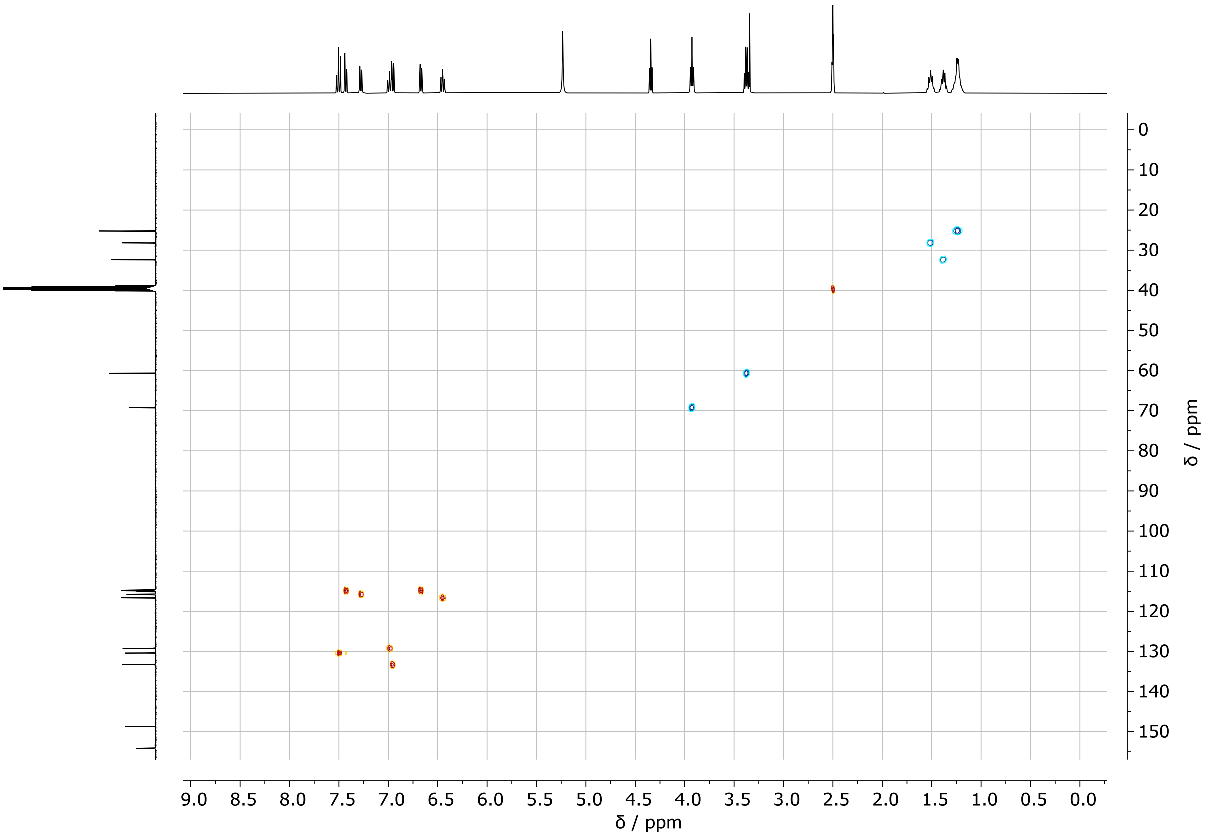

Figure S52. ^{1}H (400 MHz) - ^{13}C (101 MHz) HSQC spectrum of **5** in DMSO-d_6 at ambient temperature.

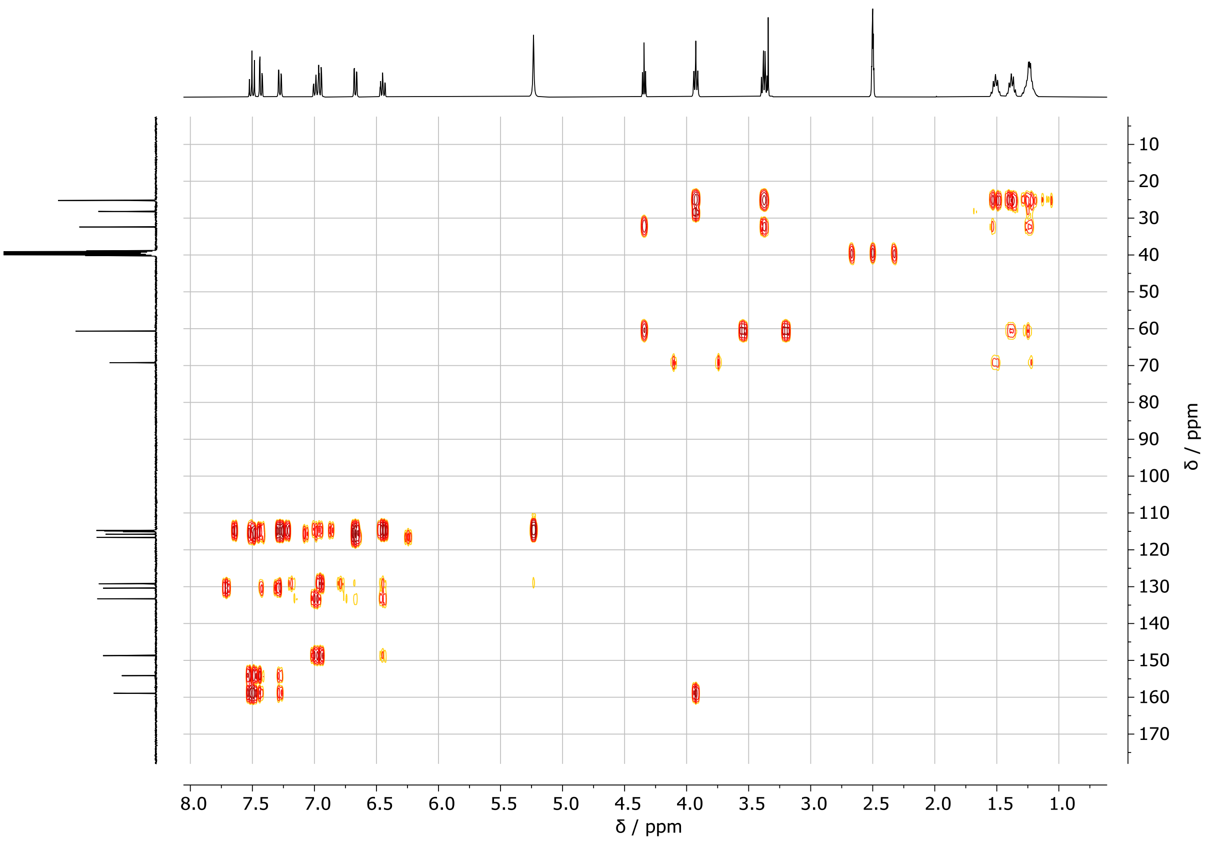

Figure S53. ^{1}H (400 MHz) - ^{13}C (101 MHz) HMBC spectrum of **5** in DMSO-d_6 at ambient temperature.

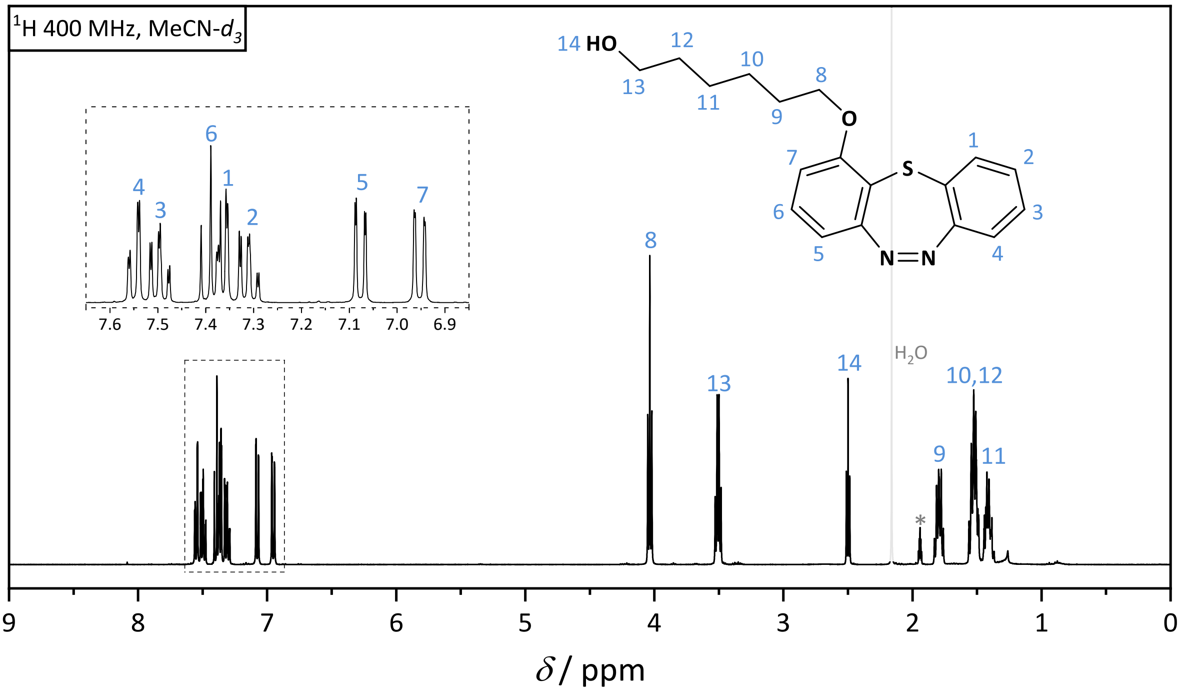

Figure S54. ^{1}H NMR (400 MHz) spectrum of SA-OH in MeCN-d_3 (*) at ambient temperature.

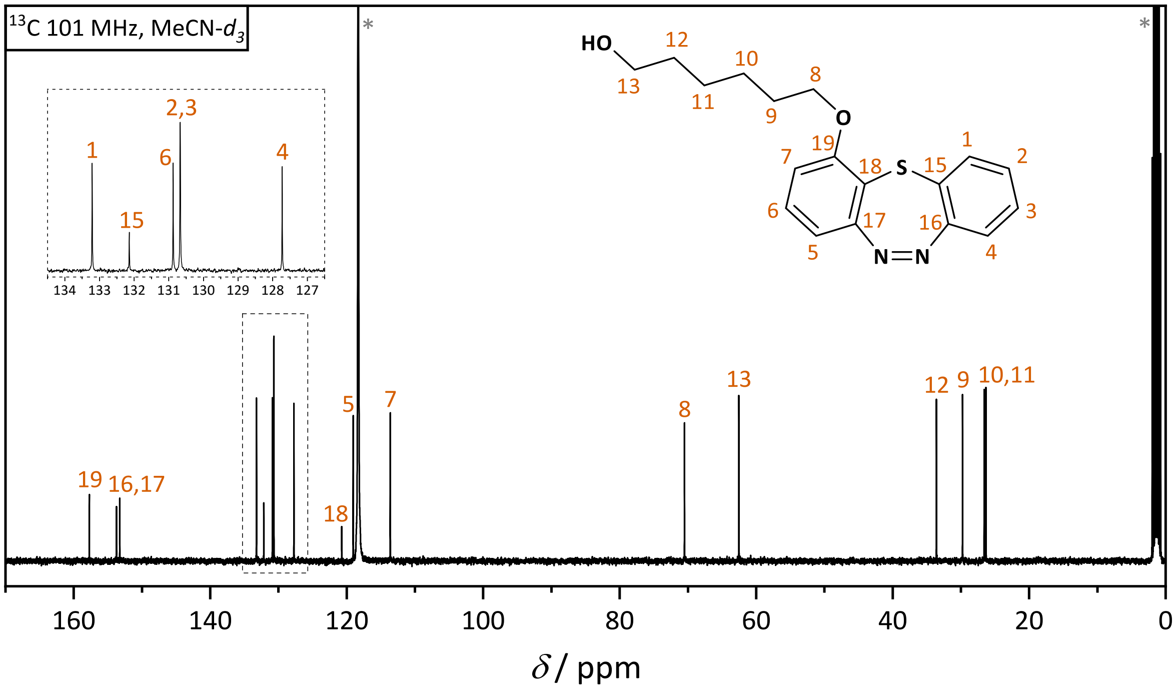

Figure S55. ^{13}C NMR (101 MHz) spectrum of SA-OH in MeCN-d_3 (*) at ambient temperature.

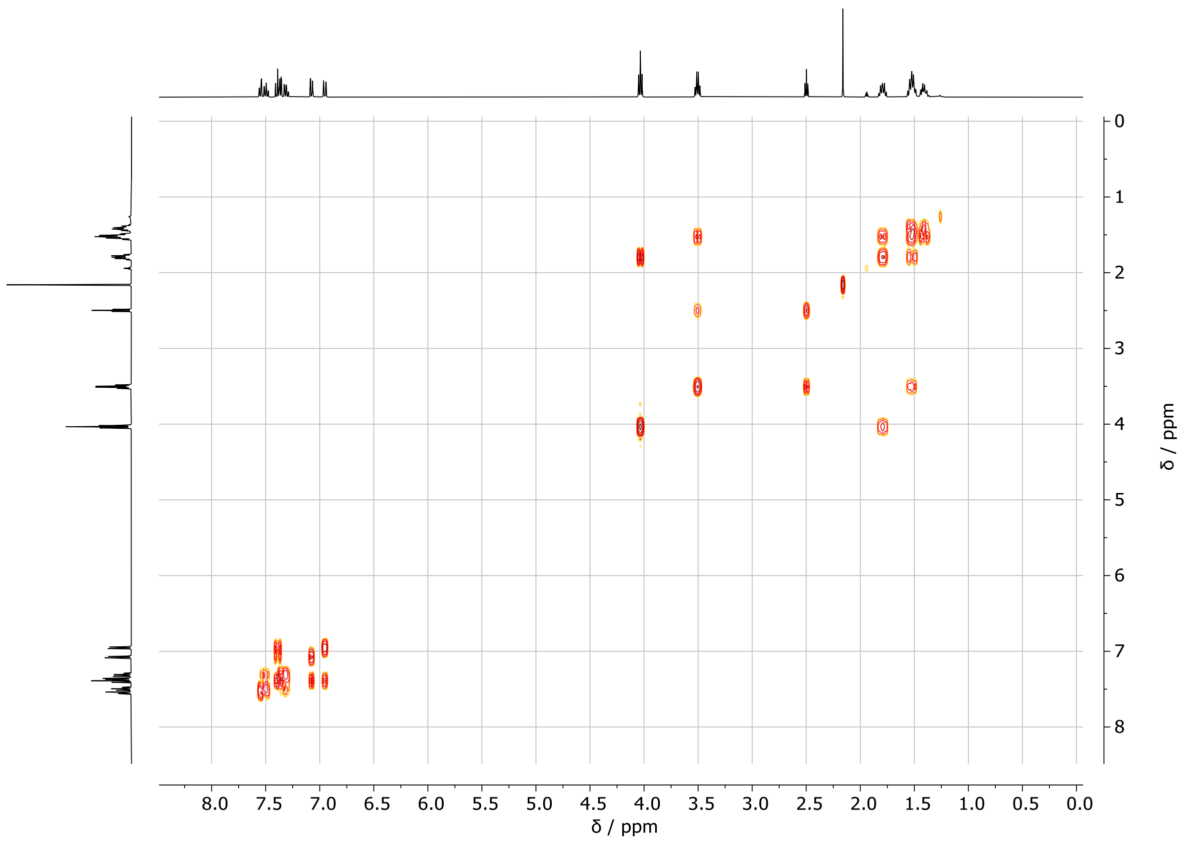

Figure S56. ^{1}H-^{1}H COSY (400 MHz) spectrum of SA-OH in MeCN-d_3 at ambient temperature.

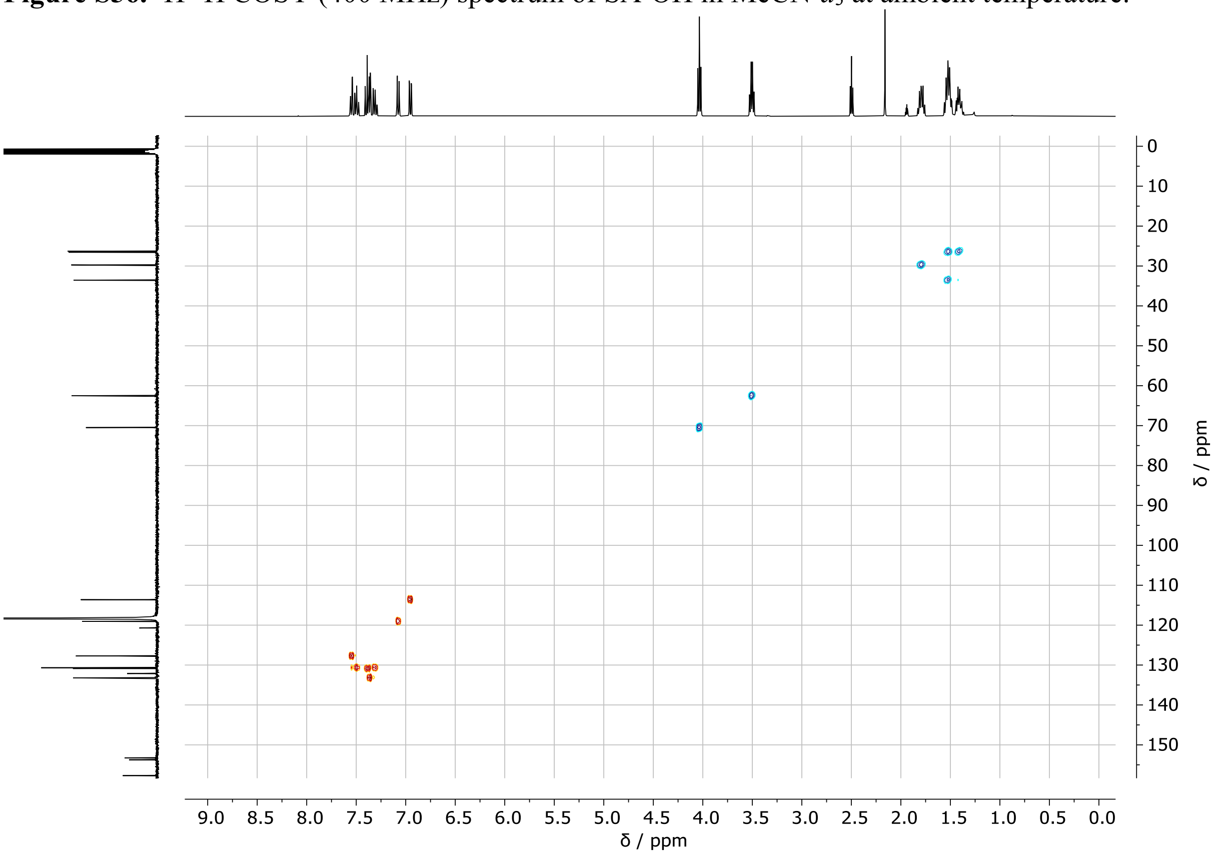

Figure S57. ^{1}H (400 MHz) - ^{13}C (101 MHz) HSQC spectrum of SA-OH in MeCN-d_3 at ambient temperature.

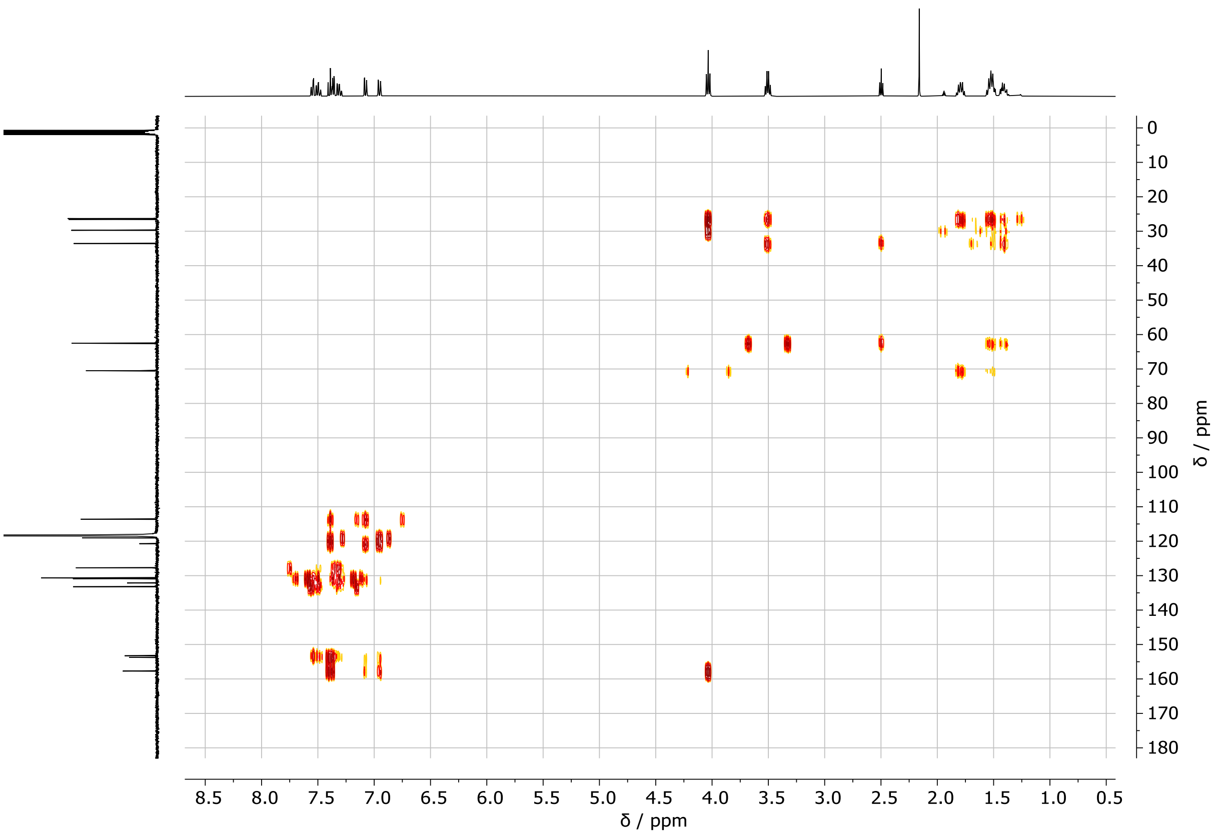

Figure S58. ^{1}H (400 MHz) - ^{13}C (101 MHz) HMBC spectrum of SA-OH in MeCN-d_3 at ambient temperature.

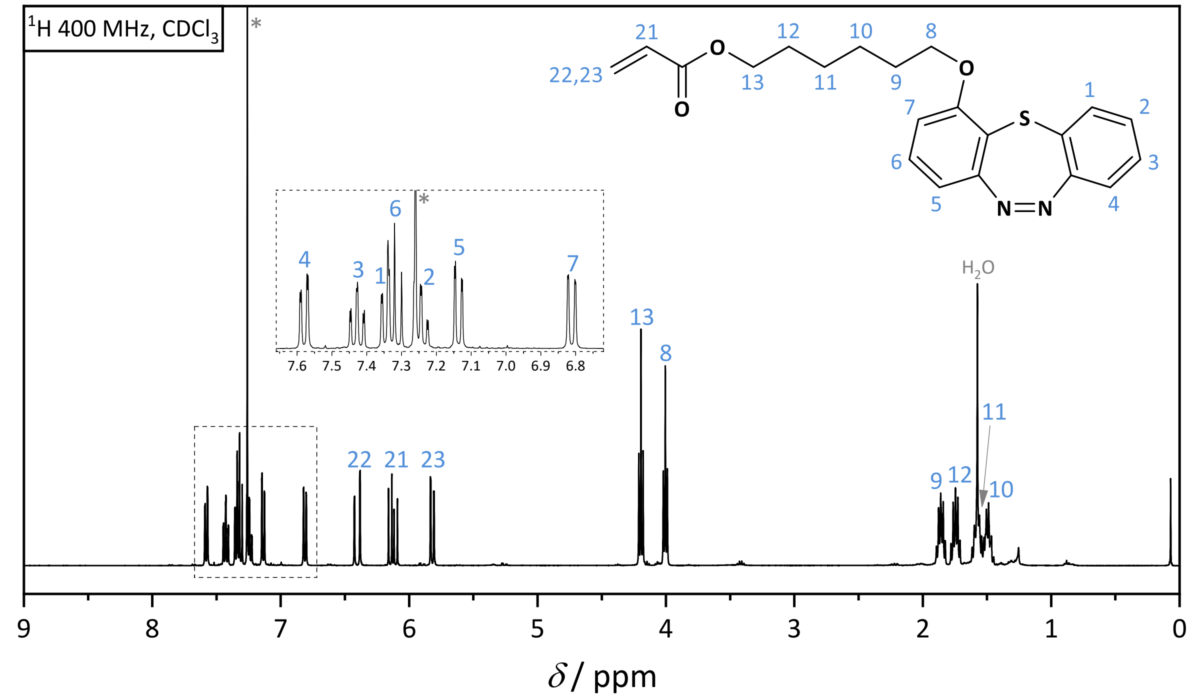

Figure S59. ^{1}H NMR (400 MHz) spectrum of **6** in $CDCl_3$ (*) at ambient temperature.

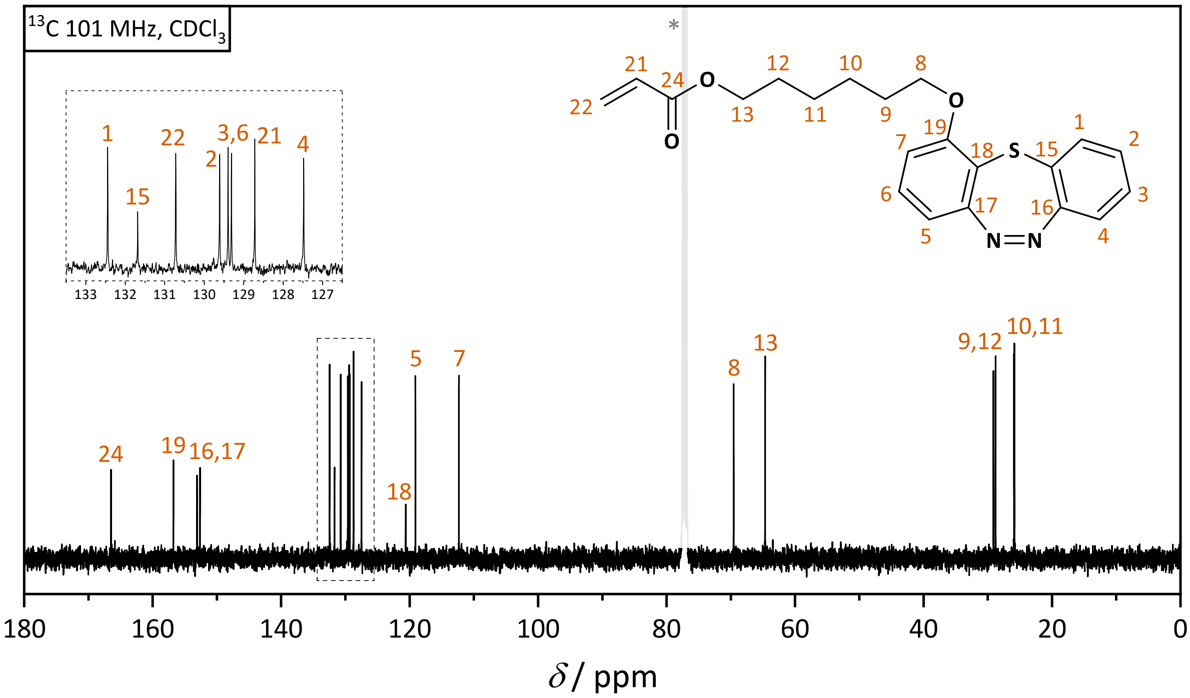

Figure S60. ^{13}C NMR (101 MHz) spectrum of **6** in $CDCl_3$ (*) at ambient temperature.

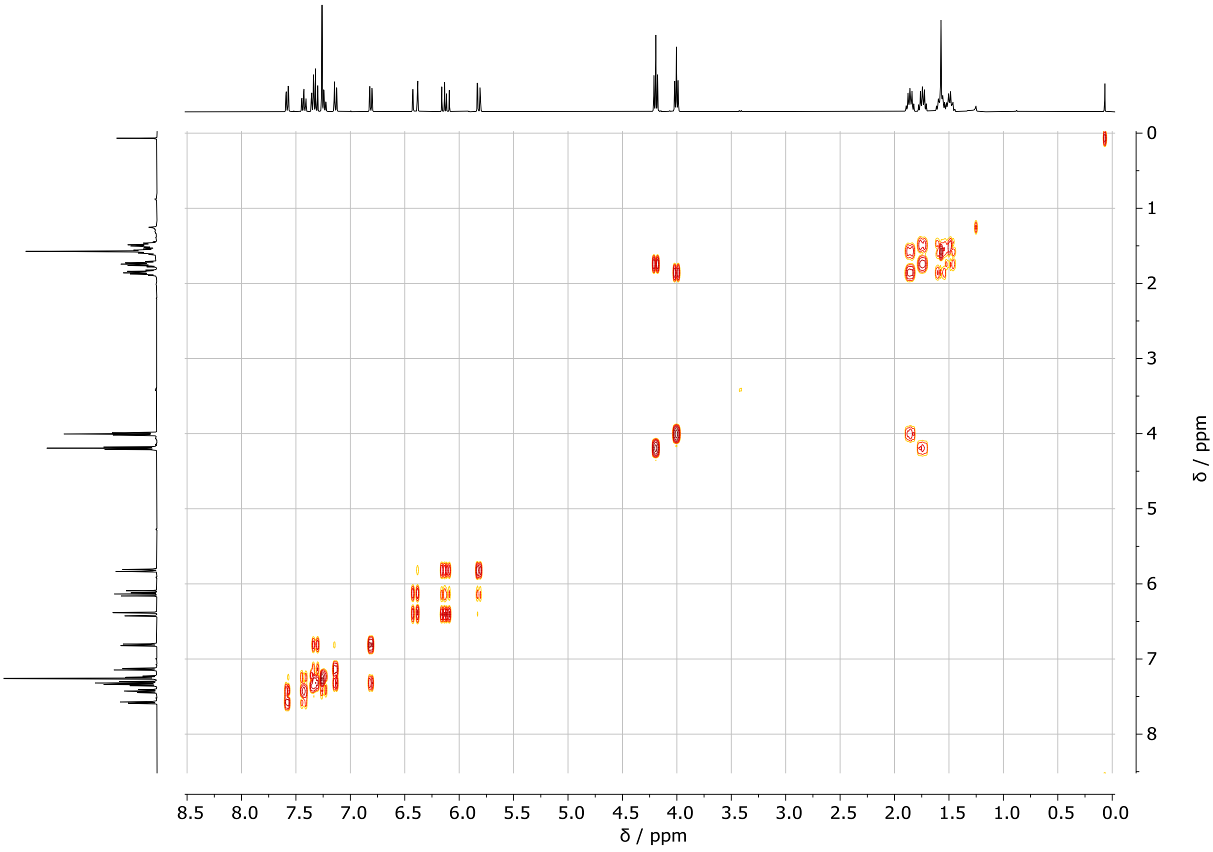

Figure S61. ^{1}H-^{1}H COSY (400 MHz) spectrum of **6** in $CDCl_3$ at ambient temperature.

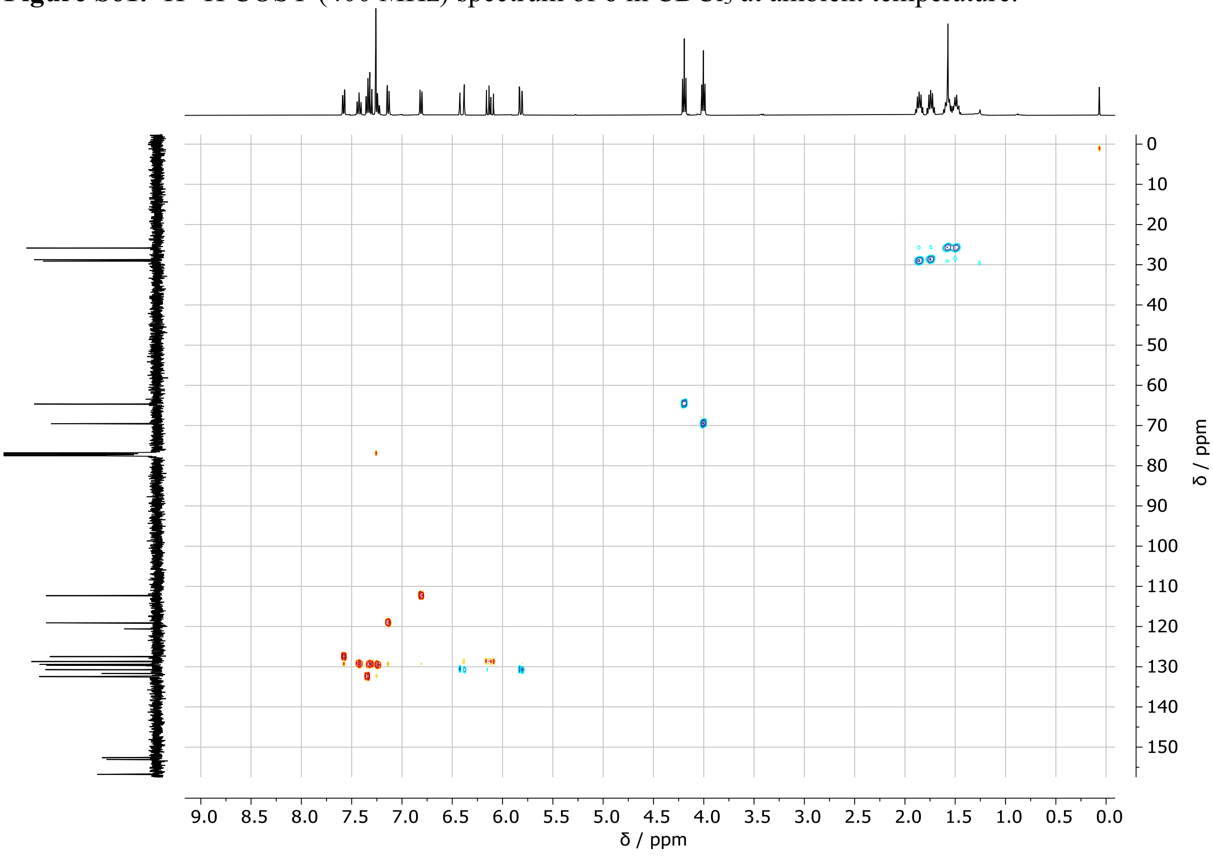

Figure S62. ^{1}H (400 MHz) - ^{13}C (101 MHz) HSQC spectrum of **6** in $CDCl_3$ at ambient temperature.

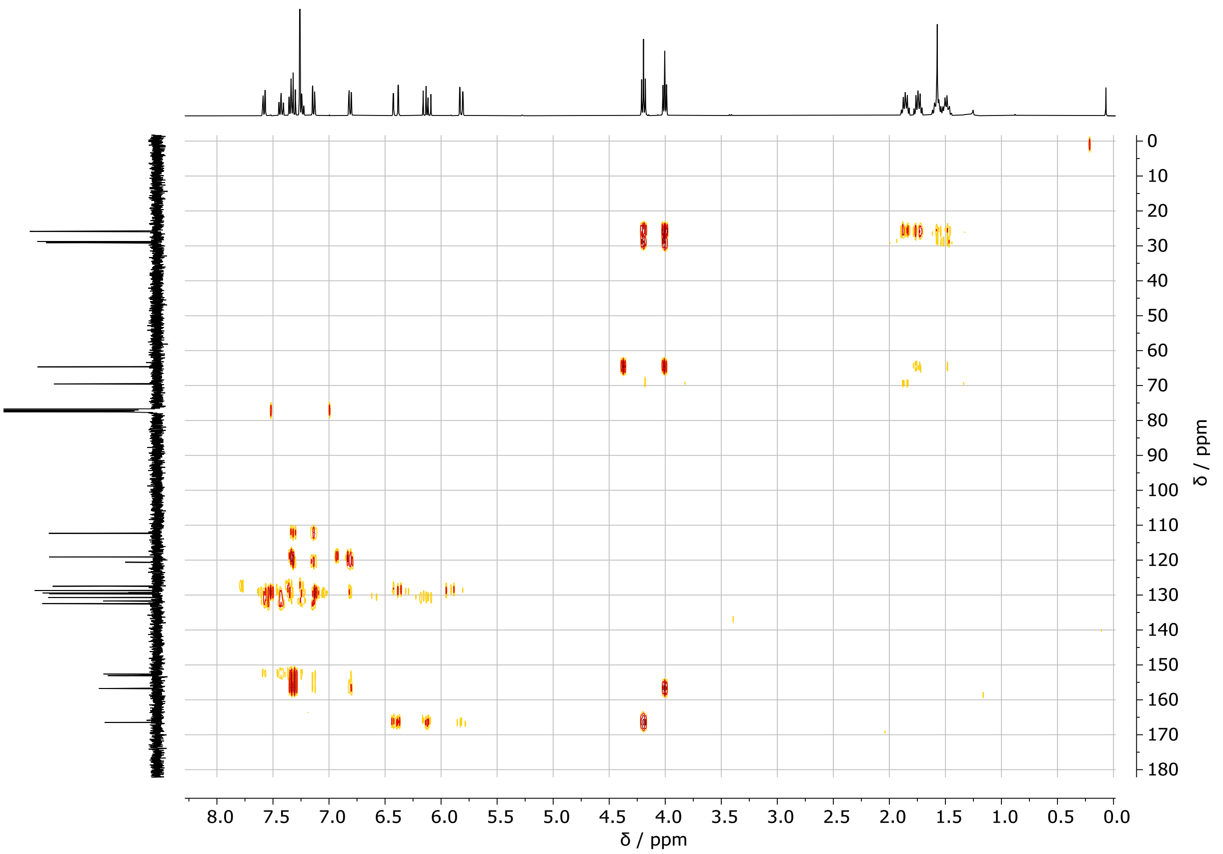

Figure S63. ^{1}H (400 MHz) - ^{13}C (101 MHz) HMBC spectrum of **6** in $CDCl_3$ at ambient temperature.

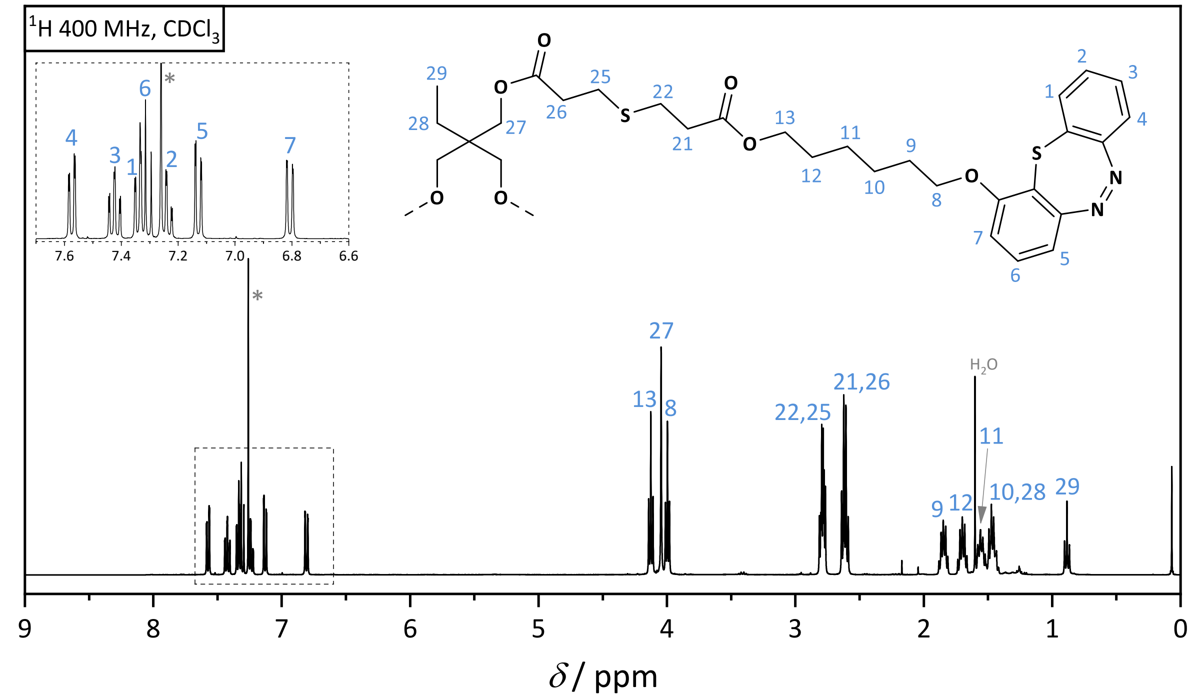

Figure S64. ^{1}H NMR (400 MHz) spectrum of SA-3arm in $CDCl_3$ (*) at ambient temperature.

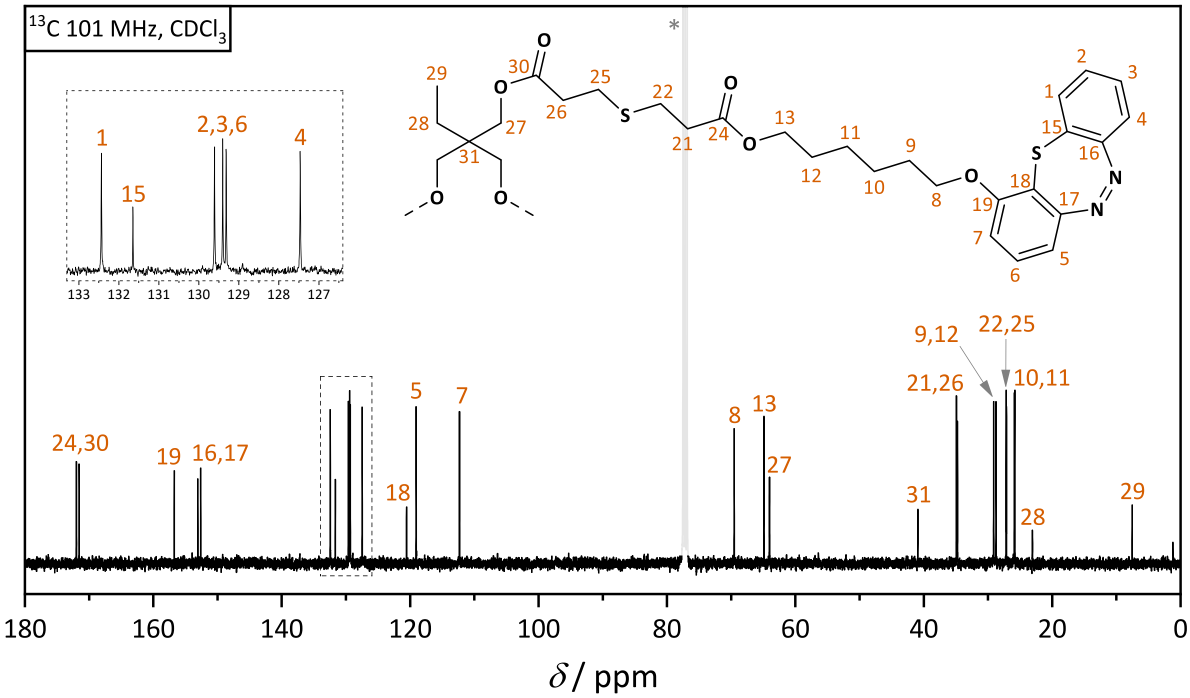

Figure S65. ^{13}C NMR (101 MHz) spectrum of SA-3arm in $CDCl_3$ (*) at ambient temperature.

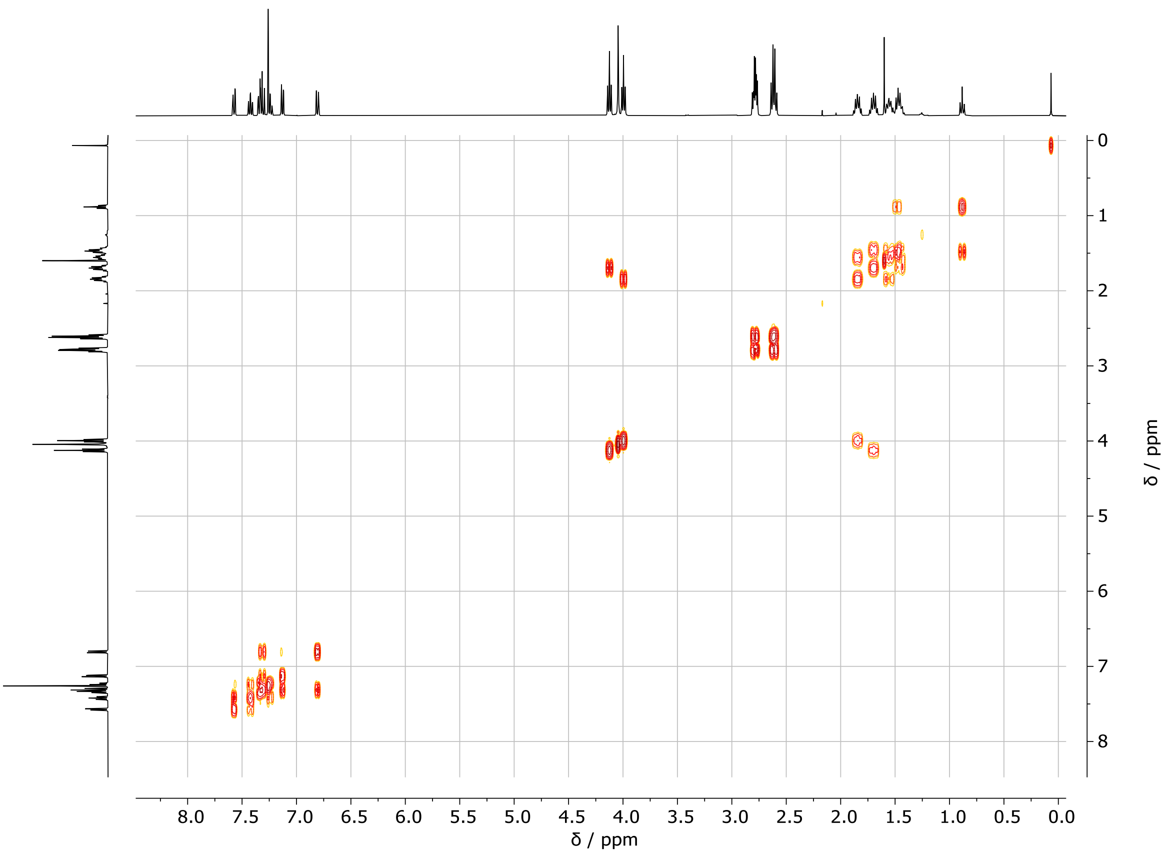

Figure S66. ^{1}H-^{1}H COSY (400 MHz) spectrum of SA-3arm in $CDCl_3$ at ambient temperature.

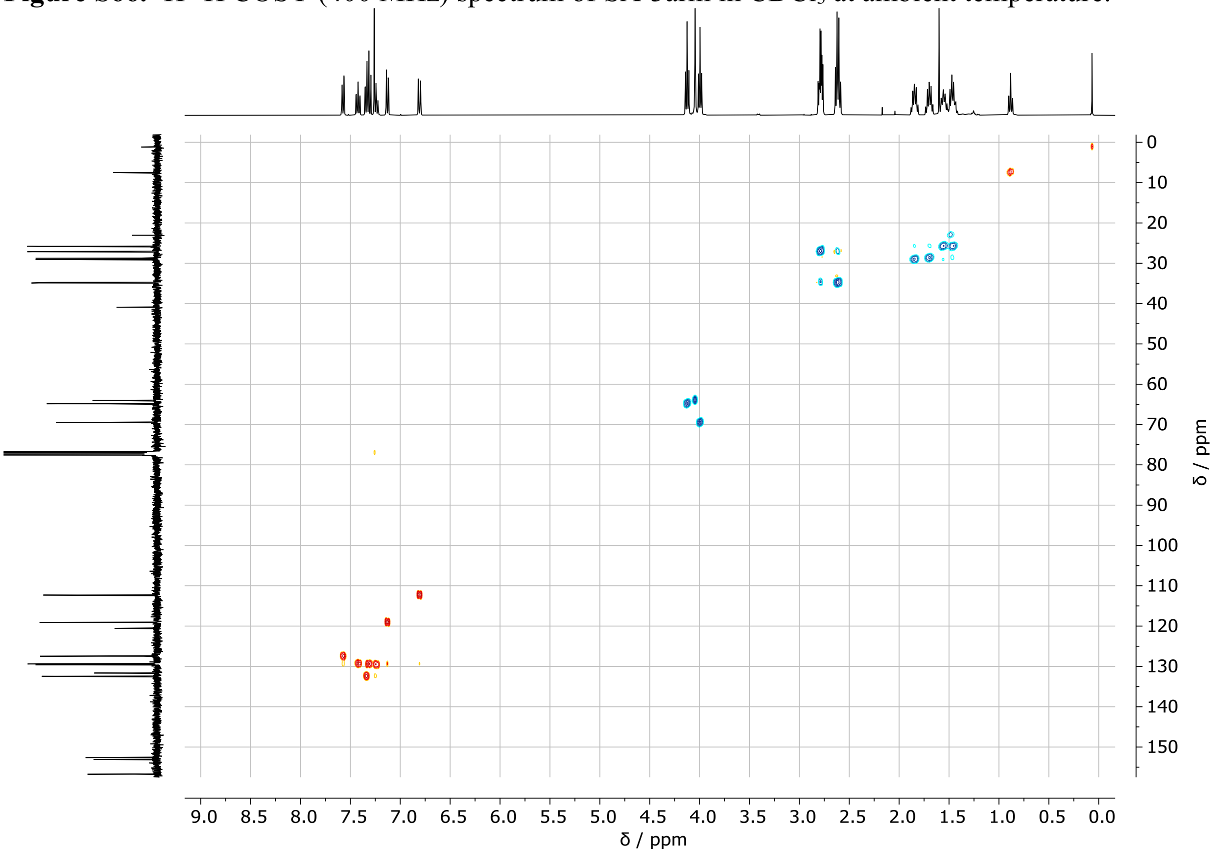

Figure S67. ^{1}H (400 MHz) - ^{13}C (101 MHz) HSQC spectrum of SA-3arm in $CDCl_3$ at ambient temperature.

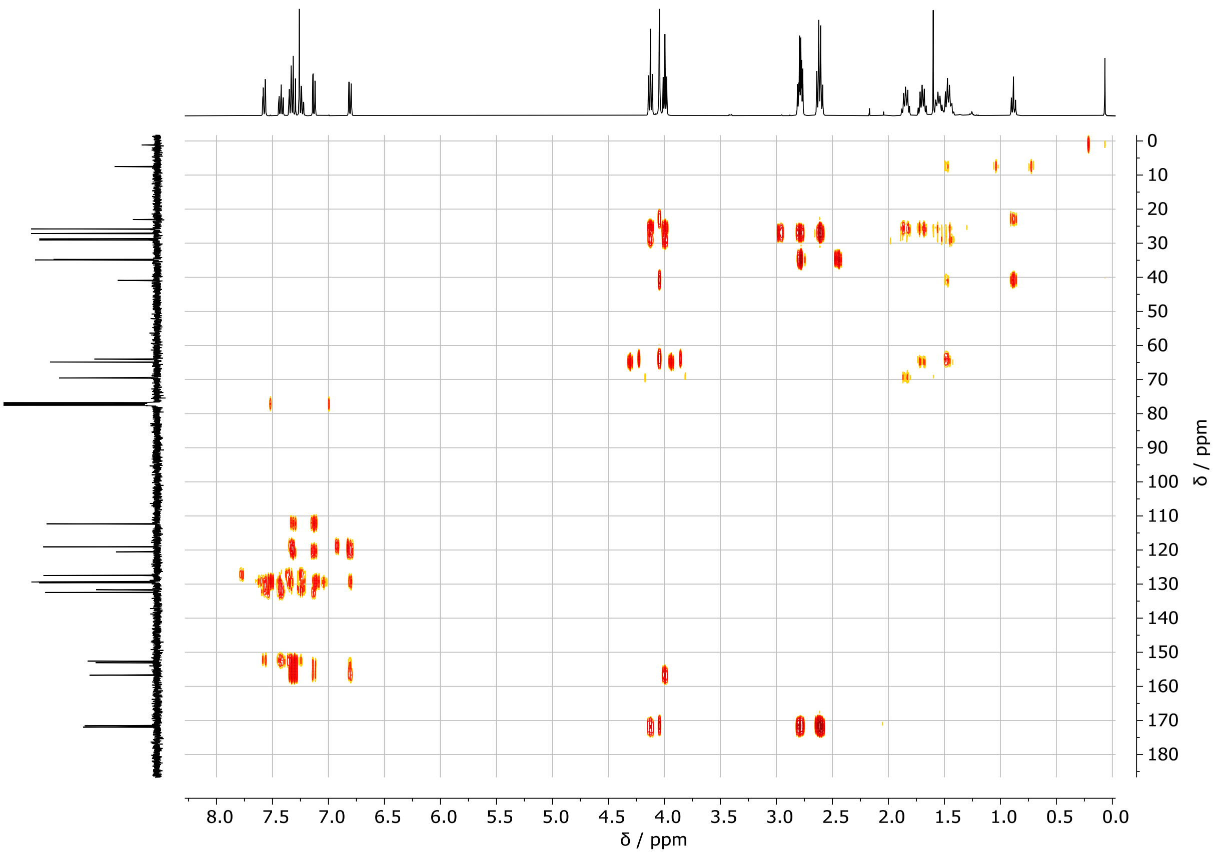

Figure S68. ^{1}H (400 MHz) - ^{13}C (101 MHz) HMBC spectrum of SA-3arm in $CDCl_3$ at ambient temperature.

8 LC-MS Measurements

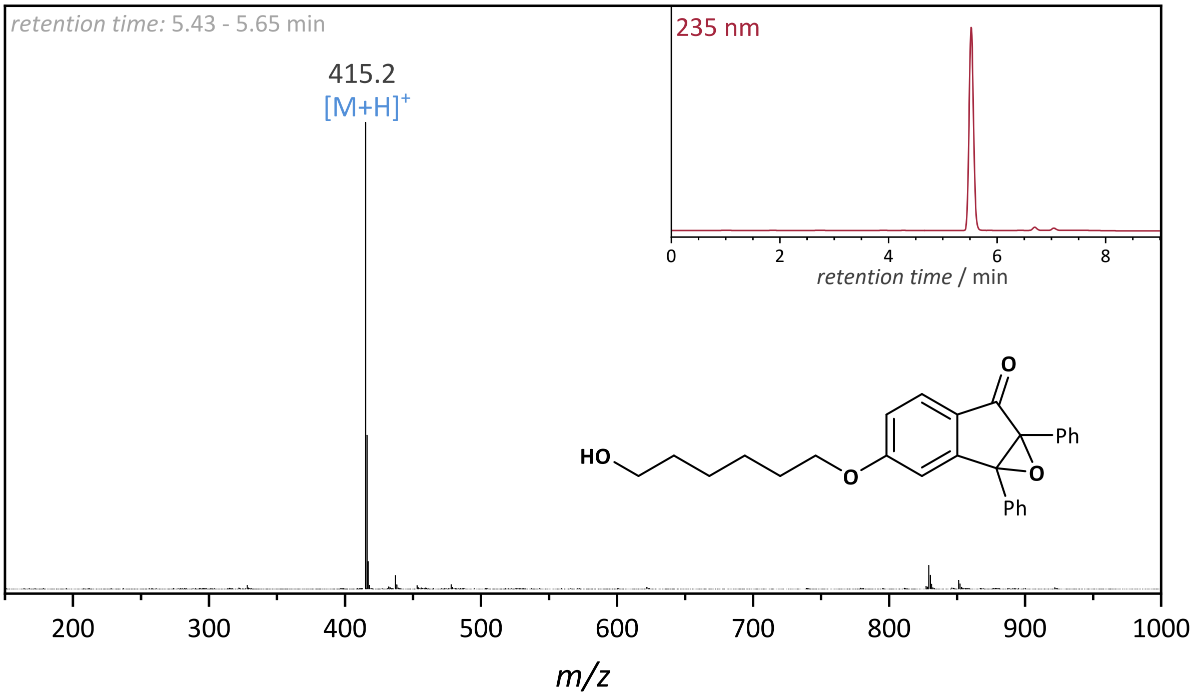

Figure S69. LC-trace (235 nm detector wavelength) and accumulated mass-spectra of DIO-OH.

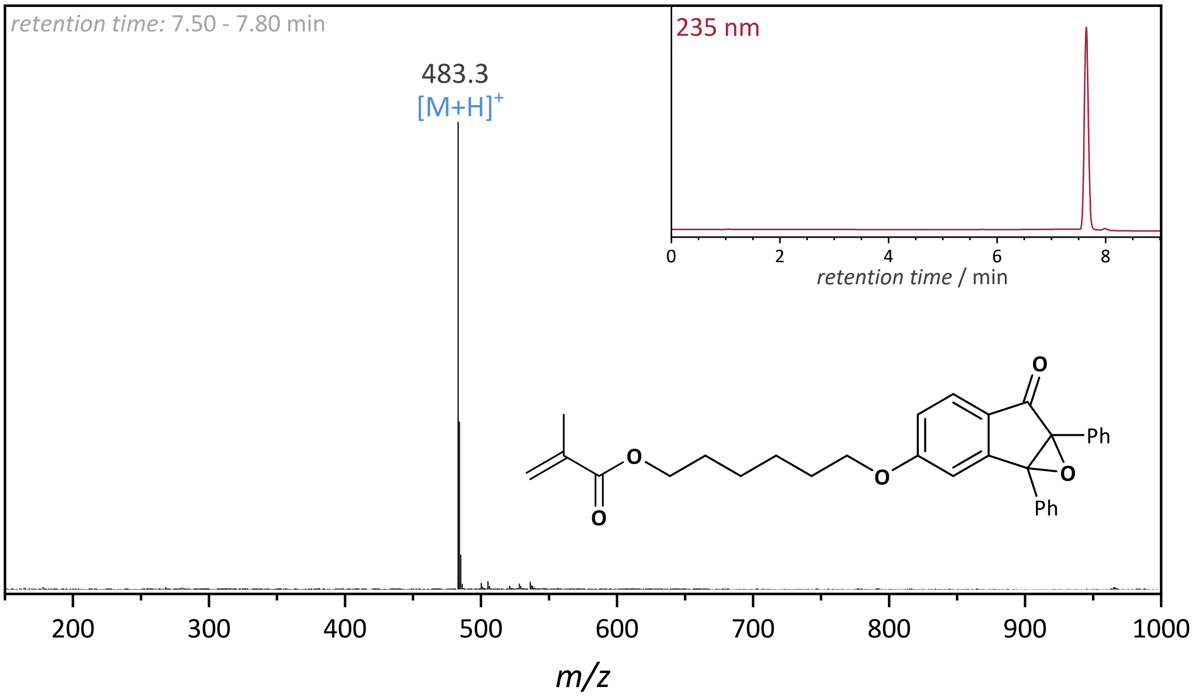

Figure S70. LC-trace (235 nm detector wavelength) and accumulated mass-spectra of **3**.

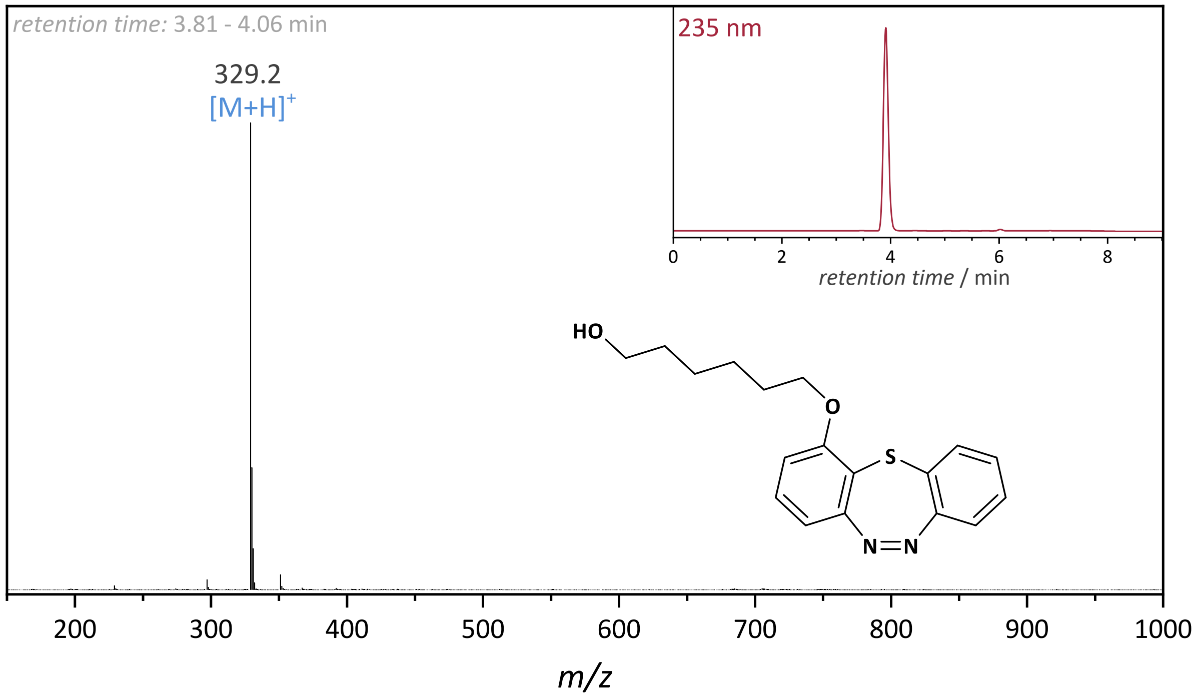

Figure S71. LC-trace (235 nm detector wavelength) and accumulated mass-spectra of SA-OH.

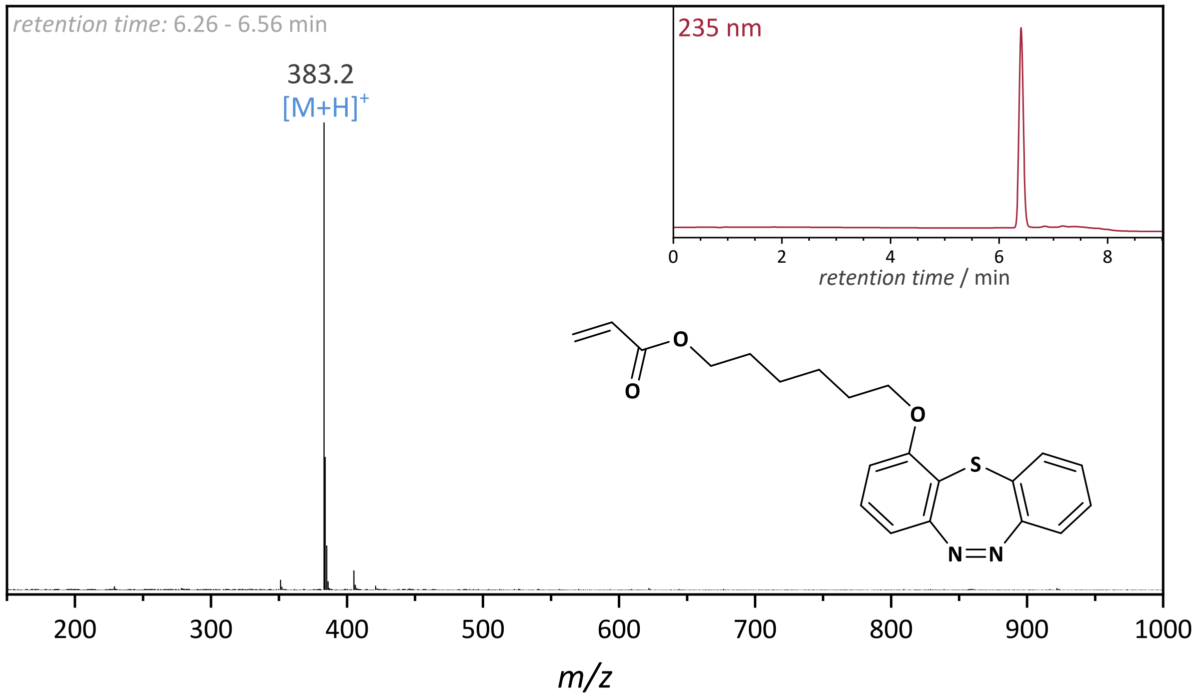

Figure S72. LC-trace (235 nm detector wavelength) and accumulated mass-spectra of **6**.

9 UV-Vis Spectra

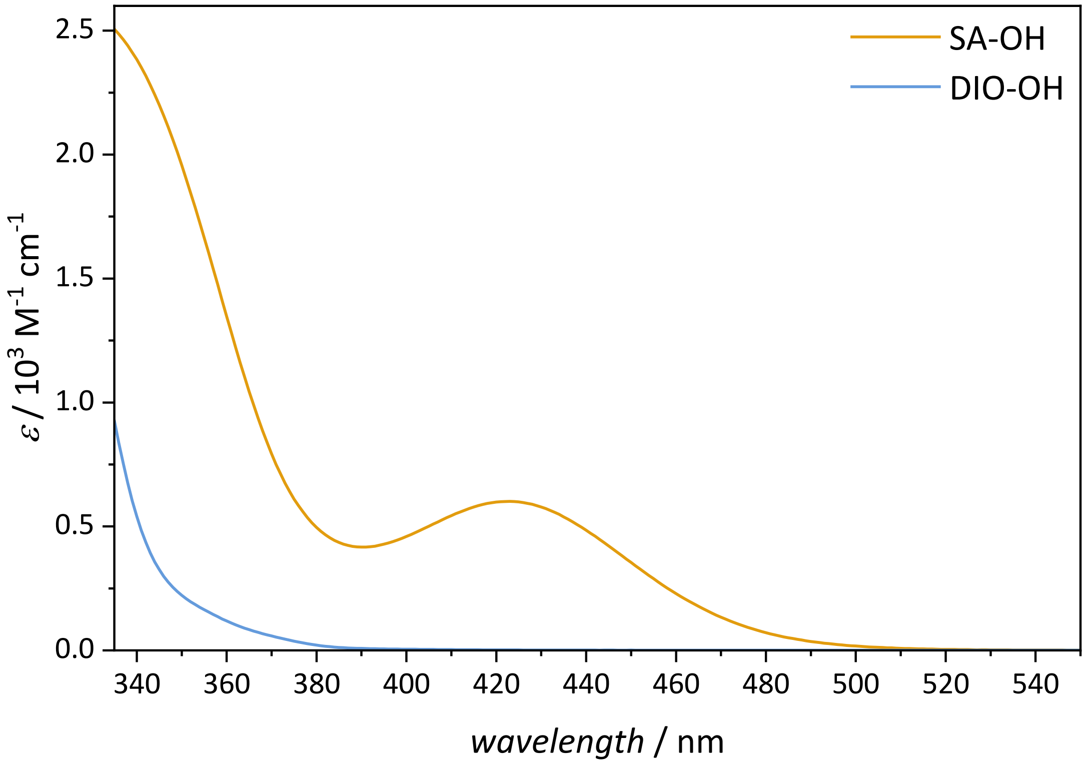

Figure S73. UV-Vis absorption spectra of DIO-OH (blue) and SA-OH (yellow) in acetonitrile.

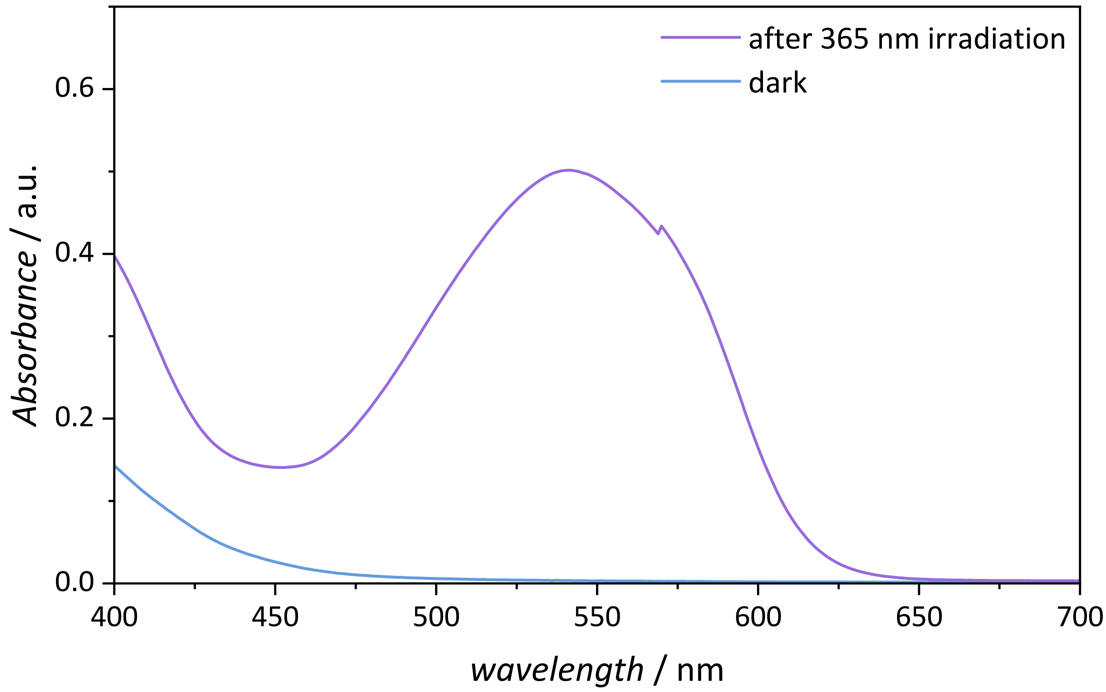

Figure S74. UV-Vis absorption spectra of polyDIO (10 mg mL^{-1}) before (blue) and after irradiation with light at 365 nm (pink) in acetophenone, evincing photoswitching.

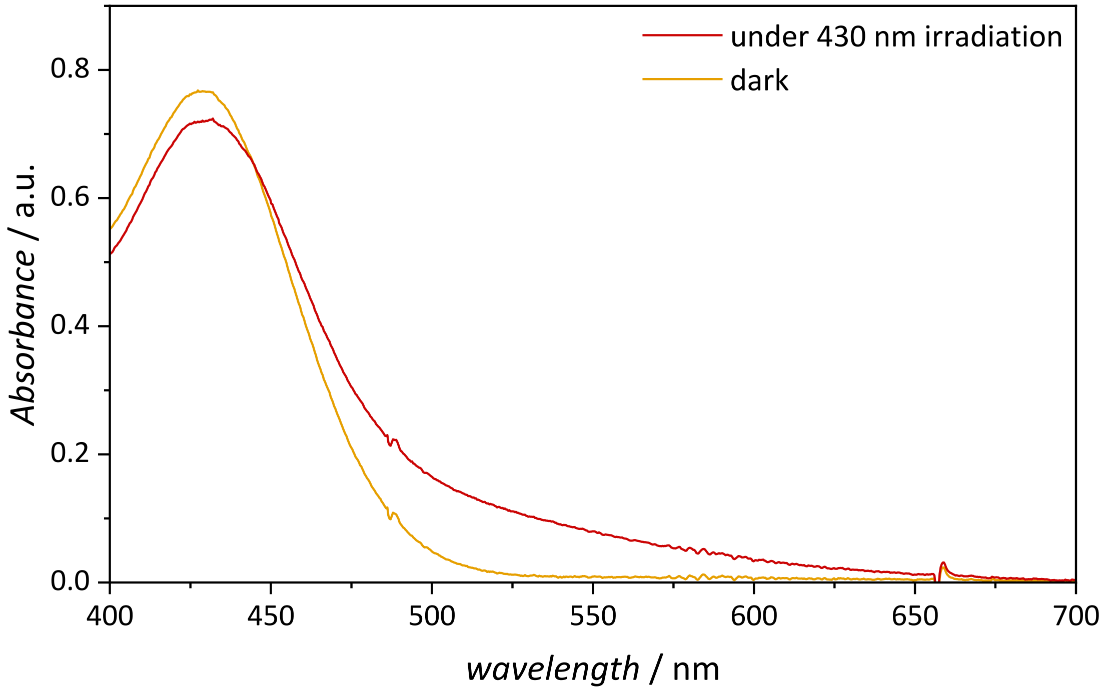

Figure S75. UV-Vis absorption spectra of SA-3arm before (yellow) and under irradiation with light at 430 nm (red) in acetophenone, evincing photoswitching.

10 ATR-IR Spectra

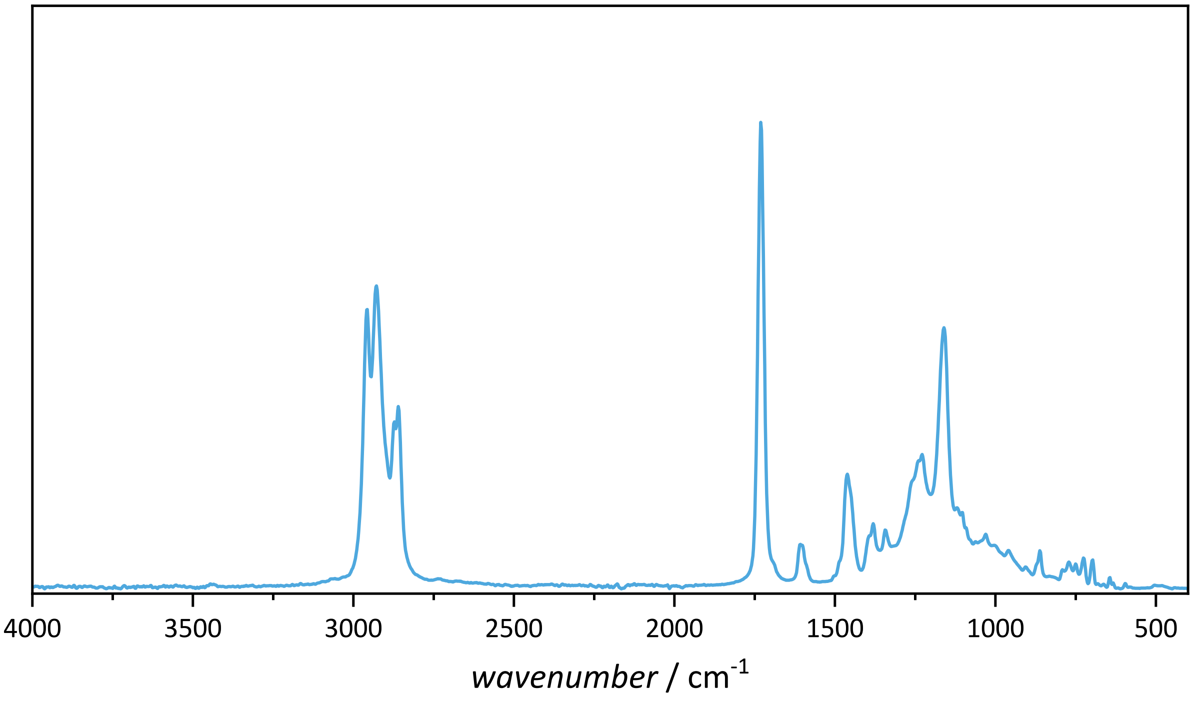

Figure S76. ATR-IR spectrum of polyDIO in the range of 400 to 4000 cm^{-1}.

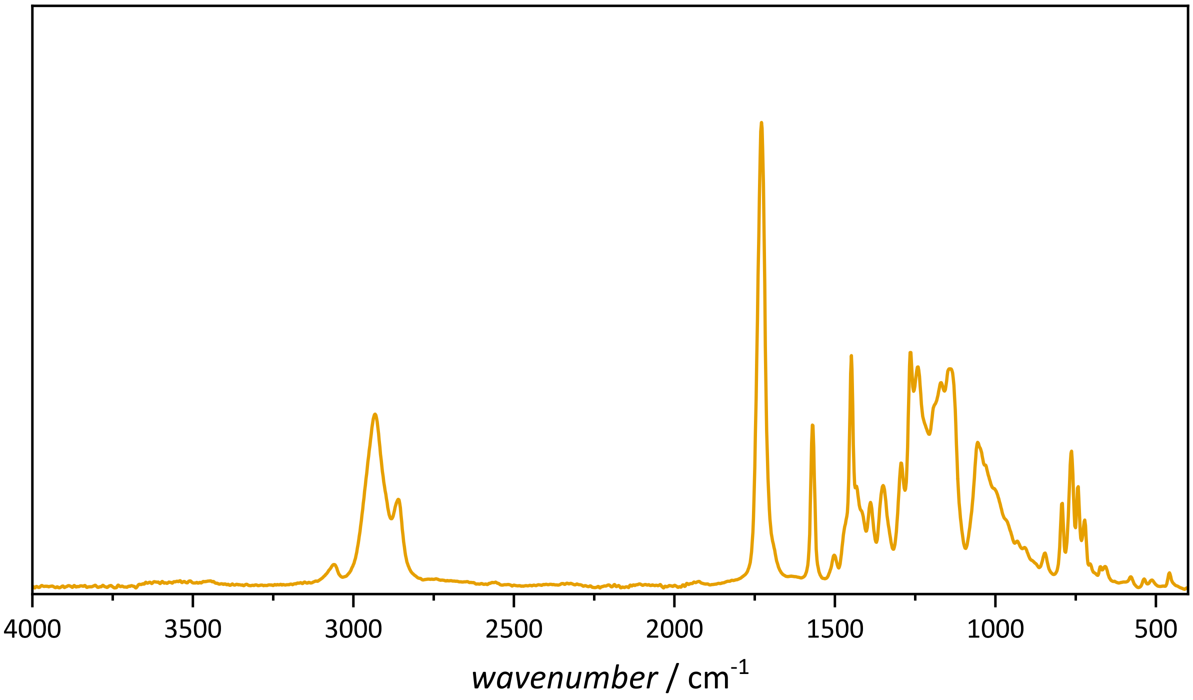

Figure S77. ATR-IR spectrum of SA-3arm in the range of 400 to 4000 cm^{-1}.

11 Thermal Characterization

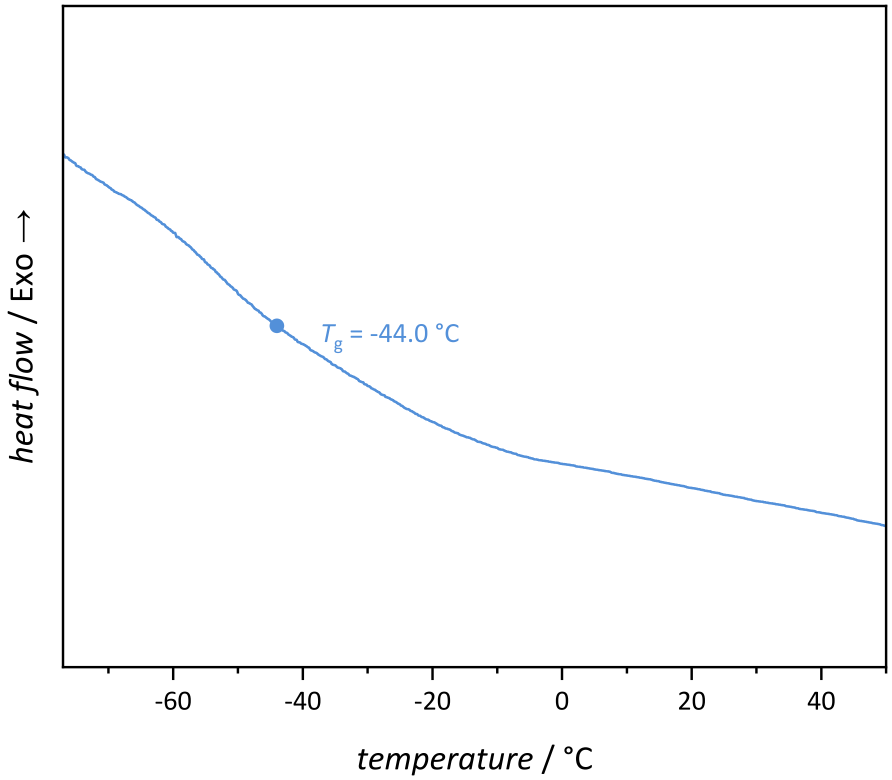

Figure S78. DSC curve of polyDIO from -90 to 100 °C (second heating run) with a heating rate of 20 K min^{-1}.

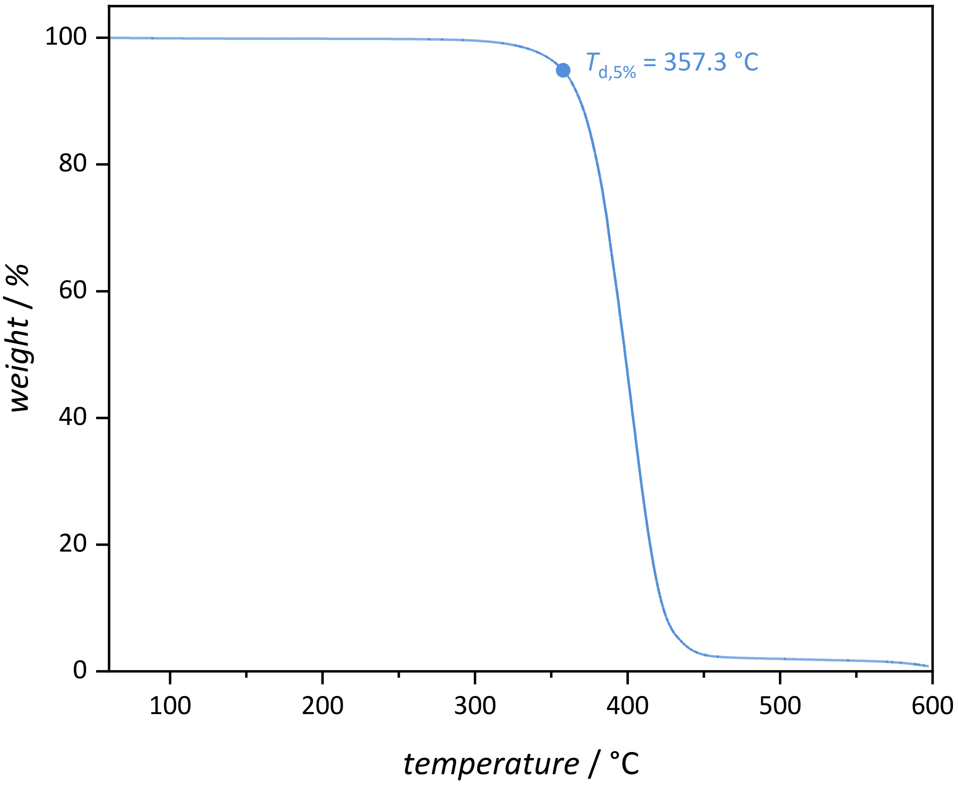

Figure S79. TGA curve of polyDIO from 25 to 600 °C with a heating rate of 10 K min^{-1}.

12 Two-color Lithography

12.1 Setup

All components of the two-color lithography setup are mounted on an optical table. As shown in **Scheme S1**, we incorporated a continuous-wave (CW) 375 nm laser into our custom-designed Mono LISA 3D printer for two-color lithography. An *Innolas* SpitLight 600 optical parametric oscillator (OPO) wavelength-tunable laser producing 7 ns, 100 Hz pulses serves as 430 nm light source and TOPTICA IBEAM-SMART-PT 375 nm serves as 375 nm light source. The output beam of the pulsed laser passes through a Dove prism (Pr) and subsequently through two cylindrical lenses (L1 and L2, f = 250.0 mm and -100.0 mm, respectively) and an attenuator (A, used to adjust the pulsed laser power). It then passes spherical lenses (L3 and L4, f = 50.0 mm and 150.0 mm respectively) and mirrors (F and M1). The flip mirror (F) is employed to direct the pulsed laser beam in the UV region along its designed pathway. To allow the 430 nm beam to pass, F must be flipped down. The pulsed beam is then combined with the CW 375 nm laser via a beam splitter (B, 50:50). The combined beam further passes two spherical lenses (L3 and L5, f = 50.0 mm and 75.0 mm, respectively), a mirror (M1) and a shutter (S), before being directed to the Galvo mirror (dual-axis, 250 µs step response time). After passing two lenses (L6, f = 150.0 mm), the beam is focused on a z-stage (Thorlabs motorized translation stage with a KSC101 - K-Cube Solenoid Controller) via a 45° mirror (M2) and a focusing lens (L7, f = 75.0 mm). The power meter (P) is positioned between L6 and M2 to measure all powers for printing, ensuring both convenience and safety. The movement of the Galvo mirror (G) and the shutter (S, on-off) is controlled by our customized software, programmed in LabView. Upon importing G-code files containing the coordinates and travel speed, the software processes the files to precisely control both the printing trajectory and speed. The resolution of the laser printer is approximately 20 µm, as determined by the focused beam size.

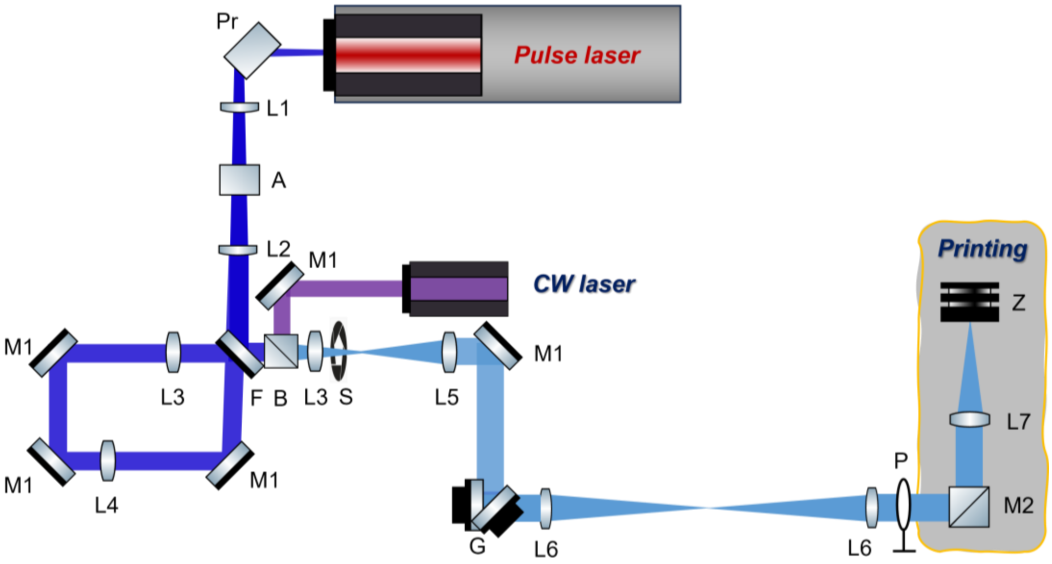

Scheme S1. Schematic of the two-color lithography setup, showing the beam path and various components.

12.2 Sample preparation and lithography

The ink for 2D printing was prepared by mixing polyDIO (720 mg) with SA-3arm (235 mg) in acetophenone (2.146 mL, 2.210 g). After placing the glass sample holder onto the Z stage, the ink was directly deposited onto glass slide for printing. The G-code files for lines, rings and butterflies were generated with UltiMaker Cura (v. 5.6.0). The beam – by passing through the spherical lens (L7) – was focused onto the surface of the glass substrate. The objects were subsequently printed by controlling the motions of the Galvo mirror (G) with the software.

12.3 Line testing

To determine the fabrication windows, lines were printed at fixed laser powers at 375 nm and 430 nm, with varying printing speeds. Fabrication windows at 1, 2.5, 5, and 15 mW (375 nm), with or without additional 430 nm irradiation (10 mW), were evaluated, as shown in **Table S1**.

Table S1. Fabrication windows of lines printed with one- or two-color irradiation. 1C stands for one-color irradiation (375 nm) and 2C stands for two-color irradiation (375 nm + 430 nm, 430 nm at 10 mW). Color code: green – printable; grey – not printable.

	P (mW)	v (mm s^{-1})	0.1	0.13	0.16	0.2	0.23	0.26	0.3	0.33	0.36	0.4	0.45	0.5	0.55	0.6	0.65	0.7	0.75	0.8
1C	15																			
2C	15	10																		
1C	5																			
2C	5	10																		
1C	2.5																			
2C	2.5	10																		
1C	1																			
2C	1	10																		

Our previous study demonstrated enhanced synergistic effects at higher 430 nm laser powers.[3] Inspired by these findings, the effect of the laser power at 430 nm was investigated while keeping the 375 nm laser power fixed at 5 mW and the printing speed constant at 0.55 mm s^{-1} (**Figure S80**). According to the fabrication windows shown in Table S1, lines cannot be printed using 375 nm alone at 5 mW at a printing speed of 0.55 mm s^{-1}.

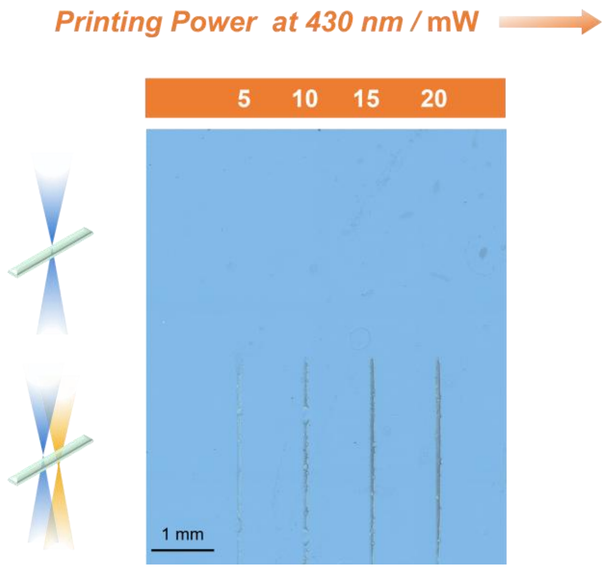

Figure S80. Optical microscopy images of lines printed with one- or two-color exposure. Top lines were printed with one color (375 nm, 5 mW, 0.55 mm s^{-1}), while bottom lines were printed using two colors with varying laser powers at 430 nm (375 nm at 5 mW, 0.55 mm s^{-1}) as illustrated by the schematic on the left.

12.4 Intricate structures

A series of ring patterns were designed with varying segments (100%, 75%, 50%, 25%, and 0%) printed using only 375 nm, while the remaining portions were printed using two-color exposure (375 nm + 430 nm) under identical 375 nm conditions.

The G-code files for the series of ring patterns are provided in **Supplementary Files 1**. **Figure S81** illustrates the trajectories and coordinates defined in the G-code for each structure. **Figure S82** shows the two-color lithography pathways by fabricating a shared border between the one-color and two-color regions.

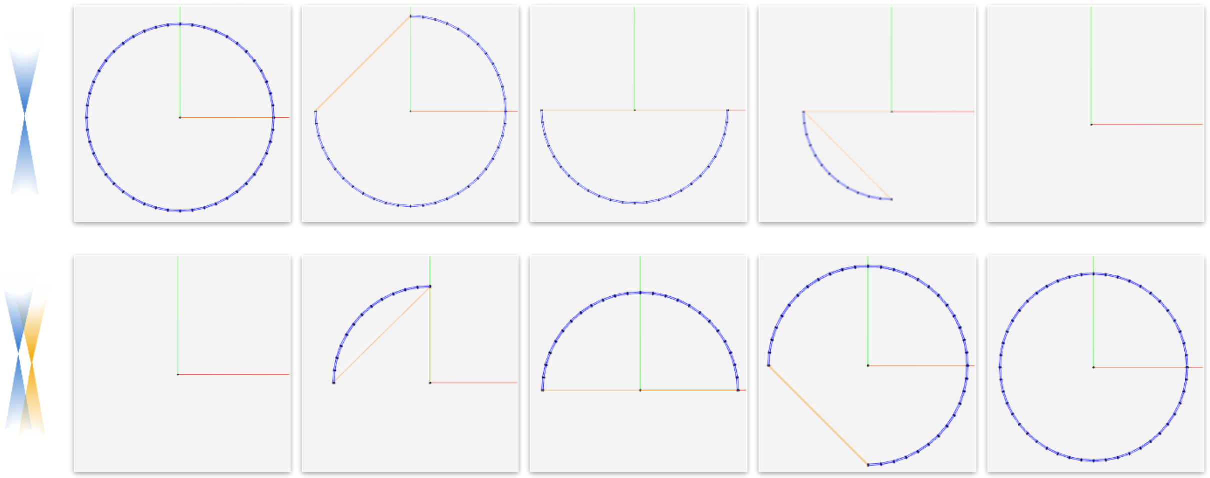

Figure S81. Images illustrating the trajectory of the 2 axes for the series of rings, generated from the G-code files to obtain the corresponding structures. The blue lines represent the trajectory when the shutter is on (i.e., resin exposure to the laser) with blue dots indicating the coordinates. The orange lines show the trajectory when the shutter is off (i.e., resin is not exposed to the laser). Top: ring segments irradiated with one color (375 nm); bottom: ring segments irradiated with two colors (375 nm + 430 nm).

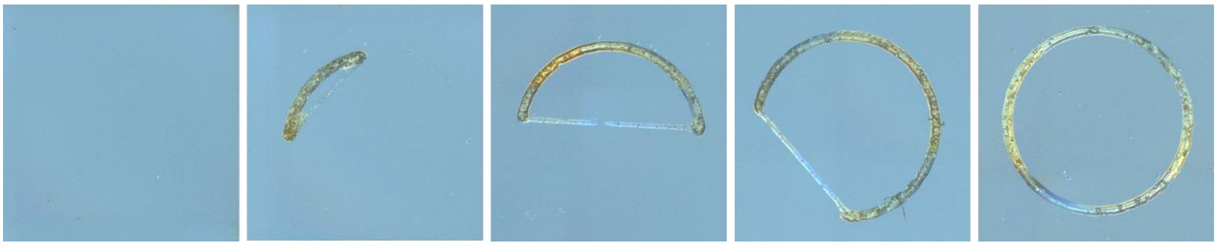

Figure S82. Optical microscopy images of a series of rings with varying fractions (100%, 75%, 50 %, 25 % and 0 %) printed using one-color exposure (375 nm, 5 mW, 0.45 mm s^{-1}), with the remining portions printed using two-color irradiation (375 nm at 5 mW and 430 nm at 10 mW, 0.45 mm s^{-1}). The straight lines were fabricated with two-color irradiation to indicate the two-color pathways.

A butterfly structure was also printed, with the left half fabricated using two colors and the right half using only one color. The G-code files for the butterfly structures are provided in **Supplementary Files 2**. **Figure S83** illustrates the trajectories and coordinates defined in the G-code for each part of the structure.

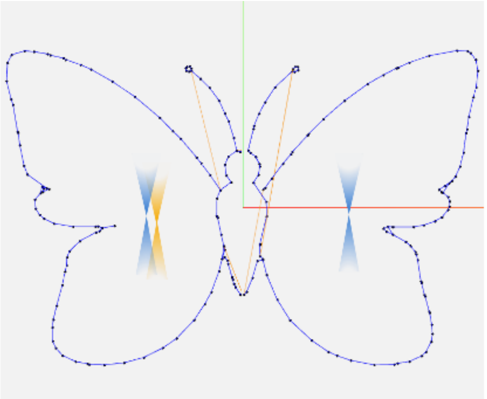

Figure S83. Image illustrates the trajectory of the 2 axes for the butterfly, generated from the G-code files to obtain the corresponding structures.

13 References

[1] H. E. Gottlieb, V. Kotlyar, A. Nudelman, *J. Org. Chem.* **1997**, *62*, 7512.

[2] X. Shen, C. Zhang, F. Lan, Z. Su, Y. Zheng, T. Zheng, Q. Xiong, X. Xie, G. Du, X. Zhao, C. Hu, P. Deng, Z. Yu, *Angew. Chem. Int. Ed.* **2022**, *61*, e202209441.

[3] J. Hobich, F. Feist, P. Werner, J. A. Carroll, O. Fuhr, E. Blasco, H. Mutlu, C. Barner-Kowollik, *Angew. Chem. Int. Ed.* **2025**, *64*, e202413530.

[4] T. N. Eren, F. Feist, K. Ehrmann, C. Barner-Kowollik, *Angew. Chem. Int. Ed.* **2023**, *62*, e202307535.
