# Supplementary material for: Synergistic Two‐Color Photochemical Polymer Network Formation and Lithography
Source: Angew Chem Int Ed Engl. 2025 Oct 3;64(48):e202518815. doi: 10.1002/anie.202518815 (PMC12643345; doi:10.1002/anie.202518815)
Supplement: Supplementary file 4 — Supporting Information [file ANIE-64-e202518815-s004.docx]

**Supplementary file 1:**

**1C 100:** G-code for a 100 % ring printed with 375 nm

**1C 75:** G-code for a 75 % ring printed with 375 nm

**2C 25:** G-code for a 25 % ring printed with 375 nm + 430 nm

**1C 50:** G-code for a 50 % ring printed with 375 nm

**2C 50:** G-code for a 50 % ring printed with 375 nm + 430 nm

**1C 25:** G-code for a 25 % ring printed with 375 nm

**2C 75:** G-code for a 75 % ring printed with 375 nm + 430 nm

**2C 100:** G-code for a 100 % ring printed with 375 nm + 430 nm

**Supplementary file 2:**

**Butterfly left:** G-code for the left half of the butterfly printed with 375 nm + 430 nm

**Butterfly right:** G-code for the right half of the butterfly printed with 375 nm
